# Efficacy of gabapentin for the prevention of postherpetic neuralgia in patients with acute herpes zoster: A double blind, randomized controlled trial

**DOI:** 10.1371/journal.pone.0217335

**Published:** 2019-06-05

**Authors:** Oana Bulilete, Alfonso Leiva, Manuel Rullán, Antonia Roca, Joan Llobera, on behalf of PHN Group

**Affiliations:** 1 Primary Care Research Unit of Mallorca, Balearic Health Service, Palma, Spain; 2 Balearic Islands Health Research Institute (IdISBa), Palma, Spain; 3 Pollença Health Care Centre, Balearic Health Service, Palma, Spain; 4 Son Serra La Vileta Health Care Centre, Balearic Health Service, Palma, Spain; University of Toronto, CANADA

## Abstract

**Background:**

Postherpetic neuralgia (PHN) is the most common complication of herpes zoster (HZ). Previous trials have reported that gabapentin can relieve chronic neuropathic pain, but its effect on prevention of PHN is unclear.

**Objective:**

To assess the efficacy of a 5-week course of gabapentin on acute herpetic pain and on the prevention of PHN at 12 weeks in patients with acute HZ.

**Methods:**

This was a randomized, double blind, placebo-controlled trial conducted in 17 primary care health centers in Mallorca, Spain. All patients were older than 50 years, presented with HZ within 72 h of rash onset, and had moderate-severe pain (≥4 on a 10-point visual analogue scale [VAS]). Ninety-eight patients were randomized to receive gabapentin or placebo. All patients received valaciclovir for 7 days and analgesia if needed. The treatment period was 5 weeks, followed by 7 weeks of follow-up. Gabapentin was initiated at 300 mg/day and gradually titrated to a maximum of 1800 mg/day. The main outcome measure was pain at 12 weeks.

**Results:**

Seventy-five patients completed the study, 33 in the gabapentin group and 42 in the control group. A total of 18.2% of patients in the gabapentin group and 9.5% in the control group reported pain at 12 weeks (*p* = 0.144). Four patients in the gabapentin group (12.1%), but no patients in the placebo group, reported pain of 4 or more on a 10-point VAS. Patients taking gabapentin reported worse health-related quality of life and poorer sleep quality. Three patients discontinued the trial due to adverse effects from gabapentin.

**Conclusion:**

Addition of gabapentin to the usual treatment of HZ within 72 h of rash onset provided no significant relief from acute herpetic pain or prevention of PHN.

**Trial registration:**

ISRCTN Registry identifier: ISRCTN79871784

## Introduction

The most common chronic complication of herpes zoster (HZ; shingles) is postherpetic neuralgia (PHN), a persistent neuropathic pain that occurs after the acute HZ infection. HZ is considered a significant public health problem, due to its increasing incidence along with the ageing population[1] and its substantial impact on patient quality of life, in the acute and chronic phases[2]. Moreover, HZ was recently associated with greater risk for cerebrovascular and cardiac events shortly after the acute infection occurs[3].

Reactivation of varicella zoster virus (VZV), a double-stranded DNA virus responsible for chickenpox, causes HZ. This virus remains dormant within the dorsal root ganglion, and its reactivation, typically in adults older than 50 years[4], leads to unilateral, dermatomal, and painful skin eruptions. The reactivation of VZV is associated with the age-related decrease of cellular immunity to VZV and with impaired cellular immune function [5].

Before the introduction of the universal varicella immunization, the seroprevalence of VZV was estimated to be higher than 95% in people older than 65 year, across 16 European countries according to a recent systematic review[6]; many people are therefore at risk for developing HZ[7]. The incidence of HZ increases with age. The estimated overall incidence is 3.4 to 4.82 per 1000 person-years and increases up to 11 per 1000 person-years in patients aged over 80 years[7]. The lifetime risk ranges between 25% and 30% but is up to 50% for individuals older than 80 years[7].

Estimates for the risk of developing PHN among individuals with HZ range from 5% to more than 30%[8], but there are disagreements about the incidence and prevalence of PHN because there is no universally accepted definition. Thus, a compressive review reported the incidence of PHN was 3.9 to 42.0/100,000 person-years[9]. Previous studies have defined PHN according to the severity of pain (≥3 or 4 on a 10-point Likert scale)[10] or duration (pain persisting for 30, 90, 120, or 180 days)[11]. The main risk factors for PHN are older age, greater acute pain, more widespread rash, presence of prodromal pain, and ophthalmic involvement[8].

Patients with PHN may describe their pain as constant or intermittent, and as burning, aching, throbbing, stabbing, or shooting. Some patients also report allodynia, hyperalgesia and dysesthesia[11]. This long-lasting and debilitating pain may persist for months to years and is often refractory to pharmacological therapies[8].

PHN negatively affects all four health domains—physical, social, functional and psychological[2] and is associated with significant direct and indirect healthcare costs[12]. Moreover, PHN is related with a considerable burden on patients, caregivers, the healthcare system and employers[7].

Researchers and clinicians have proposed many therapies for prevention of PHN, including pharmacological therapies (such as antivirals, amitriptyline, and pregabalin), interventional procedures (epidural and sympathetic nerve blocks), VZV vaccination, and complementary and alternative therapies (such us acupuncture and electrical nerve stimulation). A Cochrane systematic review reported that antivirals, such as acyclovir, may decrease the severity of acute HZ pain at 1 month relative to placebo, but had no significant effect on prevention of PHN [13]. The same review highlighted the need for more studies of valacyclovir and famciclovir and of different subgroups of patients. Another study reported that corticosteroids given during the acute phase of HZ provided no benefit in preventing PHN [14]. There is some evidence that amitriptyline (25 mg per day for 3 months) may decrease the incidence of PHN [15], but further research is needed to confirm the results of this single study.

The only established method for prevention of PHN is the HZ vaccine [10], but the effectiveness of the live attenuated vaccine seems to decline over time [16]. A new recombinant zoster vaccine (Zostavax) was recently approved for adults who are 50 or more years-old, and this new vaccine seems more effective for prevention of HZ and PHN [17].

Gabapentin is an anticonvulsant initially developed and approved as adjunctive therapy for treatment of partial seizures, often used for the treatment of neuropathic pain[18]. It is an analogue of gamma aminobutyric acid and binds to the α_2_-δ site of voltage-dependent calcium channels, thereby reducing neurotransmitter release [18]. A previous study reported that gabapentin reduced acute herpetic pain and delayed postherpetic pain in mice [19]. Studies in humans demonstrated gabapentin was beneficial for the treatment of chronic neuropathic pain and might also reduce allodynia and hyperalgesia[20]. The European consensus-based guideline on the management of HZ recommends that gabapentin may be added to analgesics for treatment of HZ infection if moderate or severe pain is present [21].

The objective of this study of patients older than 50 years with moderate or severe pain from HZ was to assess the efficacy of gabapentin added to the usual treatment (valacyclovir and analgesics as needed) on reducing acute pain and preventing PHN at 12 weeks.

## Materials and methods

### Design and setting

A full description of the research protocol was published previously[22]. This was a multicenter, parallel, randomized, double-blind, placebo-controlled trial of patients from 17 primary health care centers in Mallorca (Spain) that was conducted from February 2014 to January 2017. This study followed the principles outlined in the Declaration of Helsinki. The study protocol was also approved by the Primary Care Research Committee of the Balearic Ethical Committee of Clinical Research (IB 1857/12), and the Spanish Agency on Drugs and Medical Devices (Agencia Española de Medicamentos y Productos Sanitarios). The trial was registered on ISRCTN (Registry identifier: ISRCTN79871784) on May 2, 2013 (http://www.isrctn.com/ISRCTN79871784). Before inclusion in the trial, each patient signed a written informed consent document.

### Patients

**Fig 1:**
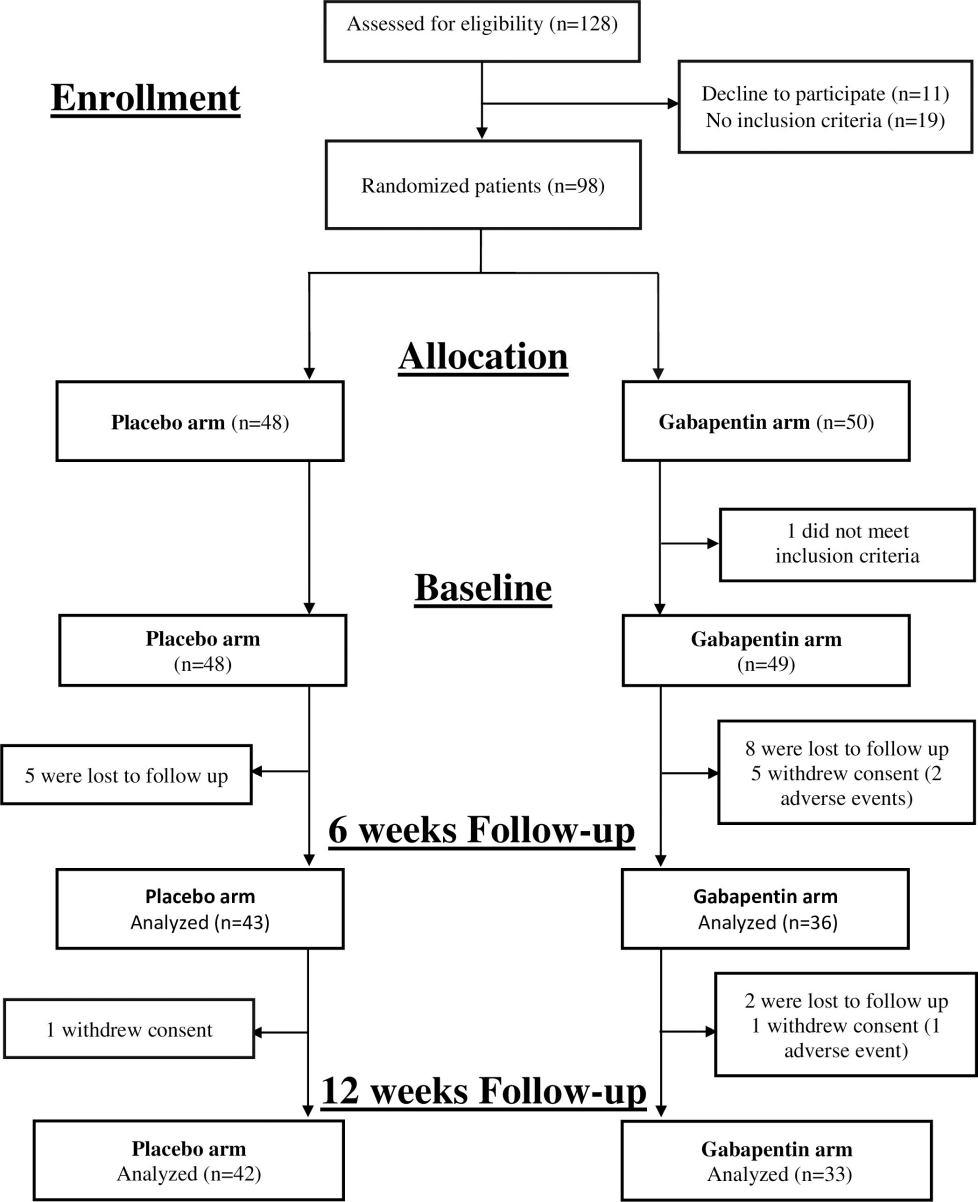
Study design and patient disposition (CONSORT flow diagram).

All patients presenting with acute herpetic rash at one of the participating centers were assessed and referred to the collaborating general practitioner (GP), who determined if the inclusion criteria were satisfied. If so, the patient was asked to sign an informed consent document. All included patients were older than 50 years, had uncomplicated HZ presenting within the first 72 h of vesicle formation, and had an average pain score of at least 4 of 10 on a visual analogue scale (VAS) before therapy (**Fig 1**).

Patients were excluded if they were taking gabapentin or a tricyclic antidepressant; had evidence of cutaneous or visceral dissemination or ocular involvement; had a history of intolerance or hypersensitivity to any active components or excipients in the study drugs; had severe hepatic impairment or impaired renal function; received cytotoxic drugs or immunosuppressive therapy within the previous 3 months (e.g., long-term systemic corticosteroids); were diagnosed with any immune dysfunction; used immunomodulatory medications (including interferon) within the previous 4 weeks; or received a VZV vaccine.

### Treatment

Participants were randomly allocated to receive gabapentin or placebo by use of a computer-generated randomization list in blocks of six. The randomization and allocation process were described in a previous publication[22]. The participants, the collaborating GP, the research staff, and the investigators who assessed outcomes were all blinded to treatment allocations. At the final visit, to assess the effectiveness of blinding, the patients were asked to guess whether they received the treatment or placebo. The trial period was 12 weeks, and the patients were scheduled for 5 visits (baseline, week-1, -4, -6, and -12). At baseline, sociodemographic data (age, sex, weight, and height) and clinical data (significant medical history, concomitant use of other medications) were collected. The rash localization, pain intensity, quality of life and sleep patterns were also recorded.

All participants received 1000-mg caplets of valacyclovir hydrochloride 3 times daily for 7 days, and an analgesic according to the World Health Organization three-step pain relief ladder (nonopioids, mild opioids, then strong opioids) [23]. At the baseline visit, patients received one bottle of 300-mg gabapentin pills or matching placebos.

**Fig 2:**
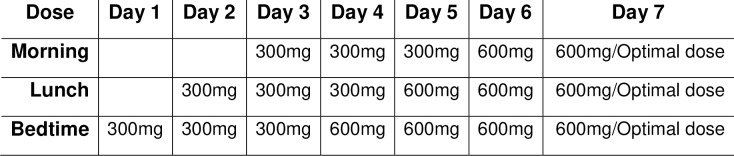
Dose titration scheme during the 1^st^ week of the study.

The treatment period was 5 weeks. Gabapentin was initiated at 300 mg/day at breakfast, and the dose increased by 300 mg/day up to a ceiling dose of 1800 mg/day by day 7 (**Fig 2**). The dose was increased regardless of whether efficacy was achieved at a lower dose. Among patients who developed adverse effects, the dose was reduced to the previously tolerated level. Gabapentin (at 1800 mg/day or the optimal dose established during the titration period) was maintained for 3 weeks and was followed by dose-tapering for a maximum of 1 week. Patients receiving the placebo received the same dose escalation and the same number of capsules. Patients were not allowed to receive tricyclic antidepressants or systemic corticosteroids during the trial.

Excerpt of the study medication dose titration scheme based on a dose of 1800mg/day. Treatment was maintained for 3 weeks, followed by a 1 week of dose tapering.

### Outcome measures

The primary outcome was the efficacy of gabapentin on reducing PHN, based on a 10-point VAS in which 0 indicated no pain and 10 indicated the worst possible pain. PHN was considered present for any score above 0 at week-12.

The secondary outcome measures were:

*(i)* moderate-severe pain (VAS ≥ 4) at week-12;
*(ii)* neuropathic pain based on a validated Spanish version of the DN4 questionnaire (Doleur Neuropathique) [24] at baseline and week-12;
(iii) any pain (VAS score > 0) at week-4 and week-6;
*(iv)* 50% reduction of pain relative to baseline[25], measured using a VAS;
*(v)* quality of life based on the ShortForm-12 (SF-12) [26] at baseline, week-4, and week-12;
*(vi)* sleep interference based on the Medical Outcomes Study Sleep Scale (MOS-Sleep) [27] at baseline, week-4, and week-12;
*(vii)* patient global impression of change scale (PGIC) [28] score at week-12;
*(viii)* analgesic consumption (recorded at every visit);
and *(ix)* safety and adverse effects (recorded at every visit).

### Statistical analysis

An initial estimate indicated that a sample size of 134 would be required for a statistical power of 80% and detection of a difference of at least 25% in the incidence of PHN. Thus, a 12-month recruitment period was planned, with an anticipated loss to follow-up of 20%. Primary analyses were performed on an intention-to-treat basis. All randomized patients, regardless of whether they received the treatment or were lost to follow up, were analyzed by use of multiple imputation methods that considered treatment group, age, sex, HZ location, and VAS pain score at baseline.

Logistic regression models, which adjusted for age, sex, and HZ location, were performed to analyze the effectiveness of gabapentin treatment by measurement of *(i)* number of patients reporting any pain (VAS > 0) at week-12; *(ii)* pain ≥ 4 on the VAS or based on DN-4 score, *(iii)* any pain (VAS > 0) at week-6; and *(iv)* pain reduction of 50% or more at week-12. Between-group comparisons of sleep quality (measured by the MOS-Sleep questionnaire) and quality of life (measured by the SF-12 questionnaire) at week-12 were analyzed by Student’s *t*-test. All treatment effects were considered significant for a two-sided *p*-value of 0.05.

The results of analyses according to intention to treat and per protocol were compared. The per protocol analysis included 2 different analyses: effectiveness of gabapentin among patients completing the trial, and effectiveness of gabapentin among those taking at least 300 mg gabapentin or placebo per day.

The effectiveness of patient blinding in each treatment group was evaluated using the James blinding index, applying the equation provided by the authors[29]. This index is a variation of the kappa coefficient and ranges between 0 to 1, in which 0 indicates total absence of blinding, 1 indicates complete blinding, and 0.5 indicates completely random [30]. The participants were asked to guess to which group they were designated, by choosing one of the following answers: (i). gabapentin or (ii). placebo or (iii). "don’t know". Successful blinding was considered if the upper bound of the confidence interval (CI) of the index was above 0.5.

We performed a between-group comparisons of reported pain at week-1, -4, -6, and -12, the treatment groups were compared using an analysis of covariance, adjusted for age, sex, and HZ location. A Chi-square test was used for analysis of PGIC scores and presence of optimal sleep (yes/no) on the MOS-Sleep scale.

## Results

The 29 participating GPs initially screened 128 eligible patients (**Fig 1**). Ninety-eight consenting patients were ultimately randomized, 50 to the intervention group and 48 to the placebo group. After randomization, 15 patients (15.3%) were loss to follow up (10 in the gabapentin group and 5 in the placebo group) and 7 patients (7.14%) withdrew consent (6 in the gabapentin group and 1 in the placebo group). One patient randomized to the gabapentin group was excluded from the analysis, for not meeting inclusion criteria. Adverse effects from gabapentin were responsible for 3.1% of the patient losses (3 of 6 patients who withdrew consent).

The two groups had similar demographic and clinical characteristics at baseline (Table 1). Overall, the mean patient age at enrolment was 65 years, 54.1% of the patients complained of moderate pain (4 to 6.9 on a VAS), and 45.9% complained of severe pain (7 to 10 on a VAS).

**Table 1:** Baseline characteristics of patients in the two groups.

	Gabapentin (n = 49)	Placebo(n = 48)
	n/N (%)	n/N (%)
**Age (years)**	65.1±11.4	66.0±11.1
• <65	27/49 (55.1)	23/48 (47.9)
• 65–84	20/49 (40.8)	25/48 (52.1)
• >85	2/49 (4.1)	0/48 (0)
Women	28/48 (58.3)	30/48 (62.5)
BMI (kg/m^2^)	28.1±4.7	27.3±5.7
**HZ Location**		
• Thorax	26/49 (53.1)	19/48 (39.6)
• Abdomen	7/49 (14.3)	7/48 (14.6)
• Extremities	9/49 (18.4)	10/48 (20.8)
• Other	7/49 (14.3)	12/48 (25.0)
**Pain VAS**	**Mean (SD)** 7.0±1.8	**Mean (SD)** 6.6±1.8
**SF-12**	**Mean (SD)**	**Mean (SD)**
• Physical functioning	45.6±12.1	48.9±10.6
• Role Physical	26.9±3.6	27.6±3.4
• Role emotional	20.1±4.5	20.2±4.3
• Mental Health	48.3±11.4	51.1±11.7
• Bodily pain	50.2±11.7	48.2±11.5
• General Health	41.8±11.2	43.3±11.2
• Vitality	53.1±11.7	54.5±11.5
• Social functioning	50.2±9.0	49.0 ±11.0
• Summary Scale Physical	44.3±9.8	45.1±7.6
• Summary Scale Mental	40.9±8.7	41.7±9.8
**MOS- Sleep**	**Mean (SD)**	**Mean (SD)**
• Sleep disturbance	33.7±26.5	34.6±30.4
• Snoring	58.5±38.0	43.8±38.3
• Awakening with short breath or headache	11.2±20.5	6.5±20.5
• Sleep adequacy	68.3±32.6	73.0±28.0
• Day-time somnolence	31.9 ±19.9	31.9±22.4
• Sleep Problems Index	25.7±18.8	24.2±22.9
**MOS Sleep: optimal sleep**	21/43 (48.8)	23/41 (56.1)

Abbreviations: BMI: Body Mass Index; VAS: visual analogic scale; SD: standard deviation; SF-12: Short-Form 12; MOS- Sleep: Medical Outcomes Study.

The maximum allowed daily dosage of gabapentin (1800 mg) was achieved in 9 of 33 patients (27.3%). The mean adherence was 97.5% in the placebo group and 74.8% in the gabapentin group. The major side effects in the gabapentin group were dizziness (10 patients), somnolence (8 patients), and abdominal pain (5 patients); the major side effects in the placebo group were also dizziness (4 patients), somnolence (4 patients), and abdominal pain (2 patients).

### Primary outcomes

At week-12, 10 patients overall reported at least some pain (VAS > 0), 4 in the placebo group and 6 in the gabapentin group (Table 2). This difference was not statistically significant, p-value = 0.144, OR (95% confidence interval [95%CI]) = 2.59(0.59–11.28).

**Table 2:** Postherpetic neuralgia pain scores at week-12 (primary outcomes) and week-6 (secondary outcome) in the two groups.

	Gabapentin n/N (%)	Placebo n/N (%)	OR (95% CI)*	p-Value	Gabapentin >300 mg/day n/N (%)	Placebo	OR (95% CI)* >300 mg/day n/N (%)	p-Value	ITT analysis Imputed OR (95% CI)	p-Value
**Primary outcomes**										
Pain VAS > 0 at week-12	6/33 (18.2)	4/42 (9.5)	2.59 (0.59–11.28)	0.144	3/24 (12.5)	3/32 (9.4)	1.58 (0.25–10.02)	0.627	2.61 (0.43–15.8)	0.283
Pain VAS ≥ 4 at week-12	4/33 (12.1)	0/42 (0)	N/A	N/A	2/24 (8.3)	0/32 (0)	N/A	N/A	N/A	N/A
DN-4 ≥4 at week-12	4/32 (12.5)	3/37 (8.1)	1.55 (0.28–8, 61)	0.827	2/24(8.3)	3/29 (10.3)	0.74 (0.10–5.30)	0.740	1.54 (0.23–10.4)	0.735
**Secondary outcome**										
Pain VAS > 0 at week-6	10/33 (30.3)	6/40 (15.0)	1.94 (0.57–6.53)	0.287	6/24 (25.0%)	3/32 (9.4)	2.8 (0.56–14.56)	0.205	2.42 (0.63–9.25)	0.188

Abbreviations: OR: odd ratio; 95% CI: 95% confidence interval; ITT: intention to treat; VAS: visual analogic scale; DN4: Doleur Neuropathique in 4 Questions.

*Adjusted by age, sex, and herpes zoster location.

Three patients in the gabapentin group and no patients in the placebo group reported pain greater than 4 on the VAS atweek-12.

The two groups had no significant difference in the incidence of neuropathic pain, based on the DN-4 questionnaire at week-12 (placebo group: 10.8%; gabapentin group: 12.5%). Moreover, most patients did not report any pain on the pain VAS at week-12 (Table 3). For patients reporting pain at week-12, most reported only mild pain, and only 3 patients reported moderate pain (4 to 6.9 on a VAS). Gabapentin had no effect on average HZ pain at any time (Table 3). Although both groups experienced reductions in pain over time, this change was larger and statistically significant in the placebo group at week-4, -6, and -12.

**Table 3:** Average herpes zoster pain score at week-1, -4, -6, and -12 in the two groups.

	Gabapentin	Placebo	Difference	
Time	Pain VAS	Pain VAS	Difference	p-value
	Adjusted mean (95% CI)	Adjusted mean (95% CI)	(95% CI)	
1 week	3.9 (3.1–4.8)	3.5 (2.6–4.3)	0.5 (-0.7–1.6)	0.451
4 weeks	2.2 (1.4–2.9)	0.8 (0.1–1.5)	1.4 (0.3–2.4)	**0.009**
6 weeks	1.2 (0.7–1.7)	0.3 (-0.1–0.8)	0.9 (0.2–1.6)	**0.014**
12 weeks	0.8 (0.4–1.3)	0.1 (-0.3–0.5)	7.8 (1.7–13.9)	**0.013**

Abbreviations: 95%CI: 95% confidence interval; VAS: visual analogic scale.

### Response rate

At 6 weeks, 10 patients in the gabapentin group and 6 in the placebo group reported some pain (VAS > 0). This difference was not statistically significant (Table 2). Comparison of pain at baseline and week-12 indicated most patients in the gabapentin group (30/33) had a 50% or greater reduction of pain, but all 42 patients in the placebo group had a 50% or greater reduction of pain (*p* = 0.046).

### Sleep interference

Patients in the gabapentin group reported more sleep problems, with significantly more sleep disturbances, awakening with shortness of breath or headache, sleep inadequacy, and worse score on the Sleep Problems Index (Table 4).

**Table 4:** Sleep problems and quality of life at week -12 in the two groups.

	Gabapentin	Placebo	p-Value
	Mean (SD)	Mean (SD)	
**MOS-Sleep **			
Sleep disturbance	32.7±21.7	22.8±26.6	**0.028**
Snoring	49.7±33.4	46.2±41.3	0.759
Awakens short of breath or headache	16.1±25.5	5.0±12.6	**0.032**
Sleep adequacy	70.3±26.2	78.7±26.2	**0.005**
Day-time somnolence	35.9±22.5	24.2±20.1	0.126
Sleep problems Index	25.4±19.8	17.4±15.6	**0.038**
**MOS-Sleep: optimal sleep**	n/N (%)	n/N (%)	0.392
	19/33 (57.6)	20/42 (47.6)	
**SF-12**			
Physical functioning	44.9±11.8	51.5±7.5	**0.005**
Role Physical	26.1±4.1	28.7±2.3	**0.001**
Role emotional	18.7±5.2	21.8±2.6	**0.001**
Mental Health	45.3±10.2	53.7±8.1	**<0.001**
Bodily pain	47.6±12.5	53.1±7.9	**0.026**
General Health	43.4±10.8	45.8±9.4	0.315
Vitality	50.9±10.7	54.8±9.4	0.105
Social Functioning	47.8±11.1	49.5±11.2	0.508
Summary Scale Physical	44.1±9.9	47.8±6.6	0.061
Summary Scale Mental	38.1±9.0	42.6±6.5	**0.018**

Abbreviations: SD: standard deviation; SF-12: Short-Form 12; MOS-Sleep: Medical Outcomes Study.

### Quality of life

The health status of patients, measured by SF-12, was worse in the gabapentin group than the placebo group (Table 4). In particular, the gabapentin group had lower scores for physical functioning, role physical, role emotional, mental health, composite scale score, and bodily pain. We also analyzed quality of life and sleep problems, comparing patients with or without pain at week -12; statistically significant differences were observed in both physical and mental summary scale (Table 5).

**Table 5:** Sleep problems and quality of life in patient with pain score >0 at week -12.

	Patient without pain	Patient with pain score >0	p-Value
	Mean (SD)	Mean (SD)	
**MOS-Sleep **			
Sleep disturbance	26.5±25.2	30.4±22.1	0.662
Snoring	46.2±37.9	57.8±38.0	0.414
Awakens short of breath or headache	8.4±19.0	20.0±24.5	0.103
Sleep adequacy	74.2±29.7	81.1±16.9	0.323
Day-time somnolence	27.8±20.3	39.3±30.1	0.144
Sleep problems Index	20.6±18.2	23.0±16.5	0.719
**MOS-Sleep: optimal sleep**	n/N (%)	n/N (%)	0.077
	31/62 (50.0)	8/10 (80.0)	
**SF-12**			
Physical functioning	49.4±9.8	43.6±11.6	0.162
Role Physical	28.0±3.1	24.9±4.3	0.057
Role emotional	21.0±3.7	16.9±5.9	**0.004**
Mental Health	51.0±9.5	43.8±10.8	**0.034**
Bodily pain	51.7±9.2	44.2±16.0	**0.026**
General Health	46.3±9.1	35.0±10.9	**0.010**
Vitality	53.9±9.3	47.7±13.4	0.074
Social Functioning	50.5±9.4	37.4±14.6	**0.020**
Summary Scale Physical	47.1±7.9	40.6±9.6	**0.023**
Summary Scale Mental	41.7±7.8	34.1±5.4	**0.004**

Abbreviations: SD: standard deviation; SF-12: Short-Form 12; MOS-Sleep: Medical Outcomes Study.

### Patient global impression of change

When asked about their global impression of change using the PGIC, 29 of 32 patients (90.6%) in the gabapentin group reported much improvement, and 38 of 40 patients (95.0%) in the placebo group reported much improvement. This difference was not statistically significant.

### Analgesic use

Analgesics were prescribed to 32.7% of the patients during the 12 week study period. There was less use of rescue analgesia in the gabapentin group (26% *vs*. 40%), but this was not statistically significant. The most prescribed drugs were analgesics and antipyretics (36%, ATC code: N02B), followed by non-steroidal anti-inflammatory drugs (13%, ATC code: M01A), and opioids (10.7%, ATC code: N02A).

### Assessment of blinding

At week-12, all patients were asked to guess the group to which they were randomized. Most patients in each group believed they were in the gabapentin group. The James index of patient blinding was 0.62 (95% CI: confidence interval (CI)95% (0.54 to 0.70), which was considered a successful random blinding.

### Loss to follow-up

Most of the patients lost to follow-up were men, and more patients were lost to follow-up in the gabapentin group (n = 16) than in the placebo group (n = 6). Most of these 16 patients in the gabapentin group abandoned the study between the first and the second visit (week-2 to week-4). Patients lost to follow-up reported less pain at week-4 (VAS 1.4 *vs*. 0.2) and this difference was statistically significant (Table 6). Thus, patients who had mild pain or were pain-free withdrew from more frequently than those continuing the trial.

**Table 6:** Characteristics of patients who completed the trial and were analyzed, and of patients who withdrew.

	Analyzed	Drop-outs	p-value
	n/N (%)	n/N (%)	
**Age**			
<65 years	35/75 (46.7)	16/23 (69.6)	0.077
65–84 years	39/75 (52.0)	6/23 (26.1)	
>85 years	1/75 (1.3)	1/23 (4.3)	
Mean age (SD)	65.6±10.6	64.2±11.8	0.583
Women	47/75 (62.7)	7/23 (30.4)	0.007
Mean BMI (kg/m^2^) (SD)	28.0±5.5	27.7±4.2	0.888
**HZ Location**			
Thorax	36/75 (48.0)	9/23 (39,1)	0.330
Abdomen	8/75 (10.7)	6/23 (26.1)	
Extremities	15/75 (20.0)	4/23 (17.4)	
Other	16/75 (21.3)	4/23 (17.4)	
**SF-12** Mean (SD)			
Physical functioning	47.3±11.5	46.9±11.7	0.925
Role Physical	27.4±3.4	26.4±3.9	0.454
Role emotional	19.8±4.5	22.5±0.1	0.081
Mental Health	49.4±11.9	52.3±7.4	0.476
Bodily pain	48.7±11.7	52.9±10.3	0.310
General Health	42.6±11.5	42.5±8.1	0.994
Vitality	54.1±11.9	52.2±8.9	0.656
Social functioning	49.4±10.2	51.0±8.9	0.669
Summary Scale Physical	44.7±8.5	44.3±10.4	0.898
Summary Scale Mental	41.1±9.6	43.4±5.8	0.474
**MOS- Sleep** Mean (SD)			
Sleep disturbance	34.3 ±28.8	32.6±26.3	0.864
Snoring	50.4±38.8	55.0±38.2	0.752
Awakening with short breath or headache	9.3±18.9	4.4±8.8	0.450
Sleep adequacy	71.5±29.7	64.4±36.1	0.514
Day-time somnolence	32.5±26.7	26.7±17.3	0.434
Sleep Problems Index I	24.8 ±21.4	25.9±17.1	0.880
Sleep Problems Index II	27.2±21.2	26.1±16.0	0.877
**MOS Sleep: optimal sleep**	39/75 (52.0)	5/9 (55.6)	0.840
**VAS pain** Mean (SD)			
VAS-pain at baseline	6.8±1.9	6.7±1.7	0.851
VAS-pain at week 1	3.8±2.8	2.2±2.1	0.071
VAS-pain at week 4	1.4±2.0	0.2±0.4	0.001
VAS-pain at week 6	0.7±1.5	0.6±1.2	0.920

Abbreviations: BMI: Body Mass Index; VAS: visual analogic scale; SD: standard deviation; SF-12: Short-Form 12; MOS- Sleep: Medical Outcomes Study.

### Safety

There were two severe adverse events during follow-up. One patient had a stroke and one patient had syncope and trifascicular heart block. Both events were in the gabapentin group and both patients were successfully treated. Neither event was considered related to gabapentin use.

## Discussion

We found no evidence that gabapentin prevented PHN when added to the usual treatment for HZ. Some evidence suggests that attenuation of pain during the acute phase of HZ might prevent PHN[31], and that gabapentin attenuates neuropathic pain by acting on the central and peripheral nervous system[32], both of which are damaged by the HZ infection. Therefore, we hypothesized that addition of gabapentin to the usual HZ treatment might provide better control of pain and reduce the incidence of PHN. Our results are inconsistent with this hypothesis.

The efficacy of gabapentin for the treatment of PHN is well established and there is also evidence that it improves patient quality of life, mood and sleep[11]. Typical gabapentin treatment begins at 300 mg/day and increases to 3600 mg/per day if necessary[33]. A recent Cochrane review reported that among patients taking gabapentin at doses higher than 1200 mg/day, there was a substantial effect on PHN-related pain (50% or more reduction in pain) in 32% of patients, and a moderately beneficial effect (30% or more reduction in pain) in 46% of patients[34]. However, these therapeutic dose levels are rarely achieved, and one estimate is that only 14% of patients receive a minimally effective dose of gabapentin[35]. Moreover, a 10 week titration period may be necessary to achieve these high doses[35]. Our results indicated that 27.3% of the participants reached the maximal dose of gabapentin. However, gabapentin was less effective than placebo at doses as high as 1800 mg, even though we used a rapid titration regimen to achieve a better pain control during the acute phase of HZ.

Another study that evaluated the efficacy of gabapentin during the acute herpetic infection phase had results similar to ours[36]. This RCT examined 120 participants with HZ within 4 days of rash onset and with pain scores of 4 on a 10-point Likert scale. In this study, 60 patients were randomized to receive 900 mg gabapentin (300 mg *tid*). The results indicated that the two groups had no significant differences of mean pain scores at each point of the trial, nor in the incidence of PHN (defined as a persistent pain score of 4 or more) after 12 weeks.

An open label pilot study of 133 patients who received gabapentin up to 3600 mg/day during the acute phase of HZ, reported a lower incidence of PHN relative to previously published data of patients not receiving gabapentin[37]. However, this study had no control group, so the clinical significance of the results is questionable.

Another RCT reported that when gabapentin is given during the acute phase of HZ infection, it provided no more relief from pain than a placebo at 4 weeks since rash onset[38]. Nevertheless, other authors reported beneficial effects of gabapentin on acute herpetic pain[39–41].

We also found that gabapentin did not improve QoL (measured by the SF-12) or sleep quality (measured by the MOS- sleep scale). This is similar to the results of a previous trial, which reported that gabapentin had no significant effect on Dermatological Quality of Life scores [36]. In our study, patients who received gabapentin had significantly poorer scores in the SF-12 domains of physical functioning, role physical, role emotional, mental health, and bodily pain. In addition, patients taking gabapentin had significantly poorer scores for sleep disturbance, sleep adequacy, and awakening with shortness of breath or a headache.

There were no significant safety concerns during our trial. Dizziness, somnolence, and abdominal pain were the most common adverse effects, similar to other trials of gabapentin. Only 3 patients in the gabapentin group withdrew consent due to drug-related adverse effects.

We found that gabapentin had no effect on acute pain during an HZ infection. These results are important, because gabapentin is recommended during the acute phase of HZ[21]. Clinicians must be aware of the side effects of gabapentin, such as dizziness, sedation, worsening of cognitive impairment, and ataxia, and the growing evidence that some patients may abuse misuse gabapentin due to its euphoric effects[42]. Furthermore, older patients are more likely to receive long-term treatments and polypharmacy, so extra caution is needed to avoid adverse interactions in this group.

An estimated 1 of 3 people develop HZ at some time during their lives[43]. When established, PHN is often very difficult to treat, and typically requires a multidisciplinary approach. As far as we know, there is no evidence that any therapy can prevent PHN after the onset of HZ. Vaccination seems to be key for prevention of HZ, and implicitly PHN. Recently, the Advisory Committee on Immunization Practices recommended vaccination for immunocompetent adults aged ≥50 years, preferably with the recombinant zoster vaccine and irrespective of prior receipt of any varicella vaccine or zoster live vaccine[44]. Despite the widespread availability of the live VZV vaccine, Centers for Disease Control and Prevention estimated that only 33% of adults older than 60 years had received HZ vaccine in the United States[44].

One strength of our study is that we examined the effectiveness of masking by asking all patients to guess whether they received gabapentin or the placebo. Most patients in the placebo group believed they were assigned to the gabapentin group. Another strength of our study is that its setting was in primary care offices, the type of health care facility most visited by patients. Providing an inexpensive and established therapy for pain control during the acute phase of HZ and for prevention of PHN in primary care is especially important, due to the limited accessibility to other analgesic therapies, such as sympathetic nerve blocks and pulsed radiofrequency electrical stimulation.

However, our trial had several limitations. First, we had a lower rate of recruitment. To achieve the recruitment target, based on our calculations of sample size, we extended the inclusion period an additional 6 months. We also included more primary health care centers than originally planned, as well as an off-hours primary care service and the emergency centers of two hospitals. We sent quarterly e-mail reminders describing the project inclusion criteria to all the collaborating centers, and also sent biannual updates. Other studies also reported difficulties in recruiting patients with acute HZ[45]. Nevertheless, our results must be interpreted carefully due to the smaller than expected sample size.

Secondly, in the gabapentin group 10 of 98 patients were lost to follow up and 6 participants withdrew consent. However, we attempted to reduce the possibility of attrition bias by performing an intention to treat analysis.

## Conclusion

Our results provide no evidence that gabapentin prevented PHN. However, further studies with larger sample sizes are needed to confirm the effect of gabapentin on prevention of PHN.

## Supporting information

S1 Appendix CONSORT checklist. (DOC)

S2 Appendix Study protocol original language. (DOC)

S3 Appendix Study protocol. (DOCX)

## References

[1] Kawai K, Gebremeskel BG, Acosta CJ. Systematic review of incidence and complications of herpes zoster: towards a global perspective. BMJ Open. 2014 6 10;4(6):e004833–e004833. 10.1136/bmjopen-2014-004833 24916088 PMC4067812

[2] Johnson RW, Bouhassira D, Kassianos G, Leplège A, Schmader KE, Weinke T. The impact of herpes zoster and post-herpetic neuralgia on quality-of-life. BMC Med. 2010 12 21;8(1):37. 20565946 10.1186/1741-7015-8-37 PMC2905321

[3] Erskine N, Tran H, Levin L, Ulbricht C, Fingeroth J, Kiefe C, et al A systematic review and meta-analysis on herpes zoster and the risk of cardiac and cerebrovascular events. PLoS One. 2017 7 27;12(7):e0181565 10.1371/journal.pone.0181565 28749981 PMC5531458

[4] Gan EY, Tian EAL, Tey HL. Management of herpes zoster and post-herpetic neuralgia. Am J Clin Dermatol. 2013 4 2;14(2):77–85. 10.1007/s40257-013-0011-2 23456596

[5] Yawn BP, Gilden D. The global epidemiology of herpes zoster. Neurology. 2013 9 3;81(10):928–30. 10.1212/WNL.0b013e3182a3516e 23999562 PMC3885217

[6] Bollaerts K, Riera-Montes M, Heininger U, Hens N, Souverain A, Verstraeten T, et al A systematic review of varicella seroprevalence in European countries before universal childhood immunization: deriving incidence from seroprevalence data. Epidemiol Infect. 2017;145(13):2666–77. 10.1017/S0950268817001546 28826422 PMC5647669

[7] Johnson RW, Alvarez-Pasquin M-J, Bijl M, Franco E, Gaillat J, Clara JG, et al Herpes zoster epidemiology, management, and disease and economic burden in Europe: a multidisciplinary perspective. Ther Adv Vaccines. 2015 Jul 19;3(4):109–20. 10.1177/2051013615599151 26478818 PMC4591524

[8] Forbes HJ, Thomas SL, Smeeth L, Clayton T, Farmer R, Bhaskaran K, et al A systematic review and meta-analysis of risk factors for postherpetic neuralgia. Pain. 2015 1;157(1):30–54. 10.1097/j.pain.0000000000000307 PMC4685754 26218719

[9] van Hecke O, Austin SK, Khan RA, Smith BH, Torrance N. Neuropathic pain in the general population: A systematic review of epidemiological studies. Pain. 2014 4;155(4):654–62. 10.1016/j.pain.2013.11.013 24291734

[10] Johnson RW, Rice ASC. Postherpetic Neuralgia. N Engl J Med. 2014 10 16;371(16):1526–33. 10.1056/NEJMcp1403062 25317872

[11] Tontodonati M, Ursini T, Polilli E, Vadini F, Di Masi F, Parruti G. Post-herpetic neuralgia. Int J Gen Med. 2012 10;5:861 10.2147/IJGM.S10371 23109810 PMC3479946

[12] van Wijck AJM, Aerssens YR. Pain, itch, quality of life, and costs after herpes zoster. Pain Pract. 2017 7;17(6):738–46. 10.1111/papr.12518 27611885

[13] Li Q, Chen N, Yang J, Zhou M, Zhou D, Zhang Q, et al Antiviral treatment for preventing postherpetic neuralgia In: He L, editor. Cochrane Database of Systematic Reviews. Chichester, UK: John Wiley & Sons, Ltd; 2009 p. CD006866 10.1002/14651858.CD006866.pub2 19370655

[14] Chen N, Yang M, He L, Zhang D, Zhou M, Zhu C. Corticosteroids for preventing postherpetic neuralgia In: He L, editor. Cochrane Database of Systematic Reviews. Chichester, UK: John Wiley & Sons, Ltd; 2010 p. CD005582 10.1002/14651858.CD005582.pub3 21154361

[15] Bowsher D. The effects of pre-emptive treatment of postherpetic neuralgia with amitriptyline: a randomized, double-blind, placebo-controlled trial. J Pain Symptom Manage. 1997 6;13(6):327–31. 9204652 10.1016/s0885-3924(97)00077-8

[16] Morrison VA, Johnson GR, Schmader KE, Levin MJ, Zhang JH, Looney DJ, et al Long-term persistence of zoster vaccine efficacy. Clin Infect Dis. 2015;60(6):900–9. 10.1093/cid/ciu918 25416754 PMC4357816

[17] Lal H, Cunningham AL, Godeaux O, Chlibek R, Diez-Domingo J, Hwang S-J, et al Efficacy of an Adjuvanted Herpes Zoster Subunit Vaccine in Older Adults. N Engl J Med. 2015;372(22):2087–96. 10.1056/NEJMoa1501184 25916341

[18] Kremer M, Salvat E, Muller A, Yalcin I, Barrot M. Antidepressants and gabapentinoids in neuropathic pain: Mechanistic insights. Neuroscience. 2016 12 3;338:183–206. 10.1016/j.neuroscience.2016.06.057 27401055

[19] Kuraishi Y, Takasaki I, Nojima H, Shiraki K, Takahata H. Effects of the suppression of acute herpetic pain by gabapentin and amitriptyline on the incidence of delayed postherpetic pain in mice. Life Sci. 2004 4 9;74(21):2619–26. 10.1016/j.lfs.2004.01.005 15041444

[20] Patel R, Dickenson AH. Mechanisms of the gabapentinoids and α 2 δ-1 calcium channel subunit in neuropathic pain. Pharmacol Res Perspect. 2016 4;4(2):e00205 10.1002/prp2.205 27069626 PMC4804325

[21] Werner RN, Nikkels AF, Marinović B, Schäfer M, Czarnecka-Operacz M, Agius AM, et al European consensus-based (S2k) Guideline on the Management of Herpes Zoster—guided by the European Dermatology Forum (EDF) in cooperation with the European Academy of Dermatology and Venereology (EADV), Part 2: Treatment. J Eur Acad Dermatology Venereol. 2017 1;31(1):20–9. 10.1111/jdv.13957 27579792

[22] Rullán M, Bulilete O, Leiva A, Soler A, Roca A, González-Bals MJ, et al Efficacy of gabapentin for prevention of postherpetic neuralgia: study protocol for a randomized controlled clinical trial. Trials. 2017 12 14;18(1):24 10.1186/s13063-016-1729-y 28088231 PMC5237496

[23] World Health Organization. WHO’s cancer pain ladder for adults [Internet]. World Health Organization; 1986 Available from: http://www.who.int/cancer/palliative/painladder/en/

[24] Perez C, Galvez R, Huelbes S, Insausti J, Bouhassira D, Diaz S, et al Validity and reliability of the Spanish version of the DN4 (Douleur Neuropathique 4 questions) questionnaire for differential diagnosis of pain syndromes associated to a neuropathic or somatic component. Health Qual Life Outcomes. 2007 12 4;5(1):66. 18053212 10.1186/1477-7525-5-66 PMC2217518

[25] European Medicines Agency. Guideline on the clinical development of medicinal products intended for the treatment of pain [Internet]. 2016 Available from: http://www.ema.europa.eu/ema/index.jsp?curl=pages/regulation/general/general_content_001161.jsp&mid=WC0b01ac0580034cf5

[26] Vilagut G, Valderas JM, Ferrer M, Garin O, López-García E, Alonso J. Interpretación de los cuestionarios de salud SF-36 y SF-12 en España: Componentes físico y mental. Med Clin (Barc). 2008 5 24;130(19):726–35. 18570798 10.1157/13121076

[27] Rejas J, Ribera MV, Ruiz M, Masrramón X. Psychometric properties of the MOS (Medical Outcomes Study) Sleep Scale in patients with neuropathic pain. Eur J Pain. 2007 4;11(3):329–40. 10.1016/j.ejpain.2006.05.002 16765622

[28] Guy W. ECDEU assessment manual for psychopharmacology. Rockville, MD: US Department of Heath,Education, and Welfare Public Health Service Alcohol, Drug Abuse, and Mental Health Administration 1976.

[29] James KE, Block DA, Lee KK, Kraemer HC, Fuller RK. An index for assessing blindness in a multi-centre clinical trial: Disulfiram for alcohol cessation—A VA cooperative study. Stat Med. 1996 7 15;15(13):1421–34. 10.1002/(SICI)1097-0258(19960715)15:13<1421::AID-SIM266>3.0.CO;2-H 8841652

[30] Bang H, Ni L, Davis CE. Assessment of blinding in clinical trials. Control Clin Trials. 2004 4;25(2):143–56. 10.1016/j.cct.2003.10.016 15020033

[31] Thyregod HG, Rowbotham MC, Peters M, Possehn J, Berro M, Petersen KL. Natural history of pain following herpes zoster. Pain. 2007 3;128(1–2):148–56. 10.1016/j.pain.2006.09.021 17070998 PMC1905461

[32] Kukkar A, Bali A, Singh N, Jaggi AS. Implications and mechanism of action of gabapentin in neuropathic pain. Arch Pharm Res. 2013 3 24;36(3):237–51. 10.1007/s12272-013-0057-y 23435945

[33] Finnerup NB, Attal N, Haroutounian S, McNicol E, Baron R, Dworkin RH, et al Pharmacotherapy for neuropathic pain in adults: A systematic review and meta-analysis. Lancet Neurol. 2015 2;14(2):162–73. 10.1016/S1474-4422(14)70251-0 25575710 PMC4493167

[34] Wiffen PJ, Derry S, Bell RF, Rice AS, Tölle TR, Phillips T, et al Gabapentin for chronic neuropathic pain in adults. Cochrane Database Syst Rev. 2017 6 9;(6):CD007938. 28597471 10.1002/14651858.CD007938.pub4 PMC6452908

[35] Johnson P, Becker L, Halpern R, Sweeney M. Real-world treatment of post-herpetic neuralgia with gabapentin or pregabalin. Clin Drug Investig. 2013 1 23;33(1):35–44. 10.1007/s40261-012-0030-4 23179473 PMC3586179

[36] Lee EG, Lee HJ, Hyun DJ, Min K, Kim DH, Yoon MS. Efficacy of low dose gabapentin in acute herpes zoster for preventing postherpetic neuralgia: a prospective controlled study. Dermatol Ther. 2016 5;29(3):184–90. 10.1111/dth.12331 26799145

[37] Lapolla W, DiGiorgio C, Haitz K, Magel G, Mendoza N, Grady J, et al Incidence of postherpetic neuralgia after combination treatment with gabapentin and valacyclovir in patients with acute herpes zoster: Open-label study. Arch Dermatol. 2011 8 1;147(8):901–7. 10.1001/archdermatol.2011.81 21482862

[38] Dworkin RH, Barbano RL, Tyring SK, Betts RF, McDermott MP, Pennella-Vaughan J, et al A randomized, placebo-controlled trial of oxycodone and of gabapentin for acute pain in herpes zoster. Pain. 2009 4;142(3):209–17. 10.1016/j.pain.2008.12.022 19195785

[39] Berry JD, Petersen KL. A single dose of gabapentin reduces acute pain and allodynia in patients with herpes zoster. Neurology. 2005;65(3):444–7. 10.1212/01.wnl.0000168259.94991.8a 16087911

[40] Singh S, Madhwar A, Singh JP. Comparative study of gabapentin in combination with valacyclovir and valacyclovir alone in herpes zoster. Int J Sci fic Study. 2016;3(11):37–40.

[41] Kanodia S, Seth A, Dixit A. Dose related efficacy of gabapentin in acute herpetic neuralgia among geriatric patients. Indian J Dermatol. 2012 9;57(5):362 10.4103/0019-5154.100476 23112355 PMC3482798

[42] Goodman CW, Brett AS. Gabapentin and Pregabalin for Pain—Is Increased Prescribing a Cause for Concern? N Engl J Med. 2017 8 3;377(5):411–4. 10.1056/NEJMp1704633 28767350

[43] Centers for Disease Control and Prevention. Shingles | For Health Care Professionals | Herpes Zoster [Internet]. 2018. Available from: https://www.cdc.gov/shingles/hcp/index.html

[44] Dooling KL, Guo A, Patel M, Lee GM, Moore K, Belongia EA, et al Recommendations of the Advisory Committee on immunization practices for use of herpes zoster vaccines. MMWR Morb Mortal Wkly Rep. 2018 1 26;67(3):103–8. 10.15585/mmwr.mm6703a5 29370152 PMC5812314

[45] Carroll S, Gater A, Abetz-Webb L, Smith F, Demuth D, Mannan A. Challenges in quantifying the patient-reported burden of herpes zoster and post-herpetic neuralgia in the UK: Learnings from the Zoster Quality of Life (ZQOL) study. BMC Res Notes. 2013 11 25;6(1):486. 24274819 10.1186/1756-0500-6-486 PMC4222087

