# Supplementary material for: Efficacy of gabapentin for the prevention of postherpetic neuralgia in patients with acute herpes zoster: A double blind, randomized controlled trial
Source: PLoS One. 2019 Jun 5;14(6):e0217335. doi: 10.1371/journal.pone.0217335 (PMC6550400; doi:10.1371/journal.pone.0217335)
Supplement: S2 Appendix — (DOC) [file pone.0217335.s002.doc]

#

**PROTOCOLO DE ESTUDIO**

**Ensayo clínico, aleatorizado, doble-ciego para evaluar la eficacia y seguridad de la gabapentina frente a placebo en la prevención de la neuralgia postherpética**

**PI12_01813**

# Versión 10/02/2014

# 1. RESUMEN

**1.1. Tipo de solicitud**

Ensayo clínico farmacológico, Fase IV-III

**1.2. Identificador del promotor**

Gerencia de Atención Primaria Mallorca

C/ Reina Esclaramunda nº 9

07003 Palma de Mallorca

Teléfono 971175897

**1.3. Título del ensayo clínico**

“Ensayo clínico, aleatorizado, doble-ciego para evaluar la eficacia y seguridad de la gabapentina frente a placebo en la prevención de la neuralgia postherpética"

**1.4. Investigador Principal: dirección del centro de trabajo**

Manuel Rullán García

[mrullan@ibsalut.caib.es](mailto:mrullan@ibsalut.caib.es)

CS Pollença

C/ Bisbe Desbach, s/n

07460 Pollença

Teléfono 971 738227

**1.5. Centros en los que se prevé realizar el ensayo**

Mallorca:

1. CS Pollença
2. CS Calvià
3. CS Camp Redó
4. CS Inca
5. CS Lluchmajor
6. CS Manacor
7. CS Platja Palma
8. CS Son Gotleu
9. CS Son Pisà
10. CS Son Serra-La Vileta
11. CS Arquitecte Bennasar
12. CS Soller
13. CS Coll d’en Rabassa
14. CS Rafal
15. CS Portocristo
16. CS Cala D’or
17. CS Escola Graduada
18. CS trencadors

**1.6. Nombre y cualificación de la persona responsable de la monitorización**

Por determinar.

**1.7. Intervención**

El tratamiento con gabapentina se irá aumentando hasta la dosis óptima (dosis máxima tolerada, en este sentido, la pauta de escalada de dosis se realizará según se indica en la ficha técnica del producto, FT). El tratamiento puede iniciarse mediante el escalonado de dosis descrito en la Tabla 1 ó administrando 300 mg tres veces al día (TID) en el Día 1. Posteriormente, en base a la respuesta individual del paciente y la tolerabilidad, la dosis puede aumentarse en incrementos de 300 mg/día cada 2-3 días hasta una dosis máxima de 1800 mg/día.

Esquema de dosis: Tabla 1:

TITULACIÓN INICIAL*:

| Dosis | Día1 | Día2 | Día3 | Día4 | Día5 | Día6 | Día7 |
| --- | --- | --- | --- | --- | --- | --- | --- |
| Mañana |  |  | 300mg | 300mg | 300mg | 600mg | Dosis optima |
| Tarde |  | 300mg | 300mg | 300mg | 600mg | 600mg | Dosis optima |
| Noche | 300mg | 300mg | 300mg | 600mg | 600mg | 600mg | Dosis optima |

*Basándose en la tolerabilidad individual del paciente, se intentará alcanzar la dosis máxima 1800mg/día, en caso contrario cada paciente alcanzará su dosis óptima.

El tratamiento se mantendrá durante 4 semanas desde el inicio del cuadro y posteriormente se reducirá la dosis a lo largo de una semana para evitar posibles cuadros de “efecto de retirada” descrito con gabapentina (ansiedad, insomnio, náuseas, dolor, sudoración).

Esquema de retirada de dosis. **Tabla 2**:

RETIRADA*:

| Dosis | Día30 | Día31-32 | Día33 | Día34 | Día35 | Día36 | Día37 |
| --- | --- | --- | --- | --- | --- | --- | --- |
| Mañana | 600mg | 300mg | 300mg | 300mg |  |  | 0mg |
| Tarde | 600mg | 600mg | 300mg | 300mg | 300mg |  |  |
| Noche | 600mg | 600mg | 600mg | 300mg | 300mg | 300mg |  |

*Ejemplo de retirada basada en la dosis máxima tolerada de 1800mg/día

**1.8. Fase del ensayo clínico**

Fase IV-III.

**1.9. Objetivo principal**

Evaluar la eficacia del tratamiento con gabapentina a dosis óptima en la fase aguda del Herpes Zoster, añadida al tratamiento habitual comparado con el tratamiento habitual del Herpes Zoster + placebo de gabapentina en el aumento del porcentaje de pacientes sin NPH a las 12 semanas en pacientes > 50 años con dolor moderado-intenso.

**1.10. Diseño**

Ensayo clínico, multicéntrico, doble-ciego, aleatorizado paralelo de dos ramas de tratamiento.

**1.11. Variable principal de valoración**

La variable principal de eficacia será el porcentaje de pacientes sin NPH al final de seguimiento a las 12 semanas (definida como puntuación 0 en la Escala Visual Analógica (EVA) de dolor.

**1.13. Población en estudio y número total de pacientes**

Pacientes ambulatorios mayores de 50 años diagnosticados de herpes zoster y con dolor moderado-severo. Se incluirán 134 pacientes.

**1.14. Duración del tratamiento**

El tratamiento tendrá una duración de 5 semanas.

**1.15. Calendario previsto**

La duración global del estudio será de 3 años.

# 2. INFORMACION GENERAL

## 2.1. Identificación del ensayo

**Título**

“Ensayo clínico, aleatorizado, doble-ciego para evaluar la eficacia y seguridad de la gabapentina frente a placebo en la prevención de la neuralgia postherpética"

## 2.2. Tipo de ensayo clínico

Ensayo clínico, multicéntrico, doble-ciego, aleatorizado paralelo de dos ramas de tratamiento, ensayo clínico de fase IV-III.

## 2.3. Descripción de la intervención

Tratamiento con gabapentina a dosis óptimas o placebo.

## 2.4. Datos relativos al promotor

Gerencia de atención primaria Mallorca

C/ Reina Esclaramunda nº 9

07003 Palma de Mallorca

Teléfono 971175897

## 2.5. Identificación del monitor

Por determinar

## 2.6. Datos de los investigadores del ensayo

**Investigador Principal**

Manuel Rullán García

[mrullan@ibsalut.caib.es](mailto:mrullan@ibsalut.caib.es)

CS Pollença

C/ Bisbe Desbach, s/n

07460 Pollença

Teléfono 971 738227

**Investigadores Colaboradores y Centros en los que se realizará el ensayo**

Martí Cladera Cifre

CS Pollença

Patricia Lorente Montalvo

CS Calvià

Catalina Comas Pol

CS Camp Redó

Maria Antònia Mir Pons

CS Inca

Tomeu Aulet

Biel Lliteras

CS Lluchmajor

Maria José González Bals

CS Manacor

Apol·lònia Cifre Socias

CS Platja Palma

Francisca Bestard

CS Son Gotleu

Salvador Gestoso Gayá

CS Son Pisà

Mª Antonia Roca

CS Son Serra-La Vileta

Antoni Jover Palmer

CS Arquitecte Bennasar

Mª Antonia Bosch

CS Coll dén Rabassa

Guillen Más

CS Soller

Francisca Comas

CS Rafal

Diana Garau

CS Portocristo

Luís López

CS Cala D’or

Teresa Rosselló

CS Escola Graduada

Biel Moragues

CS Trencadors

## 2.7. Duración prevista del ensayo

La duración prevista del ensayo es de 3 años.

# 3. JUSTIFICACION Y OBJETIVOS

## 3.1. Introducción

El Herpes Zoster (HZ) es una enfermedad autolimitada, caracterizada por una erupción dérmica vesicular distribuida en la región de un dermatoma cutáneo, generalmente unilateral, asociado o no a dolor neuropático. El cuadro agudo se produce por la reactivación del virus varicela-zoster, que ha permanecido latente en un ganglio dorsal desde la primoinfección infantil que cursó clínicamente como una varicela. En esta reactivación, el virus se extiende central y periféricamente desde el ganglio dorsal, produciendo una intensa inflamación de la piel, los nervios periféricos, la raíz nerviosa y ocasionalmente la médula espinal. (1)

La neuralgia postherpética (NPH) se considera su complicación más frecuente, aunque no hay consenso sobre su definición exacta: para algunos autores se trata del dolor que persiste tras la curación de la erupción cutánea aguda, mientras que otros la definen como el dolor que aparece y dura un periodo determinado - al menos un mes tras la aparición de las vesículas o un mínimo de 3-4 meses - tras el inicio de la erupción. A pesar de estas definiciones dispares, modelos de resolución del dolor sugieren que la definición más adecuada es la persistencia del dolor 90 días tras la aparición del HZ. (2-4)

Esta controversia dificulta la estimación de su incidencia, que puede variar entre el 9 y el 34% de los pacientes con HZ, en función de la definición y la población estudiada, así como la interpretación de los resultados de los ensayos clínicos (EECC). (5)

En España la incidencia de HZ es de 4,1 casos cada 1.000 habitantes por año para todas las edades (6). Un 9-10% de los pacientes que padecen un HZ tendrán una NPH; el 1,3% entre los menores de 50 años, el 36% de los mayores de 60 años y el 50% de los mayores de 70 (7). Se estima que el coste anual atribuible en el Reino unido a la infección aguda por HZ y NPH asciende a 108 millones de € (8) y en España en Atención primaria se ha estimado que el coste de la infección HZ es de 378€ y de la NPH 821€ por paciente. (9)

En la etiopatogenia de la NPH parece que estarían involucrados mecanismos centrales y periféricos. Puede haber una lesión de la médula espinal, principalmente de la sustancia gris posterior y anterior, que explicaría los trastornos motores que en ocasiones se asocian a la enfermedad. Se piensa que en la NPH habría una hiperexcitabilidad central provocada por los nociceptores durante la fase aguda, acompañada de una lesión axonal. (10)

A pesar de los avances realizados en su tratamiento y prevención, se trata de un dolor de difícil control, por lo que la NPH continúa siendo un problema clínico significativo. En la actualidad se dispone de diversas estrategias farmacológicas para el tratamiento de la NPH instaurada, pero su eficacia es limitada y se asocia a la aparición de efectos adversos. Esto ha llevado a la búsqueda de alternativas terapéuticas aplicables durante la fase aguda de la infección que permitan prevenir su aparición.

Los resultados de diversos metaanálisis y EECC indican que el uso de antivirales sistémicos durante las primeras 72 h tras la aparición de la erupción cutánea aceleran la curación y disminuyen la duración del dolor, también acortan la duración de la NPH, pero su efecto sobre su incidencia no está bien establecido. (3,10) En esta misma línea, el uso de corticoides sistémicos en la fase aguda también se ha propuesto como una alternativa para prevenir la NPH, pero únicamente ha demostrado su eficacia en el control del dolor y la calidad de vida, no se ha asociado a una reducción de la incidencia de NPH, y su uso es controvertido. (3,5)

Actualmente la eficacia y la seguridad de la nueva vacuna de la varicela zoster, se ha evaluado en el Shingles Prevention Study, un estudio aleatorizado, multicéntrico, doble ciego y controlado con placebo, en el que se incluyó a más de 38.000 individuos inmunocompetentes de 60 o más años, sin antecedentes de HZ, con un seguimiento medio de más de 3 años. El uso de la vacuna se asoció a una reducción moderada de la carga de la enfermedad debida al

HZ (61,1%; p < 0,001), así como de la incidencia de la NPH (66,5%; p < 0,001). Si el paciente había desarrollado el HZ la reducción de NPH era del 12,46% versus 8,56%, una reducción del 31 %. Actualmente la vacuna esta aprobada para la prevención del HZ y la NPH en adultos de 60 o más años y más recientemente, con datos sólo de inmunogenicidad, también se ha aprobado para los adultos de 50-59 años. Sin embargo, se desconoce si su protección perdura más allá de los 4 años y si es necesaria una revacunación y que actualmente su uso no es generalizado. (5,12)

Por otro lado, dado que el dolor intenso durante la fase aguda del HZ es un factor de riesgo conocido para el desarrollo posterior de NPH, otra estrategia terapéutica factible seria la basada en la reducción de la incidencia de la NPH una vez aparecido el HZ mediante un control óptimo del dolor en esta fase.

Sin embargo, prácticamente no se han publicado EECC aleatorizados y controlados con placebo que evalúen la eficacia del tratamiento analgésico en la fase aguda del HZ y su efecto en la incidencia de la NPH. Entre los fármacos estudiados durante la fase aguda del HZ se encuentran la amitriptilina y la gabapentina.

La eficacia de la amitriptilina y otros antidepresivos está bien establecida para tratar el dolor de la NPH, pero la eficacia de su uso en la prevención de NPH se ha evaluado en un único estudio aleatorizado y controlado con placebo en pocos pacientes (n=72). Aunque los resultados fueron favorables -redujo la incidencia de NPH en un 50% -, la existencia en el estudio de importantes defectos metodológicos dificulta la interpretación de los resultados. (12)

Por otro lado, en un estudio exploratorio publicado en 2005 para evaluar la eficacia de gabapentina administrada en dosis única (900 mg) frente a placebo, se mostró una reducción de la intensidad del dolor en el 66% de los pacientes, en comparación al 33% del grupo control, y una disminución de gravedad y superficie afectada por alodinia. Los autores indicaban que el mejor control del dolor asociado al uso de gabapentina durante la fase aguda podría asociarse a una reducción de la incidencia de la NPH, pero no se han realizado nuevos estudios sobre esta cuestión (13).

La gabapentina actúa a nivel de la subunidad α2δ de los canales dependientes de calcio y reducen la liberación de neurotransmisores que en modelos animales han demostrado ser el mecanismo mediador de su efecto analgésico (14), su mecanismo de acción se basaría en la atenuación de la sensibilización central o por limitación de otros cambios funcionales y estructurales centrales que aparecen tras intensos inputs nociceptores como sería, el daño sobre la vía inhibitoria gabérgica.

Recientemente se ha publicado un estudio experimental sin grupo control, es un EECC abierto con un único brazo de tratamiento para evaluar la eficacia de tratamiento con gabapentina (dosis inicial de 300mg/día hasta un máximo de 1800 mg/d) y valaciclovir (1 g/8h durante 7 días) para la prevención de NPH en pacientes con HZ agudo. El estudio incluyó 133 pacientes > 50 años (edad media 64.6 años) inmunocompetentes con un tiempo > 72 horas desde la aparición de los primeros síntomas y dolor moderado (puntuación en la escala EVA >4). La incidencia de NPH en las semanas 12, 16 y 24 fueron del 20.3%, 18.0%, y 9,8% respectivamente. (15)

Aunque se dispone de diversas estrategias farmacológicas para el tratamiento de la NPH una vez instaurada, su eficacia limitada y la aparición frecuente de efectos adversos hacen necesaria la búsqueda de abordajes terapéuticos durante la fase aguda de la infección que permitan prevenir su aparición.

Dado que el dolor intenso durante la fase aguda del HZ es un factor de riesgo para el desarrollo posterior de NPH, cabría esperar que un control óptimo del dolor en esta fase se asociara con una incidencia menor de NPH. La gabapentina es un neuromodulador cuyo efecto analgésico en el tratamiento del dolor en el HZ agudo y en la NPH está bien establecido. (16,17)

Además, se trata de un fármaco con amplia experiencia de uso, se conoce su balance beneficio/riesgo y con buena tolerancia; aunque puede provocar diversos efectos secundarios, como somnolencia, ataxia, diplopía, vértigo y nistagmo, son muy poco frecuentes, leves y revierten con facilidad disminuyendo la dosis o realizando una introducción progresiva; además, su interacción con otros fármacos es mínima, lo que facilita su manejo. Es un fármaco que no se metaboliza en el hígado, que se elimina íntegramente por vía renal y apenas se une a las proteínas plasmáticas (menos del 3%) por tanto presenta un buen perfil de seguridad. La penetración de la barrera hematoencefálica es bastante buena, lo que facilita su acción analgésica. (18)

Uno de los puntos clave para el tratamiento del HZ es el inicio dentro de las primeras 72 horas tras la aparición de los síntomas. En este sentido creemos que la captación de pacientes en fase aguda, con la presentación de las primeras vesículas eruptivas, es mucho más evidente en las consultas de atención primaria y permitirá administrar de manera precoz el tratamiento antiviral y gabapentina.

En nuestra comunidad autónoma la incidencia de nuevos casos de HZ diagnosticados en AP fue de 2200 casos en el año 2010, de los cuales 1400 tenían una edad igual o superior a los 50 años.

A pesar de que prácticamente no se han publicado EECC aleatorizados y controlados que evalúen la eficacia del tratamiento analgésico en la fase aguda del HZ, y se desconoce su efecto en la incidencia de la NPH, los resultados favorables del estudio observacional recientemente publicado nos llevan a plantearnos la realización de este EECC.

Postulamos que una pauta de gabapentina administrada precozmente, durante todo el período eruptivo del HZ hasta completar 4 semanas de tratamiento, asociada a la administración de valaciclovir disminuirá la incidencia de NPH (19).

## 3.2. Objetivos e Hipótesis.

**A) Objetivo principal**

Evaluar la eficacia del tratamiento con gabapentina a dosis óptima (definida como dosis máxima tolerada) durante 5 semanas (4 semanas + 1 semana de retirada), en la fase aguda del Herpes Zoster, añadida al tratamiento habitual (tratamiento antiviral con valaciclovir y tratamiento analgésico) comparado con el tratamiento habitual del Herpes Zoster + placebo de gabapentina en el aumento del porcentaje de pacientes sin NPH (definida como puntuación 0 en la Escala Visual Analógica (EVA) de dolor) a las 12 semanas (5 semanas tratamiento+ 7 semanas sin tratamiento) en pacientes > 50 años con dolor moderado-intenso.

Objetivos secundarios

Evaluar la eficacia del tratamiento con gabapentina a dosis óptima durante 5 semanas, en la fase aguda del Herpes Zoster en pacientes > 50 años, con dolor moderado-intenso añadida al tratamiento habitual (tratamiento antiviral con valaciclovir y tratamiento analgésico) comparado con el tratamiento habitual del Herpes Zoster + placebo de gabapentina en términos de:

- Porcentaje de pacientes sin NPH (definida como puntuación 0 en la escala EVA de dolor) a las 6 semanas del inicio del cuadro agudo (5 semanas de tratamiento + 1 semana de lavado)

- Porcentaje de pacientes que mejoran la sintomatología de NPH a las 6 y 12 semanas del inicio del cuadro agudo (definida como una reducción > 50% de la puntuación en la escala EVA de dolor respecto al basal)

-Reducción de la incidencia de dolor neuropatico medido mediante la escala DN-4.

- Mejora en la puntuación de la escala SF-12.

- Evaluar la seguridad del tratamiento con gabapentina a dosis óptima durante 5 semanas añadida al tratamiento habitual del Herpes Zoster (tratamiento antiviral con valaciclovir y tratamiento analgésico) comparado con el tratamiento habitual del Herpes Zoster + placebo de gabapentina) en términos de Acontecimientos Adversos (AA).

**B) Hipótesis principal**

El tratamiento con gabapentina a dosis óptima (definida como dosis máxima tolerada) durante 5 semanas (4 semanas + 1 semana de retirada), en la fase aguda del Herpes Zoster, añadida al tratamiento habitual (tratamiento antiviral con valaciclovir y tratamiento analgésico) comparado con tratamiento habitual + placebo de gabapentina reducirá en al menos un 25% la incidencia de NPH a las 12 semanas (5 semanas tratamiento+ 7 semanas sin tratamiento) en pacientes > 50 años con dolor moderado-intenso

# 4. TIPO DE ESTUDIO Y DISEÑO

## 4.1. Diseño y tipo de control

Ensayo clínico, multicéntrico, doble-ciego, aleatorizado paralelo de dos ramas de tratamiento. El grupo control recibirá tratamiento con placebo de gabapentina a dosis óptimas. El registro del ensayo se realizará en el "International Standard Randomised Controlled Trial” http://www.controlled-trials.com/.

## 4.2. Proceso de aleatorización

Se generará una secuencia de números aleatorios mediante el programa Epidat 3.1 a cada uno de los dos brazos del ensayo. Los investigadores reclutadores que hayan decidido participar en el estudio, una vez comprobado que los pacientes cumplen los criterios de inclusión y ninguno de los de exclusión, así como hayan firmado el consentimiento informado (CI), realizarán una llamada telefónica a la unidad de investigación de la Gerencia de Atención Primaria para conocer el código de asignación.

La asignación se realizará previa identificación del paciente mediante un algoritmo identificativo se asignará a grupo control o intervención mediante una aleatorización por bloques de 6. A lo largo de la aleatorización se mantendrá un registro del proceso en que se incluya la fecha de solicitud de código de aleatorización, código de identificación del paciente, y brazo de tratamiento asignado.

**4.3 Enmascaramiento:** Tanto el objetivo principal como secundarios del estudio serán evaluados el equipo investigador que permanecerán ciegos al tratamiento recibido por el paciente, así como también la persona que realizará el análisis estadístico.

Para evaluar la eficacia del enmascaramiento, el investigador y la persona que realizará el análisis estadístico deberán elegir a qué grupo de tratamiento creen pertenece cada paciente según las siguientes categorías de respuestas excluyentes entre sí: vaciclovir y placebo, vaciclovir y gabapentina o no sabe/no contesta y se registrarán los motivos de su elección.

# 5. SELECCIÓN DE LOS SUJETOS

## 5.1. Criterios de inclusión y exclusión

### **A) criterios de inclusión**

Para ser incluidos en el estudio los participantes deberán cumplir todos y cada uno de los criterios siguientes de inclusión:

- Pacientes mayores de 50 años.

- Pacientes con diagnóstico de HZ no complicado de menos de 72h de evolución, con dolor moderado a intenso (puntuación del dolor en la escala EVA ≥ 4).

### **B) criterios de exclusión.**

- Pacientes en tratamiento habitual con gabapentina, pregabalina, o antidepresivos triciclitos

- Pacientes con diagnóstico de insuficiencia hepática grave, hipersensibilidad a la sustancia activa o a alguno de los excipientes, o diagnostico de insuficiencia renal moderada o grave

- Pacientes con evidencia de diseminación cutánea o visceral del HZ (más de 20 lesiones fuera del dermatoma adyacente) o afectación oftálmica

- Pacientes con tratamiento inmunosupresor o inmunomodulador (interferón) en las últimas 4 semanas.

- Diagnóstico de inmunodeficiencia en los últimos 3 meses,

-vacunación herpes zoster.

Medicación prohibida: durante el periodo del ensayo no se permitirá iniciar tratamiento con antidepresivos triciclicos o corticoides sistémicos.

## 5.2. Tamaño muestral

Se ha calculado el tamaño muestral en función de la variable principal de eficacia. El porcentaje de incidencia de NPH en los pacientes del grupo vaciclovir+placebo se espera que sea al menos del 45% (Helgason S, Petursson G, Gudmundsson S, Sigurdsson JA. Prevalence of postherpetic neuralgia after a first episode of herpes zoster: prospective study with long term follow up. BMJ. 2000 Sep 30;321(7264):794-6.) y en el grupo vaciclovir+ gabapentina del 20.3% (Lapolla W, Digiorgio C, Haitz K, Magel G, Mendoza N, et alI. incidence of postherpetic neuralgia after combination treatment with gabapentin and valacyclovir in patients with acute herpes zoster: open-label study. Arch Dermatol 2011;147(8):901-7), Hemos calculado una tasa de pérdidas de seguimiento del 20%; un riesgo alfa del 5% y un riesgo beta del 20%. Por tanto, se precisa reclutar a 134 pacientes (67 sujetos en cada brazo de tratamiento).

**5.3. Reclutamiento**

El periodo de reclutamiento tendrá lugar entre junio 2013 y junio de 2015,

Se prevén dos situaciones,

-Pacientes que acudan a su centro de salud en horario de visitas: previamente se realizaran sesiones a todos los médicos del centro en los centros participantes explicando la naturaleza del estudio, en cada uno de estos centros habrá una persona responsable de la inclusión de los casos, los pacientes que potencialmente puedan ser incluidos serán derivados al investigador del centro responsable, quien examinará y en caso de cumplir los criterios del estudio, ofrecerá su participación en el estudio y se instaurará el tratamiento con Valaciclovir y Gabapentina..

-Pacientes que acudan a su centro de salud en horario de urgencias, en este caso será necesario informar a los médicos que realicen este servicio y disponer de Valaciclovir para iniciar el tratamiento que será suministrado por el médico de urgencias, los pacientes que potencialmente puedan ser incluidos será derivados al investigador responsable del centro responsable y se instaurará el tratamiento con Valaciclovir, se le entregará al paciente medicación suficiente hasta que el paciente pueda acudir a su centro de salud, cuando el paciente acuda a la visita con el médico responsable examinará al paciente y en caso de cumplir los criterios del estudio, ofrecerá su participación en el estudio y se instaurará el tratamiento con Gabapentina y se continuará con el tratamiento con Valaciclovir..

.

**Entrenamiento:** Todos los médicos colaboradores en investigación que participen en el estudio recibirán formación básica sobre el proyecto y cumplimentación de cuestionarios.

La formación será impartida por personas del equipo investigador con experiencia en el desarrollo de Ensayos clínicos. Se le entregará el manual del médico colaborador con la explicación detallada del procedimiento y la metodología a seguir.

## 5.4. Criterios de retirada de los pacientes del ensayo o del análisis.

**Importante:** Si el paciente por algún motivo decidiera abandonar la intervención, los pacientes deberán proseguir con el tratamiento más adecuado a juicio del personal sanitario que le atiende, el investigador intentará realizar **la visita final del paciente**, si este accede (Pag. 8-10 del CRD).

En ningún caso estos pacientes podrán incluirse en ningún otro ensayo de investigación. No se les aplicará en ningún momento la intervención.

Sólo se considerará que los pacientes discontinúan el ensayo si hay una pérdida del seguimiento después de agotar todos los medios de contacto o bien si el paciente expresa explícitamente a intención de discontinuar el ensayo y no acudir más a las visitas programadas.

Los eventos posteriores deben ser comunicados a lo largo de todo el ensayo.

Se deberá realizar un verdadero esfuerzo para determinar la o las razones por las que el paciente deja de acudir a las visitas concertadas o es discontinuado del ensayo. Esta información deberá registrarse en el apartado correspondiente del cuaderno de recogida de datos (CRD) y en la hoja de finalización. Si un paciente abandona el estudio deberán intentarse todos los medios para recoger la información relacionada con los endpoints principales.

5.5. Control de Calidad del desarrollo del estudio:

Se realizará una monitorización de los procedimientos. Cada centro se visitará antes de que el centro reclute ningún paciente. Juntamente con el investigador se revisará el protocolo, el manual del investigador, los CRDs, los trámites para obtener el consentimiento informado. Se realizarán dos visitas de monitorización a cada centro a efectos de contrastar los datos registrados en los CRDs con los datos de los documentos originales e historia clínica informatizada.

En la HC informatizada deberá constar que se ha realizado una visita del proyecto PI12_01813 “Ensayo clínico, aleatorizado, doble-ciego para evaluar la eficacia y seguridad de la gabapentina frente a placebo en la prevención de la neuralgia postherpética” y se anotará, la dosis que el paciente esta tomando, puntuación escala EVA basal y final, fechas de las visitas, retirada del estudio si ocurriera, y fecha de finalización del estudio. Esta información será contrastada por el monitor externo del estudio como control de calidad.

Se realizará un estudio piloto con 6 investigadores que incluirán dos pacientes cada uno para conocer el grado de aceptación del protocolo por parte de los profesionales sanitarios y de los pacientes.

## 5.6. Período de reclutamiento

El período de reclutamiento será de 18 meses.

**6. DESCRIPCION DE LA INTERVENCIÓN**

El tratamiento del Herpes Zoster (HZ) agudo incluye por un lado el tratamiento de lesiones dermatológicas, el tratamiento antiviral y el control del dolor.

La intervención que recibirán los pacientes que participen en el ensayo clínico incluirá el abordaje de estos aspectos según las recomendaciones actuales de tratamiento. (Guía Fisterra de Herpes zoster y neuralgia post herpética actualizada el 03/05/2011. Disponible en: http://www.fisterra.com/guias-clinicas/herpes-zosterneuralgia-post-herpetica/ [Consultada el 22 de febrero de 2012]. Fashner J, Bell AL. Herpes Zoster and

Postherpetic Neuralgia: Prevention and Management. Am Fam Physician. 2011; 83:1432-1437**).**

Tratamiento de las lesiones dermatológicas: El objetivo principal es impedir sobreinfección bacteriana y el dolor por roce. Se realizará una higiene adecuada de la piel para evitar una infección bacteriana secundaria, se recomienda usar ropa holgada para reducir el roce y el dolor asociado y no se recomienda el uso de antivirales tópicos.

Tratamiento antiviral: Los resultados de diversos metanálisis y ensayos clínicos indican que el uso de antivirales sistémicos durante las primeras 72 h tras la aparición de la erupción cutánea aceleran la curación y disminuyen la duración del dolor, también acortan la duración de la NPH, aunque su eficacia en la reducción de la incidencia de NPH no está establecida. Se recomienda su uso si se inicia en las primeras 72 horas tras la aparición de la clínica.

En nuestro ensayo este es un criterio de inclusión por lo que todos los pacientes recibirán tratamiento antiviral.

A pesar de que cualquiera de los antivirales autorizados para el tratamiento del HZ estaría indicados, en el ensayo se administrara Valaciclovir 1g/8 h durante 7 días en ambos grupos.

El valaciclovir disminuye la duración del dolor neuropático más rápidamente que el aciclovir (media de días de dolor: Valaciclovir 38-48 días vs. 51 días aciclovir). Además, los resultados de un ensayo clínico aleatorizado que comparó la eficacia de valaciclovir con aciclovir sugieren que el valaciclovir puede ser levemente más eficaz en la resolución del dolor y en la prevalencia de la NPH, en el grupo aciclovir era del 25,7%, frente al 18,6% en el valaciclovir a los 6 meses. En este sentido la elección de valaciclovir como antiviral en el ensayo, obligará a realizar la evaluación del efecto de Gabapentina en las condiciones más desfavorables.

Tratamiento analgésico: Los AINES por vía oral tienen una eficacia modesta a la hora de reducir el dolor neurálgico agudo en el episodio agudo de Herpes Zoster.

En ambos grupos se seguirá la siguiente “escalera analgésica” propuesta por la OMS

Primer Paso: paracetamol. Los AINES parece que tienen una eficacia modesta en el dolor neurálgico agudo del Herpes Zoster. Segundo Paso: combinar al paracetamol un analgésico opioide débil (p.e: codeína); Tercer Paso: combinar al paracetamol un analgésico opioide potente (p.e: morfina).

Tratamiento con Gabapentina o placebo: Los pacientes incluidos en el ensayo recibirán el tratamiento médico habitual y serán aleatorizados a recibir gabapentina o placebo.

El tratamiento con gabapentina se irá aumentando hasta la dosis óptima (dosis máxima tolerada, en este sentido, la pauta de escalada de dosis se realizará según se indica en la ficha técnica del producto, FT).

Hay que tener en cuenta que, como todos los agentes anticonvulsivantes, gabapentina actúa sobre el sistema nervioso central y puede producir somnolencia, vértigo, u otros síntomas relacionados. Aunque sólo sean de intensidad leve o moderada, estos efectos adversos pueden ser potencialmente peligrosos para los pacientes que conducen o manejan maquinaria, particularmente hasta que el momento en que la

experiencia individual del paciente esté consolidada.

Debe aconsejarse a los pacientes que se abstengan de conducir o manejar maquinaria hasta que se conozcan los efectos de gabapentina, así mismo la ingesta de alcohol o fármacos puede agravar algunos de los efectos secundarios de gabapentina relacionados con el SNC p. Ej. somnolencia, ataxia.

El tratamiento puede iniciarse mediante el escalonado de dosis descrito en la Tabla 1 ó administrando 300 mg tres veces al día (TID) en el Día 1. Posteriormente, en base a la respuesta individual del paciente y la tolerabilidad, la dosis puede aumentarse en incrementos de 300 mg/día cada 2-3 días hasta una dosis máxima de 1800 mg/día.

Esquema de dosis: **Tabla** 1:

TITULACIÓN INICIAL*:

| Dosis | Día1 | Día2 | Día3 | Día4 | Día5 | Día6 | Día7 |
| --- | --- | --- | --- | --- | --- | --- | --- |
| Mañana |  |  | 300mg | 300mg | 300mg | 600mg | Dosis optima |
| Tarde |  | 300mg | 300mg | 300mg | 600mg | 600mg | Dosis optima |
| Noche | 300mg | 300mg | 300mg | 600mg | 600mg | 600mg | Dosis optima |

*Basándose en la tolerabilidad individual del paciente, se intentará alcanzar la dosis máxima 1800mg/día, en caso contrario cada paciente alcanzará su dosis óptima.

El tratamiento se mantendrá durante 4 semanas desde el inicio del cuadro y posteriormente se reducirá la dosis a lo largo de una semana para evitar posibles cuadros de “efecto de retirada” descrito con gabapentina (ansiedad, insomnio, náuseas, dolor, sudoración).

Esquema de retirada de dosis: Tabla **2**:

RETIRADA*:

| Dosis | Día30 | Día31-32 | Día33 | Día34 | Día35 | Día36 | Día37 |
| --- | --- | --- | --- | --- | --- | --- | --- |
| Mañana | 600mg | 300mg | 300mg | 300mg |  |  | 0mg |
| Tarde | 600mg | 600mg | 300mg | 300mg | 300mg |  |  |
| Noche | 600mg | 600mg | 600mg | 300mg | 300mg | 300mg |  |

*Ejemplo de retirada basada en la dosis máxima tolerada de 1800mg/días

# 7. DESARROLLO DEL ENSAYO Y EVALUACIÓN DE LA RESPUESTA

## 7.1. Desarrollo del ensayo

- Visita de screening o basal: El investigador del estudio a los pacientes elegibles que cumplan los criterios de inclusión del estudio y no incurran en ninguno de los criterios de exclusión, se les ofrecerá participar en el estudio, se le entregará la hoja de información al paciente sobre el estudio y se le entregará el consentimiento informado.

Todos los pacientes incluidos en el ensayo serán evaluados en una primera entrevista o visita basal y se registrarán en el CRD los siguientes datos:

*Revisión de los criterios de inclusión y exclusión de estudio

*Evaluación basal en la escala EVA.

*Evaluación de la escala SF-12 de calidad de vida.

*Datos sociodemográficos (edad, sexo y origen del paciente)

*Enfermedades concomitantes: cualquier enfermedad que esté presente al inicio del ensayo.

*Registro de la medicación concomitante cualquier medicación que el paciente tome durante el ensayo incluido el período de selección. Cualquier cambio en la medicación concomitante debe registrarse en cada visita de seguimiento.

*Entrega de la medicación antiviral y de la gabapentina según pauta de dosificación establecida en ficha técnica, y se le recordará que debe traer en la visita final el bote de la medicación.

-Visita 1 de tratamiento. Semana1

El investigador del estudio a los pacientes incluidos entregará la medicación antiviral y de la gabapentina o placebo según pauta de dosificación establecida en ficha técnica e instrucciones para proceder al recuento de la medicación: se informará al paciente de que debe registrar diariamente la toma de la medicación.

Registrará las nuevas enfermedades concomitantes que hayan aparecido y la medicación nuevamente recetada.

Registrará los acontecimientos adversos información que deberá incluir: descripción del acontecimiento adverso, fecha de inicio, fecha de finalización o si continua y posible causalidad con el fármaco de estudio

-Visita 2 de tratamiento. Semana 4

El investigador del estudio en esta visita con el paciente pautará la retirada de dosis de Gabapentina según ficha técnica e instrucciones para proceder al recuento de la medicación en la fase de retirada: Se informará al paciente de que debe registrar diariamente la toma de la medicación.

Registrará las nuevas enfermedades concomitantes que hayan aparecido y la medicación nuevamente recetada.

Registrará los acontecimientos adversos información que deberá incluir: descripción del acontecimiento adverso, fecha de inicio, fecha de finalización o si continua y posible causalidad con el fármaco de estudio

Visita 3 de seguimiento (Semana 6). El investigador del estudio.

Realizará el recuento de pastillas y registrará la medida de la adherencia al tratamiento.

Evaluará la eficacia del tratamiento a corto plazo mediante evaluación del dolor mediante escala visual analógica.

Evaluación la calidad de vida a las 6 semanas mediante la escala SF-12 de calidad de vida.

Registrará la calidad del sueño mediante escala MOS-Sleep.

Registrará las nuevas enfermedades concomitantes que hayan aparecido y la medicación nuevamente recetada.

Registrarán los acontecimientos adversos información que deberá incluir: descripción del acontecimiento adverso, fecha de inicio, fecha de finalización o si continua y posible causalidad con el fármaco de estudio

Visita final de seguimiento (Semana 12): El investigador del estudio:

Evaluará la eficacia del tratamiento mediante evaluación del dolor mediante escala visual analógica.

Evaluará la calidad de vida final mediante la escala SF-12 de calidad de vida y la calidad del sueño mediante la escalaMos-Sleep y realizará la evaluación del paciente mediante la escala de dolor neuropatico DN-4.

Registrará las nuevas enfermedades concomitantes que hayan aparecido y la medicación nuevamente recetada, especialmente las prescritas para el dolor.

Registrará los acontecimientos adversos información que deberá incluir: descripción del acontecimiento adverso, fecha de inicio, fecha de finalización o si continua y posible causalidad con el fármaco de estudio.

**7.2. Variable dependiente principal.**

Porcentaje de pacientes sin NPH al final de seguimiento a las 12 semanas (definida como puntuación 0 en la Escala Visual Analógica (EVA) de dolor.

Se utilizará la escala EVA con una puntuación de 0 (ningún dolor) a 10 (pero dolor imaginable).

**7.3. Variables dependientes secundarias.**

- Porcentaje de pacientes sin NPH en la visita de seguimiento a las 6 semanas

- Porcentaje de pacientes sin NPH a las 6 semanas de inicio del cuadro agudo definida como puntuación 0 en la escala EVA de dolor.

- Porcentaje de pacientes considerados respondedores que mejoran la sintomatología de NPH a las 6 y las 12 semanas del inicio del cuadro agudo: Definimos respondedores a aquellos pacientes que consiguen una reducción menor o igual al 50% de la puntuación en la escala EVA de dolor respecto al basal.

·Variables de calidad de vida:

-Escala de calidad de vida: En las semanas 6 y 12 se administrará el test SF-12 versión 2 de calidad de vida en su versión española ([Med Care. 1996 Mar;34(3):220-33.](http://www.ncbi.nlm.nih.gov/pubmed?term=Med Care. 1996 Mar%3B34(3)%3A220-33.)), escala que consta de 12 ítems provenientes de las 8 dimensiones del SF-36: función Física, función social, rol físico, rol emocional, salud mental, vitalidad, dolor corporal y salud general (1).

Las opciones de respuesta forman escalas de tipo Likert que evalúan intensidad o frecuencia. El número de opciones de respuesta oscila entre tres y seis, dependiendo del ítem.

-Puntuación en la escala total de sueño MOS-Sleep en su versión española (Eur J Pain. 2007 Apr;11(3):329-40.) en las semanas 6 y 12, y puntuación en la dimensión de perturbaciones del sueño, consta de 6 dimensiones: alteración del sueño (tiempo que tarda en quedarse dormido), medida en que el sueño no es tranquilo, problemas para quedarse dormido, frecuencia en la que se despierta mientras duerme); adecuación del sueño; somnolencia diurna; ronquidos; levantarse falto de aire o con dolor de cabeza, y cantidad de sueño , las preguntas a cada dimensión se puntúan con un rango de 0-100. A mayor puntuación, mayor es la presencia del concepto que se está evaluando, a excepción de la dimensión de adecuación del sueño, donde se considera óptimo si el paciente responde que el número de horas dormidas se encuentra en el rango 7-8 horas.

-Paciente con diagnostico de dolor neuropatico, según la escala versión española del cuestionario DN4 (puntuación en la escala > 3) (Health Qual Life Outcomes. 2007 Dec 4;5:66), escala de dos dimensiones Calidad del dolor y exploración neurológica del dolor, que consta de 4 preguntas y 10 ítems de respuesta sobre las características del dolor.

-Escala de impresión de mejora global del paciente en su versión española ([Int Urogynecol J Pelvic Floor Dysfunct. 2008 Aug;19(8):1109-16](http://www.ncbi.nlm.nih.gov/pubmed?term=18360735)): consiste en una sola pregunta que solicita al paciente que clasifique el alivio obtenido con el tratamiento que sigue según una escala de Likert de siete puntos.

-Consumo de analgésicos: En las semanas 6 y 12 se evaluará el consumo de analgesicos relacionado con la NPH.

Todas las variables de eficacia y calidad de vida se recogerán mediante entrevista con el paciente.

- Variable de seguridad:

Incidencia de acontecimientos adversos (AA) y AA graves. (fuentes: entrevista con el paciente en las visitas de seguimiento del estudio e historia clínica de Atención Primaria).

## 7.4. Variables independientes

La **variable independiente principal** será la pertenencia al grupo de tratamiento con placebo o gabapentina.

**Variables Independientes:**

**-**Variables sociodemográficas: edad, sexo e IMC. (fuente: entrevista y recogida de datos antropométricos en la visita basal):

1. Edad
2. Sexo (hombre/mujer)
3. IMC

-Porcentaje de adherencia al tratamiento prescrito: En la visita final, se invitará al paciente a que traiga el bote con la medicación de Gabapentina y se realizará un recuento de las pastillas que ha tomado y las que han sido prescritas.

-Presencia de Diabetes Mellitus, enfermedades autoinmunitarias y neuropatías.

(fuentes: entrevista e historia clínica)

-Medicación concomitante (analgésicos, antidepresivos triciclicos, opioides, vacunación contra el Herpes, etc). (fuentes: entrevista e historia clínica).

**7.5. Estudio Piloto.**

Se realizará un estudio piloto con 6 investigadores que incluirán dos pacientes cada uno para conocer el grado de aceptación del protocolo por parte de los profesionales sanitarios y de los pacientes, comprobación de la adecuación de los circuitos en la detección de pacientes con herpes zoster, ofrecimiento de participación en el estudio en un periodo no superior a las 72h, comunicación con la unidad de investigación y posterior aleatorización.

# 8. EFECTOS ADVERSOS

Incidencia de acontecimientos adversos (AA) y AA graves. En un estudio a doble ciego en pacientes con dolor neuropático en tratamiento con gabapentina, apareció somnolencia, edema periférico y astenia en un porcentaje algo mayor en pacientes > 65, frente a pacientes más jóvenes. A parte de estos hechos, las investigaciones clínicas en este grupo de edad no indican un perfil de acontecimientos adversos diferente del observado en pacientes jóvenes.

A pesar de esto como no ha habido estudios sistemáticos en pacientes mayores de 65 años y el ensayo prevé la inclusión de pacientes mayores de 50 años, se recogerá de manera sistemática información referente a estos AA. (Gabapentina. Ficha técnica del producto).

En el tratamiento con gabapentina se han detectado **efectos adversos** como:

Trastornos de la sangre y del sistema linfático:

Frecuentes: leucopenia.

Raras: trombocitopenia.

Trastornos del sistema inmunológico:

Raras: reacciones alérgicas (por ejemplo, urticaria).

Trastornos del metabolismo y de la nutrición:

Frecuentes: anorexia, aumento del apetito.

Trastornos psiquiátricos:

Frecuentes: hostilidad, confusión e inestabilidad emocional, depresión, ansiedad, nerviosismo,

pensamiento anormal.

Raras: alucinaciones.

Trastornos del sistema nervioso:

Muy frecuentes: somnolencia, mareos, ataxia.

Frecuentes: convulsiones, hipercinesia, disartria, amnesia, temblor, insomnio, dolor de cabeza,

sensaciones como parestesias, hipoestesia, coordinación anormal, nistagmo y aumento, descenso o ausencia de reflejos.

Raras: trastornos del movimiento (por ejemplo, coreoatetosis, discinesia, distonía).

Trastornos oculares:

Frecuentes: alteraciones visuales tales como ambliopía, diplopía.

Trastornos del oído y del laberinto:

Frecuentes: vértigo.

Raras: tinnitus.

Trastornos cardiacos:

Raras: palpitaciones

Trastornos vasculares:

Frecuentes: hipertensión, vasodilatación.

Trastornos respiratorios, torácicos y mediastínicos:

Frecuentes: disnea, bronquitis, faringitis, tos, rinitis.

Trastornos gastrointestinales:

Frecuentes: vómitos, náuseas, anomalías dentales, gingivitis, diarrea, dolor abdominal, dispepsia,

estreñimiento, sequedad de boca o de garganta, flatulencia.

Raras: pancreatitis.

Trastornos hepatobiliares:

Raras: hepatitis, ictericia.

Trastornos de la piel y del tejido subcutáneo:

Frecuentes: edema facial, púrpura descrita más a menudo como contusiones resultantes de un traumatismo físico, erupción, prurito, acné.

Raras: síndrome de Stevens-Johnson, angioedema, eritema multiforme, alopecia.

Trastornos musculoesqueléticos y del tejido conjuntivo:

Frecuentes: artralgia, mialgia, dolor de espalda, espasmos.

Trastornos renales y urinarios:

Poco frecuentes: incontinencia

Raras: fallo renal agudo.

Trastornos del aparato reproductor y de la mama:

Frecuentes: impotencia.

Trastornos generales y alteraciones en el lugar de administración:

Muy frecuentes: fatiga, fiebre.

Frecuentes: edema periférico o generalizado, marcha anormal, astenia, dolor, malestar, síndrome gripal.

Raras: reacciones por síndrome de retirada (principalmente ansiedad, insomnio, náuseas, dolores, sudoración), dolor de pecho. Se han notificado muertes inesperadas, en las que no se ha establecido una relación causal con el tratamiento con gabapentina.

Exploraciones complementarias:

Frecuentes: disminución del recuento leucocitario, aumento de peso.

Raras: fluctuaciones de la glucemia en pacientes con diabetes, aumento en los valores de los análisis de función hepática.

Lesiones traumáticas e intoxicaciones:

Frecuentes: lesiones accidentales, fractura, abrasión.

En los casos graves, se han descrito pancreatitis aguda y tendencias suicidas durante el tratamiento con gabapentina.

Los acontecimientos adversos se clasifican en:

Leve: El acontecimiento adverso relacionado con el tratamiento con la gabapentina no modifica la vida normal del paciente
Moderado: El acontecimiento adverso relacionado con el tratamiento con la gabapentina altera la vida normal del paciente (supone visita medica o baja laboral)
Grave: El acontecimiento adverso relacionado con el tratamiento con la gabapentina pone en peligro la vida del paciente de forma directa.
Mortal: El paciente es éxitus como consecuencia del tratamiento con la gabapentina.

Se considera acontecimientos adverso grave a cualquier síntoma de retirada Grave o Mortal y cualquier causa de hospitalización o muerte ya sea o no causada por el tratamiento con la gabapentina.

Si se presenta un acontecimiento adverso grave se deberá rellenar la hoja de registro de acontecimientos adversos graves (Anexo15) y enviar por Fax antes de las 48 horas al centro coordinador:971175888:

**9. ASPECTOS ÉTICOS**

Este proyecto ha sido sometido a la aprobación del Comité Ético de las Illes Balears. Los profesionales sanitarios que participan en el estudio firmaran documento por el que se comprometen a garantizar la confidencialidad de los datos de todos los pacientes. Antes de iniciar cualquier procedimiento del estudio deberá obtenerse el consentimiento informado de cada paciente. En la hoja de información al paciente constará la información necesaria y será previamente aprobado por el CEIC de referencia. El consentimiento se guardará con los documentos del estudio del investigador. Para la obtención del consentimiento se seguirán las recomendaciones recogidas en la Declaración de Helsinki y los investigadores firmarán un documento de confidencialidad de los datos recogidos.

# 10. CONSIDERACIONES PRÁCTICAS

**Organización del estudio y responsabilidades**

## 10.1. Comités de Monitorización

**A) Comité Ejecutivo.** Estará formado por el Dr. Manuel Rullán García, Dr. Joan Llobera Cànaves, serán los encargados de supervisar la calidad y rigor metodológico de la recogida de datos y del análisis. También se ocupará de desarrollar un mecanismo de transmisión de la información a los investigadores de cada centro. Evaluará los resultados de la monitorización y será el responsable de corregir los problemas detectados. Revisará y aprobará las presentaciones o publicaciones realizadas.

**B) Comité de Coordinación:** Estará formado el Dr. Manuel Rullán García y Alfonso Leiva Rus. Serán los responsables de que la aplicación del protocolo sea uniforme en los diferentes centros de Salud y de mantener el ritmo de inclusión, organizar el envío y recogida de material, coordinación del personal del estudio.

**C) Comité de seguridad:** Estará formado por el Dr. Manuel Rullán Garcia, Dr. Joan Llobera Cànaves, su función será la de recibir, revisar y notificar si fuera preciso a la Agencia Española del Medicamento, los efectos adversos graves del estudio. También estará dentro de sus funciones la apertura del codigo de aleatorización.

## 10.2. Responsabilidades

Cada investigador que decida participar en el estudio, se responsabilizará del reclutamiento de los pacientes, de la solicitud y consecución del consentimiento informado escrito, de la aplicación de los criterios de inclusión y exclusión, de la notificación de la inclusión al centro coordinador, de la citación de pacientes, de su entrevista y examen periódico de acuerdo al protocolo de estudio, de la recogida y envío periódico de datos al centro coordinador y de la comunicación de efectos adversos graves.

El centro coordinador del estudio será responsable de la planificación del estudio, del diseño y realización de los instrumentos de recogida de datos, del análisis estadístico, de la edición del manual de operaciones y procedimientos, de organizar las sesiones de entrenamiento, estandarización y certificación de investigadores, de organizar el estudio piloto y de su análisis, de la edición posterior del manual y CRD si procede, del envío de cuestionarios y materiales de estudio a los centros participantes, de supervisar y coordinar la recogida de datos (monitorización presencial en una submuestra de pacientes) y del procesamiento de datos de todos los centros.

La coordinación general será a cargo de la Unidad de Investigación de la Gerencia de Atención Primaria de Mallorca

# 11. ANALISIS ESTADÍSTICO

## Manejo global de los datos

A todo sujeto incluido en el estudio se le asignará un identificador numérico único creado con un algoritmo basado en los códigos de la Comunidad de procedencia, el Centro de salud al que pertenece, el médico colaborador y el número de caso.

El identificador estará presente en cada una de las tablas del estudio. A continuación, se enumeran las distintas tablas de datos que se incluirán en el estudio:

1. Tabla de inclusión. Se corresponderá con los datos de los CRDs de las visitas –1 y 0.
2. Tabla de seguimiento. Se corresponden con los datos de los CRDs de las visitas 1,2 y3.
3. Tabla visita final: Se corresponden con los datos de los CRDs de la visita 4.
4. Tabla de monitorización Registro de las monitorizaciones llevadas a cabo a cada uno de los pacientes incluidos.

El programa ***Teleform*** dispone de un módulo de verificación que garantiza la calidad de la entrada de datos. En cualquier caso, un 30% de los cuestionarios se volverán a entrar manualmente y se procederá a la validación de datos mediante la verificación de la concordancia de ficheros con el programa ***Epiinfo*.** Los datos en soporte impreso se almacenarán localmente y periódicamente se enviarán copias al centro coordinador donde serán procesados y almacenados en soporte magnético en el servidor del centro coordinador. Se efectuarán 2 copias de seguridad en cinta magnética de todos los datos entrados cada mes. Se utilizará un aparato servidor IBM 3500 backup 400 Gb.

Todos los análisis estadísticos se realizarán con el programa **SPSS para Windows v. 15**. El nivel de significación estadística se establece en el 5% bilateral. El análisis estadístico será responsabilidad de la unidad de investigación de Gerencia de Atención Primaria de Mallorca.

Para el análisis estadístico se utilizará el programa SPSS para Windows v. 15

- Análisis descriptivo, etiquetado y depuración de los datos: Valoración de los valores atípicos y extremos (`outliers´), detección y etiquetado de los valores perdidos y/o no aplicables, descripción de la distribución de cada una de las variables. Pruebas de normalidad, gráficas de dispersión.

- Análisis comparativo basal: Comparación entre las características sociodemográficas entre el grupo intervención y el grupo control, mediante la prueba de t-test y de chi-cuadrado. En caso de no cumplirse las asunciones de normalidad se aplicarán pruebas no paramétricas.

- Análisis comparativo final: Comparación de las características clínicas, de medicación entre los grupos de placebo y tratamiento, mediante la prueba t-test y de chi-cuadrado. En caso de no cumplirse las asunciones de normalidad se aplicarán pruebas no paramétricas. La relevancia clínica de la intervención se determinará a partir de la ausencia de

dolor a las 12 semanas en los grupos control e intervención y en la relación beneficio/riesgo de la intervención, se calculará la reducción del riesgo relativo (RRR), la reducción del riesgo absoluto (RRA) y el número necesario de pacientes a tratar (NNT). Se realizará un análisis crudo mediante chi-cuadrado y ajustado mediante regresión logística de las características basales que muestren ser diferentes en los grupos control e intervención. Todos los

análisis se realizarán por intención de tratar (`intention to treat´). El nivel se significación estadística se establece al 5% bilateral.

**12. DIFICULTADES Y LIMITACIONES DEL ESTUDIO.**

Al incluir pacientes con dolor moderado o intenso podría ocurrir un efecto de regresión a la media, existiría por tanto una tendencia a que estos pacientes pasaran a valores de dolor menos intenso independientemente del efecto de la gabapentina o el placebo, sin embargo, al estar comparando dos grupos asumimos que dicho efecto ocurrirá en los dos grupos por igual y por tanto no afectará a la estimación de la magnitud del riesgo.

La gabapentina es un medicamento que puede presentar efectos adversos, esto por una parte podría provocar que el grupo de fármaco activo tenga un mayor número de perdidas de seguimiento, si estas perdidas son más frecuentes en los pacientes de mayor edad, con más comorbilidad o mayor inmunosupresión podríamos estar incurriendo en un error de tipo I, para evitarlo se ha optado por un análisis por intención de tratar.

Por otra parte, los pacientes que toman el fármaco activo podrían reconocer que no están tomando placebo por la presencia de dichos efectos adversos, para controlar por la posible falta de cegamiento del paciente y del médico que realiza las visitas de seguimiento, se han limitado el número de visitas de seguimiento y para conocer la magnitud del enmascaramiento del placebo, a paciente y médico, se le preguntará a qué grupo de tratamiento que ellos piensan pertenecen.

**13. CALENDARIO DE TRABAJO**

**Preparación del proyecto: Enero-abril 2013**

- Preparación de los CRDs, hoja de inclusión y redacción pormenorizada del protocolo.
- Revisión de los CRDs por todos los subproyectos y aportación de mejoras.
- Captación de los médicos para el ensayo.
- Registro ISRCTN

**Formación y pilotaje: Abril –Julio 2013:**

- Formación en metodología de investigación en ensayos clínicos para los facultativos incluidos.
- Pilotaje de los cuestionarios y rectificaciones pertinentes (15 de abril)
- Segunda reunión de coordinación para preparar el trabajo de campo, manual del médico colaborador en investigación y actuaciones ante las desviaciones de protocolo.
- Contacto con el Servicio de Farmacia del Hospital Universitario Son Espases, y compra de la medicación del estudio y placebo.
- CEIC
- Registro AEMPS

**Trabajo de campo: agosto 2013-abril 2015:**

- Inclusión de los pacientes y asignación aleatoria.
- Evaluación basal e inicio del seguimiento del paciente
- Recepción de CRDs
- Entrada de datos
- Reunión Equipo investigador
- Control de calidad, resolución queries.
- Monitorización
- Elaboración de informes preliminares
- Memorias anuales FIS 2013/2014

**Final: Mayo –septiembre 2015:**

- Análisis de datos.
- Reunión con informe general de los resultados, discusión de los mismos y planificación de la preparación de artículos.
- Difusión de resultados.
- Memoria
- Final Fis 2015
- Búsqueda bibliográfica y preparación de los manuscritos.
- Memoria final.

Tabla 1: Tabla resumen de las pruebas e intervenciones se realizarán en el estudio:

| Visita Basal: screening | Visita 1:Tratamiento | Visita 2:Tratamiento | Visita 3: Tratamiento | Visita 4: Final |
| --- | --- | --- | --- | --- |
| Semana 0 | Semana 1 | Semana 4 | Semana 6 | Semana 12 |
| Investigador | Investigador | investigador | Investigador | Investigador |
| Criterios de Inclusión: - Pacientes mayores de 50 años.  - Pacientes con diagnóstico de HZ no complicado de menos de 72h de evolución, con dolor moderado a intenso (puntuación del dolor en la escala EVA ≥ 4). | Entrega de la medicación antiviral y de la gabapentina o placebo según pauta de dosificación establecida en ficha técnica. |  |  |  |
| Criterios de exclusión: - Pacientes en tratamiento habitual con gabapentina, pregabalina o antidepresivos triciclitos  - Pacientes con diagnóstico de insuficiencia hepática grave, hipersensibilidad a la sustancia activa o a alguno de los excipientes o diagnóstico de insuficiencia renal moderada o grave.  - Pacientes con evidencia de diseminación cutánea o visceral del HZ (más de 20 lesiones fuera del dermatoma adyacente) o afectación oftálmica  - Pacientes con tratamiento inmunosupresor en los últimos 3 meses o inmunomodulador (interferón) en las últimas 4 semanas, diagnóstico de  inmunodeficiencia de cualquier tipo.  -Vacunación herpes zoster. |  |  |  |  |
| Datos sociodemográficos (edad, sexo y origen del paciente) y antropométricos (peso y talla) |  |  |  |  |
| Enfermedades concomitantes | Registro de nuevas enfermedades concomitantes | Registro de nuevas enfermedades concomitantes | Registro de nuevas enfermedades concomitantes | Registro de nuevas enfermedades concomitantes |
| Registro de la medicación concomitante | Registro de la medicación concomitante | Registro de la medicación concomitante | Registro de la medicación concomitante | Registro de la medicación concomitante |
| Aleatorización  TLF:(971175883 (76704) / 659691565) o Fax: 971175888. | Registro de los acontecimientos adversos | Registro de los acontecimientos adversos | Registro de los acontecimientos adversos | Registro de los acontecimientos adversos |
| Evaluación basal en la escala EVA.  Y de la escala SF-12 de calidad de vida. |  |  | Evaluación basal en la escala EVA, escala MOS-Sleep  Y de la escala SF-12 de calidad de vida. | Evaluación basal en la escala EVA, escala MOS-Sleep, la escala SF-12 de calidad de vida y escala DN4. |
| Entrega bote medicación |  |  | Recordatoria bote medicación en la siguiente visita | Recuento de la medicación |

**14. BIBLIOGRAFIA**

(1) González-Escalada J. R. Pregabalina en el tratamiento del dolor neuropático periférico. Rev. Soc. Esp. Dolor 2005;12: 169-180.

(2) Desmond RA, Weiss HL, Arani RB. Clinical applications for change-point analysis of herpes zoster pain. Journal of Pain and Symptom Management. 2002; 23(6): 510-6.

(3) Whitley RJ, Volpi A, McKendrick M, Van Wijck A, Oaklander AL. Management of herpes zoster and post-herpetic neuralgia now and in the future. Journal of clinical virology 2010;48:S20-S28.

(4) Arani RB, Soong SJ, Weiss HL, Wood MJ, Fiddian RJ et al. Phase specific analysis of herpes zoster associated pain data: a new statistical approch. Stat Med 2001;20:2429-39.

(5) López E, Agustí A. Prevención de la neuralgia postherpética. Med Clin (Barc). 2008;130(20):794-6.

(6) Cebrián-Cuenca AM, Díez-Domingo J, Rodríguez MS, Puig-Barberá J, Navarro-Pérez J; 'Herpes Zoster Research Group of the Valencian Community'. Epidemiology of herpes zoster infection among patients treated in primary care centres in the Valencian community (Spain). BMC Fam Pract. 2010 May 6;11:33.

(7) Helgason S, Petursson G, Gudmundsson S, Sigurdsson JA. Prevalence of postherpetic neuralgia after a first episode of herpes zoster: prospective study with long term follow up. BMJ. 2000 Sep 30;321(7264):794-6.

(8) Wahreham DW, Breuer J. Herpes Zoster. Clinical Review. BMJ 2007;334:1211-5.

(9) Cebrián-Cuenca AM, Díez-Domingo J, San-Martín-Rodríguez M, Puig-barbera J, Navarro-Pérez J et al. Epidemiology and cost of herpes zoster and postherpetic neuralgia among patients treated in primary care centers in valencian community of Spain. BMC Infectious Diseases 2011;11:302

(10) Qifu Li, Ning Chen, Jie Yang, Muke Zhou, Dong Zhou, Quanwei Zhang, Li He. Tratamiento antiviral para la prevención de la neuralgia posherpética (Revision Cochrane traducida). En: Biblioteca Cochrane Plus 2009 Número 3. Oxford: Update Software Ltd. Disponible en: http://www.update-software.com. (Traducida de The Cochrane Library, 2009 Issue 2 Art no. CD006866. Chichester, UK: John Wiley & Sons, Ltd.).

(11) Oxman MN, Levin MJ, Johnson GR, Schmader KE, Straus SE, Gelb LD, et al, and the Shingles Prevention Study Group. A vaccine to prevent herpes zoster and postherpetic neuralgia in older adults. N Engl J Med. 2005;352:2271-84.

(12) Bowsher D. The effects of pre-emptive treatment of post-herpetic neuralgia: a randomised, double-blind, placebocontrolled trial. J Pain Symptom Manage. 1997;13:327-31.

(13) Berry JD, Petersen KL. A single dose of gabapentin reduces acute pain and allodynia in patients with herpes zoster. Neurology. 2005;65:444-7.

(14) Tenser RB, Dworkin RH. Herpes zoster and the prevention of postherpetic neuralgia. Beyond antiviral therapy. Neurology 2005;65:349 -350.

(15) Lapolla W, Digiorgio C, Haitz K, Magel G, Mendoza N, et al.Incidence of postherpetic neuralgia after combination treatment w ith gabapentin and valacyclovir in patients with acute herpes zoster: open-label study. Arch Dermatol 2011;147(8):901-7

(16) Fashner J, Bell AL. Herpes Zoster and Postherpetic Neuralgia: Prevention and Management. Am Fam Physician. 2011;83:1432-1437.

(17) Guía Fisterra de Herpes zoster y neuralgia post herpética actualizada el 03/05/2011. Disponible en: http://www. fisterra.com/guias-clinicas/herpes-zoster-neuralgia-post-herpetica/ [Consultada el 22 de febrero de 2012].

(18) Gabapentina. Ficha técnica del producto. Disponible en AEMPS.

(19) Green CB. Prevent rather than treat postherpetic neuralgia by prescribing gabapentina earlier in patients with herpes zoster. Practice gaps. Arch Dermatool 2011;147:908.

**15. ANEXO 1**

**HOJA DE INFORMACIÓN AL PACIENTE**

**TÍTULO DEL ESTUDIO:** Ensayo clínico, aleatorizado, doble-ciego para evaluar la eficacia y seguridad de la gabapentina frente a placebo en la prevención de la neuralgia postherpetica

**CÓDIGO DEL PROMOTOR**: PI12_01813

**PROMOTOR:** Gerencia de Atención Primaria Mallorca

**INVESTIGADOR PRINCIPAL**: Manuel Rullán García

**CENTRO:** CS Pollença

**INTRODUCCION**

Nos dirigimos a usted para informarle sobre un estudio de investigación en el que se le invita a participar. El estudio ha sido aprobado por el Comité de Ética de la Investigación de las Illes Balears y la Agencia Española del Medicamento y Productos Sanitarios, de acuerdo a la legislación vigente, y se lleva a cabo con respeto a los principios enunciados en la declaración del Helsinki y a las normas de buena práctica clínica.

Nuestra intención es tan solo que usted reciba la información correcta y suficiente para que pueda evaluar y juzgar si quiere o no participar en este estudio. Para ello lea esta hoja informativa con atención y nosotros le aclararemos las dudas que le puedan surgir después de la explicación. Además, puede consultar con las personas que considere oportuno.

Debe saber que su participación en este estudio es voluntaria y que puede decidir no participar o cambiar su decisión y retirar el consentimiento en cualquier momento, sin que por ello se altere la relación con su médico ni se produzca perjuicio alguno en su tratamiento.

En algunos casos y dependiendo de la edad, las personas que han sufrido un herpes zoster desarrollan dolor en la zona afectada con diferente intensidad, se piensa que entre un 15 y un 50% dependiendo de la edad.

El ensayo clínico al que se le invita a participar pretende determinar si la administración de gabapentina en las primeras 72h del inicio del herpes zoster y durante 5 semanas reduce el número de personas que sufren dolor después del episodio de herpes zoster, ni el médico ni usted sabrán cuál es el tratamiento que va a recibir, para ello la mitad de los pacientes recibirán el tratamiento con gabapentina y la otra mitad recibirán una capsula con las mismas características que la de gabapentina pero que no contiene sustancia farmacológicamente activa, igual que si usted se tomara un caramelo.

El estudio tiene una duración de 12 semanas, usted tendrá que acudir a 4 visitas con su médico y una visita realizada con una persona del estudio. En este estudio participarán 134 pacientes.

Si usted recibe el tratamiento activo (gabapentina) y los resultados son positivos, usted y otras personas con herpes zoster pueden que no desarrollen secuelas de dolor en la zona afectada, aunque también es posible que no obtenga ningún beneficio para su salud. Si usted recibe el placebo, al igual que un caramelo, no le producirá ni beneficio ni perjuicio alguno.

La gabapentina es un fármaco comercializado y se utiliza para el tratamiento de la epilepsia y del dolor neuropático, en los estudios de este fármaco han demostrado que es un fármaco que puede ser utilizado con seguridad en personas, sin embargo en estos estudios algunas personas desarrollaron efectos no deseados como: somnolencia, mareos vértigo e hinchazón en las extremidades, la gabapentina es un agente anticonvulsivante que actúa sobre el sistema nervioso central y puede producir somnolencia, vértigo, u otros síntomas relacionados. Aunque sólo sean de intensidad leve o moderada, estos efectos adversos pueden ser potencialmente peligrosos para los pacientes que conducen o manejan maquinaria, particularmente hasta el momento en que la experiencia individual del paciente esté consolidada.

Debe aconsejarse a los pacientes que se abstengan de conducir o manejar maquinaria hasta que se conozcan los efectos de gabapentina, así mismo la ingesta de alcohol o fármacos puede agravar algunos de los efectos secundarios de gabapentina relacionados con el SNC p. Ej. somnolencia, ataxia.

Usted no tendrá que pagar por los medicamentos del estudio.

Su médico recibirá una compensación económica por su participación en este estudio y ha declarado si existe o no conflicto de intereses.

El tratamiento, la comunicación y la cesión de los datos de carácter personal de todos los sujetos participantes se ajustará a lo dispuesto en la Ley Orgánica 15/1999, de 13 de diciembre, de protección de datos de carácter personal, y en su reglamento de desarrollo. De acuerdo con lo que establece la legislación mencionada, usted puede ejercer los derechos de acceso, modificación, oposición y cancelación de datos, para lo cual deberá dirigirse a su médico del estudio.

Sus datos serán tratados informáticamente y se incorporarán a un fichero automatizado de datos de carácter personal cuyo responsable es (*Alfonso Leiva Rus*), que ha sido registrado en la Agencia Española de Protección de Datos.

Sus datos recogidos para el estudio estarán identificados mediante un código y solo su médico del estudio y colaboradores podrán relacionar dichos datos con usted y con su historia clínica. Por lo tanto, su identidad no será revelada a persona alguna salvo en caso de urgencia médica o requerimiento legal.

Sólo se transmitirán a terceros y a otros países, previa notificación a la Agencia Española de Protección de Datos, los datos recogidos para el estudio que en ningún caso contendrán información que le pueda identificar directamente, como nombre y apellidos, iniciales, dirección, nº de la seguridad social, etc. En el caso de que se produzca esta cesión, será para los mismos fines del estudio descrito y garantizando la confidencialidad como mínimo con el nivel de protección de la legislación vigente en nuestro país.

El acceso a su información personal quedará restringido al médico del estudio/colaboradores, autoridades sanitarias, al Comité de Ética de la Investigación de las Illes Balears y personal autorizado, cuando lo precisen para comprobar los datos y procedimientos del estudio, pero siempre manteniendo la confidencialidad de los mismos de acuerdo con la legislación vigente.

Si usted decide retirar el consentimiento para participar en este estudio, ningún dato nuevo será añadido a la base de datos, si bien los responsables del estudio podrán seguir utilizando la información recogida sobre usted hasta ese momento, a no ser que usted se oponga expresamente.

También debe saber que usted puede ser retirado del estudio en caso de que los responsables del estudio lo consideren oportuno, ya sea por motivos de seguridad, por cualquier acontecimiento adverso que se produzca por la medicación en estudio o porque consideren que no está cumpliendo con los procedimientos establecidos. En cualquiera de los casos, usted recibirá una explicación adecuada del motivo que ha ocasionado su retirada del estudio.

Si usted es retirado del estudio, por alguno de los motivos expresados, su médico le prescribirá un tratamiento adecuado a su enfermedad.

Al firmar la hoja de consentimiento adjunta, se compromete a cumplir con los procedimientos del estudio que se le han expuesto.

Para cualquier duda se puede poner en contacto con:

Unidad de Investigación Gerencia de atención Primaria de Mallorca:

Telf. 971 175884

**16. ANEXO 2**

**CONSENTIMIENTO INFORMADO**

Caso nº: ____________

Título del ensayo:

Ensayo clínico, aleatorizado, doble-ciego para evaluar la eficacia y seguridad de la gabapentina frente a placebo en la prevención de la neuralgia postherpetica

Yo, _________________________________________________________

(Nombre y apellidos)

1. He leído la hoja de información del estudio.
2. He podido hacer preguntas sobre el estudio.
3. He recibido suficiente información sobre el estudio.
4. He hablado con ___________________________________

(Nombre médico investigador)

1. Comprendo que mi participación es voluntaria.
2. Se que puedo retirarme del estudio cuando quiera, sin tener que dar explicaciones y sin que esto repercuta en mis atenciones médicas.

Doy libremente la conformidad para participar en el estudio.

Firma del paciente Firma del médico responsable

Nombre del Paciente______________ Nombre del médico_________

Fecha: Fecha:

Investigador principal del estudio:

Manual Rullán García

CS Pollença.

Tlf: 971533041

[mrullan@ibsalut.caib.es](mailto:mrullan@ibsalut.caib.es)

**17. ANEXO 3**

**HOJA DE INFORMACION Y COMPROMISO PARA MÉDICOS COLABORADORES.**

Apreciado compañero,

Te invitamos a participar en un proyecto de investigación cuyo objetivo es estudiar la **efectividad de la gabapentina en la reducción del porcentaje de pacientes que sufren dolor tras un episodio de herpes Zoster, en pacientes mayores de 50 años**.

Si decides participar en el estudio, los pacientes que decidan participar serán seleccionados de forma aleatoria al grupo de tratamiento con gabapentina o placebo.

Los datos identificativos de los pacientes permanecerán anonimizados para el centro coordinador y los datos personales y clínicos de los pacientes se incluirán en una base de datos para ser analizados estadísticamente sin que en ningún momento se revele su identidad. El estudio **cumplirá con los principios de la Declaración de Helsinki y con la legislación vigente en materia de Ensayos Clínicos**. Toda la información recogida tendrá un carácter estrictamente confidencial y los datos se utilizarán de acuerdo con la Ley Orgánica de Protección de Datos de carácter personal LO15/1999 y la ley europea 95/46/CE.

Por tu colaboración en el estudio se te entregará un certificado de participación en el mismo.

Con la firma de este documento manifiestas que **no existe conflicto de intereses** entre tu participación en el estudio y tus deberes asistenciales con tus pacientes y que la inclusión de estos en el estudio no perjudicará en ningún caso sus intereses.

Así mismo te comprometes **a recoger, registrar y notificar los datos de forma correcta y garantizar su veracidad**, también a facilitar la monitorización de los datos registrados durante el tiempo de seguimiento y a guardar los datos del estudio por el período de tiempo que establece la ley.

Si después de recibir esta información accedes a participar, por favor firma por duplicado esta autorización y conserva una de las copias para ti.

Si tienes cualquier duda, puedes contactar con los responsables del proyecto cuyos datos constan a pie de página.

**Nombre del médico Fecha Firma**

**Unidad de Investigación Gerencia de Atención Primaria de Mallorca:**

Telf 971 175884, o

Investigador prinicipal del estudio:

Manual Rullán García

CS Pollença.

Tlf: 971533041

[mrullan@ibsalut.caib.es](mailto:mrullan@ibsalut.caib.es)

1. **18. ANEXO 4**
2. **COMPROMISO DE CONFIDENCIALIDAD DEL INVESTIGADOR**
3. Yo, Don/Dña. **……………………………………………………………………….**, con DNI nº **……………………….**, voy a participar en el Proyecto y Ensayo clínico, aleatorizado, doble-ciego para evaluar la eficacia y seguridad de la gabapentina frente a placebo en la prevención de la neuralgia postherpetica en calidad de médico colaborador como investigador/a de campo.
4. Por este motivo, y sólo con la finalidad señalada en los objetivos del Proyecto,
5. **ME COMPROMETO** a cumplir con la legislación vigente en cuanto a la confidencialidad de los datos de los participantes y la custodia de la documentación recogida en las fichas de inclusión de casos y los cuadernos de recogida de datos del estudio.
6. Fdo. **……………………….**
7. En **……………………………**, a **…..** de **………………….** de 2013

# 19. ANEXO 5

Adoptada por la
18ª Asamblea Médica Mundial, Helsinki, Finlandia, junio 1964
y enmendada por la
29ª Asamblea Médica Mundial, Tokio, Japón, octubre 1975
35ª Asamblea Médica Mundial, Venecia, Italia, octubre 1983
41ª Asamblea Médica Mundial, Hong Kong, septiembre 1989
48ª Asamblea General Somerset West, Sudáfrica, octubre 1996
52ª Asamblea General, Edimburgo, Escocia, octubre 2000
Nota de Clarificación, agregada por la Asamblea General de la AMM, Washington 2002
Nota de Clarificación, agregada por la Asamblea General de la AMM, Tokio 2004
59ª Asamblea General, Seúl, Corea, octubre 2008
64ª Asamblea General, Fortaleza, Brasil, octubre 2013

### Introducción

1.         La Asociación Médica Mundial (AMM) ha promulgado la Declaración de Helsinki como una propuesta de principios éticos para investigación médica en seres humanos, incluida la investigación del material humano y de información identificables.

La Declaración debe ser considerada como un todo y un párrafo debe ser aplicado con consideración de todos los otros párrafos pertinentes.

2.         Conforme al mandato de la AMM, la Declaración está destinada principalmente a los médicos. La AMM insta a otros involucrados en la investigación médica en seres humanos a adoptar estos principios.

### Principios generales

3.         La Declaración de Ginebra de la Asociación Médica Mundial vincula al médico con la fórmula “velar solícitamente y ante todo por la salud de mi paciente”, y el Código Internacional de Etica Médica afirma que: “El médico debe considerar lo mejor para el paciente cuando preste atención médica”.

4.         El deber del médico es promover y velar por la salud, bienestar y derechos de los pacientes, incluidos los que participan en investigación médica. Los conocimientos y la conciencia del médico han de subordinarse al cumplimiento de ese deber.

5.         El progreso de la medicina se basa en la investigación que, en último término, debe incluir estudios en seres humanos.

6.         El propósito principal de la investigación médica en seres humanos es comprender las causas, evolución y efectos de las enfermedades y mejorar las intervenciones preventivas, diagnósticas y terapéuticas (métodos, procedimientos y tratamientos). Incluso, las mejores intervenciones probadas deben ser evaluadas continuamente a través de la investigación para que sean seguras, eficaces, efectivas, accesibles y de calidad.

7.         La investigación médica está sujeta a normas éticas que sirven para promover y asegurar el respeto a todos los seres humanos y para proteger su salud y sus derechos individuales.

8.         Aunque el objetivo principal de la investigación médica es generar nuevos conocimientos, este objetivo nunca debe tener primacía sobre los derechos y los intereses de la persona que participa en la investigación.

9.         En la investigación médica, es deber del médico proteger la vida, la salud, la dignidad, la integridad, el derecho a la autodeterminación, la intimidad y la confidencialidad de la información personal de las personas que participan en investigación.  La responsabilidad de la protección de las personas que toman parte en la investigación debe recaer siempre en un médico u otro profesional de la salud y nunca en los participantes en la investigación, aunque hayan otorgado su consentimiento.

10.       Los médicos deben considerar las normas y estándares éticos, legales y jurídicos para la investigación en seres humanos en sus propios países, al igual que las normas y estándares internacionales vigentes. No se debe permitir que un requisito ético, legal o jurídico nacional o internacional disminuya o elimine cualquiera medida de protección para las personas que participan en la investigación establecida en esta Declaración.

11.       La investigación médica debe realizarse de manera que reduzca al mínimo el posible daño al medio ambiente.

12.       La investigación médica en seres humanos debe ser llevada a cabo sólo por personas con la educación, formación y calificaciones científicas y éticas apropiadas. La investigación en pacientes o voluntarios sanos necesita la supervisión de un médico u otro profesional de la salud competente y calificado apropiadamente.

13.       Los grupos que están subrepresentados en la investigación médica deben tener un acceso apropiado a la participación en la investigación.

14.       El médico que combina la investigación médica con la atención médica debe involucrar a sus pacientes en la investigación sólo en la medida en que esto acredite un justificado valor potencial preventivo, diagnóstico o terapéutico y si el médico tiene buenas razones para creer que la participación en el estudio no afectará de manera adversa la salud de los pacientes que toman parte en la investigación.

15.       Se debe asegurar compensación y tratamiento apropiados para las personas que son dañadas durante su participación en la investigación.

### Riesgos, Costos y Beneficios

16.       En la práctica de la medicina y de la investigación médica, la mayoría de las intervenciones implican algunos riesgos y costos.

La investigación médica en seres humanos sólo debe realizarse cuando la importancia de su objetivo es mayor que el riesgo y los costos para la persona que participa en la investigación.

17.       Toda investigación médica en seres humanos debe ser precedido de una cuidadosa comparación de los riesgos y los costos para las personas y los grupos que participan en la investigación, en comparación con los beneficios previsibles para ellos y para otras personas o grupos afectados por la enfermedad que se investiga.

Se deben implementar medidas para reducir al mínimo los riesgos. Los riesgos deben ser monitoreados, evaluados y documentados continuamente por el investigador.

18.       Los médicos no deben involucrarse en estudios de investigación en seres humanos a menos de que estén seguros de que los riesgos han sido adecuadamente evaluados y de que es posible hacerles frente de manera satisfactoria.

Cuando los riesgos que implican son más importantes que los beneficios esperados o si existen pruebas concluyentes de resultados definitivos, los médicos deben evaluar si continúan, modifican o suspenden inmediatamente el estudio.

### Grupos y personas vulnerables

19.       Algunos grupos y personas sometidas a la investigación son particularmente vulnerables y pueden tener más posibilidades de sufrir abusos o daño adicional.

Todos los grupos y personas vulnerables deben recibir protección específica.

20.       La investigación médica en un grupo vulnerable sólo se justifica si la investigación responde a las necesidades o prioridades de salud de este grupo y la investigación no puede realizarse en un grupo no vulnerable. Además, este grupo podrá beneficiarse de los conocimientos, prácticas o intervenciones derivadas de la investigación.

### Requisitos científicos y protocolos de investigación

21.       La investigación médica en seres humanos debe conformarse con los principios científicos generalmente aceptados y debe apoyarse en un profundo conocimiento de la bibliografía científica, en otras fuentes de información pertinentes, así como en experimentos de laboratorio correctamente realizados y en animales, cuando sea oportuno. Se debe cuidar también del bienestar de los animales utilizados en los experimentos.

22.       El proyecto y el método de todo estudio en seres humanos deben describirse claramente y ser justificados en un protocolo de investigación.

El protocolo debe hacer referencia siempre a las consideraciones éticas que fueran del caso y debe indicar cómo se han considerado los principios enunciados en esta Declaración. El protocolo debe incluir información sobre financiamiento, patrocinadores, afiliaciones institucionales, posibles conflictos de interés e incentivos para las personas del estudio y la información sobre las estipulaciones para tratar o compensar a las personas que han sufrido daños como consecuencia de su participación en la investigación.

En los ensayos clínicos, el protocolo también debe describir los arreglos apropiados para las estipulaciones después del ensayo.

### Comités de ética de investigación

23.       El protocolo de la investigación debe enviarse, para consideración, comentario, consejo y aprobación al comité de ética de investigación pertinente antes de comenzar el estudio. Este comité debe ser transparente en su funcionamiento, debe ser independiente del investigador, del patrocinador o de cualquier otro tipo de influencia indebida y debe estar debidamente calificado. El comité debe considerar las leyes y reglamentos vigentes en el país donde se realiza la investigación, como también las normas internacionales vigentes, pero no se debe permitir que éstas disminuyan o eliminen ninguna de las protecciones para las personas que participan en la investigación establecidas en esta Declaración.

El comité tiene el derecho de controlar los ensayos en curso. El investigador tiene la obligación de proporcionar información del control al comité, en especial sobre todo incidente adverso grave. No se debe hacer ninguna enmienda en el protocolo sin la consideración y aprobación del comité. Después que termine el estudio, los investigadores deben presentar un informe final al comité con un resumen de los resultados y conclusiones del estudio.

### Privacidad y confidencialidad

24.       Deben tomarse toda clase de precauciones para resguardar la intimidad de la persona que participa en la investigación y la confidencialidad de su información personal.

### Consentimiento informado

25.       La participación de personas capaces de dar su consentimiento informado en la investigación médica debe ser voluntaria. Aunque puede ser apropiado consultar a familiares o líderes de la comunidad, ninguna persona capaz de dar su consentimiento informado debe ser incluida en un estudio, a menos que ella acepte libremente.

26.       En la investigación médica en seres humanos capaces de dar su consentimiento informado, cada participante potencial debe recibir información adecuada acerca de los objetivos, métodos, fuentes de financiamiento, posibles conflictos de intereses, afiliaciones institucionales del investigador, beneficios calculados, riesgos previsibles e incomodidades derivadas del experimento, estipulaciones post estudio y todo otro aspecto pertinente de la investigación. El participante potencial debe ser informado del derecho de participar o no en la investigación y de retirar su consentimiento en cualquier momento, sin exponerse a represalias. Se debe prestar especial atención a las necesidades específicas de información de cada participante potencial, como también a los métodos utilizados para entregar la información.

Después de asegurarse de que el individuo ha comprendido la información, el médico u otra persona calificada apropiadamente debe pedir entonces, preferiblemente por escrito, el consentimiento informado y voluntario de la persona. Si el consentimiento no se puede otorgar por escrito, el proceso para lograrlo debe ser documentado y atestiguado formalmente.

Todas las personas que participan en la investigación médica deben tener la opción de ser informadas sobre los resultados generales del estudio.

27.       Al pedir el consentimiento informado para la participación en la investigación, el médico debe poner especial cuidado cuando el participante potencial está vinculado con él por una relación de dependencia o si consiente bajo presión. En una situación así, el consentimiento informado debe ser pedido por una persona calificada adecuadamente y que nada tenga que ver con aquella relación.

28.       Cuando el participante potencial sea incapaz de dar su consentimiento informado, el médico debe pedir el consentimiento informado del representante legal. Estas personas no deben ser incluidas en la investigación que no tenga posibilidades de beneficio para ellas, a menos que ésta tenga como objetivo promover la salud del grupo representado por el participante potencial y esta investigación no puede realizarse en personas capaces de dar su consentimiento informado y la investigación implica sólo un riesgo y costo mínimos.

29.       Si un participante potencial que toma parte en la investigación considerado incapaz de dar su consentimiento informado es capaz de dar su asentimiento a participar o no en la investigación, el médico debe pedirlo, además del consentimiento del representante legal. El desacuerdo del participante potencial debe ser respetado.

30.       La investigación en individuos que no son capaces física o mentalmente de otorgar consentimiento, por ejemplo los pacientes inconscientes, se puede realizar sólo si la condición física/mental que impide otorgar el consentimiento informado es una característica necesaria del grupo investigado. En estas circunstancias, el médico debe pedir el consentimiento informado al representante legal. Si dicho representante no está disponible y si no se puede retrasar la investigación, el estudio puede llevarse a cabo sin consentimiento informado, siempre que las razones específicas para incluir a individuos con una enfermedad que no les permite otorgar consentimiento informado hayan sido estipuladas en el protocolo de la investigación y el estudio haya sido aprobado por un comité de ética de investigación. El consentimiento para mantenerse en la investigación debe obtenerse a la brevedad posible del individuo o de un representante legal.

31.       El médico debe informar cabalmente al paciente los aspectos de la atención que tienen relación con la investigación. La negativa del paciente a participar en una investigación o su decisión de retirarse nunca debe afectar de manera adversa la relación médico-paciente.

32.       Para la investigación médica en que se utilice material o datos humanos identificables, como la investigación sobre material o datos contenidos en biobancos o depósitos similares, el médico debe pedir el consentimiento informado para la recolección, almacenamiento y reutilización. Podrá haber situaciones excepcionales en las que será imposible o impracticable obtener el consentimiento para dicha investigación. En esta situación, la investigación sólo puede ser realizada después de ser considerada y aprobada por un comité de ética de investigación.

### Uso del placebo

33.       Los posibles beneficios, riesgos, costos y eficacia de toda intervención nueva deben ser evaluados mediante su comparación con las mejores intervenciones probadas, excepto en las siguientes circunstancias:

Cuando no existe una intervención probada, el uso de un placebo, o ninguna intervención, es aceptable; o

cuando por razones metodológicas científicamente sólidas y convincentes, sea necesario para determinar la eficacia y la seguridad de una intervención el uso de cualquier intervención menos eficaz que la mejor probada, el uso de un placebo o ninguna intervención.

Los pacientes que reciben cualquier intervención menos eficaz que la mejor probada, el placebo o ninguna intervención, no correrán riesgos adicionales de daño grave o irreversible como consecuencia de no recibir la mejor intervención probada.

Se debe tener muchísimo cuidado para evitar abusar de esta opción.

### Estipulaciones post ensayo

34.       Antes del ensayo clínico, los auspiciadores, investigadores y los gobiernos de los países anfitriones deben prever el acceso post ensayo a todos los participantes que todavía necesitan una intervención que ha sido identificada como beneficiosa en el ensayo. Esta información también se debe proporcionar a los participantes durante el proceso del consentimiento informado.

### Inscripción y publicación de la investigación y difusión de resultados

35.       Todo estudio de investigación con seres humanos debe ser inscrito en una base de datos disponible al público antes de aceptar a la primera persona.

36.       Los investigadores, autores, auspiciadores, directores y editores todos tienen obligaciones éticas con respecto a la publicación y difusión de los resultados de su investigación. Los investigadores tienen el deber de tener a la disposición del público los resultados de su investigación en seres humanos y son responsables de la integridad y exactitud de sus informes. Todas las partes deben aceptar las normas éticas de entrega de información. Se deben publicar tanto los resultados negativos e inconclusos como los positivos o de lo contrario deben estar a la disposición del público. En la publicación se debe citar la fuente de financiamiento, afiliaciones institucionales y conflictos de intereses. Los informes sobre investigaciones que no se ciñan a los principios descritos en esta Declaración no deben ser aceptados para su publicación.

### Intervenciones no probadas en la práctica clínica

37.       Cuando en la atención de un paciente las intervenciones probadas no existen u otras intervenciones conocidas han resultado ineficaces, el médico, después de pedir consejo de experto, con el consentimiento informado del paciente o de un representante legal autorizado, puede permitirse usar intervenciones no comprobadas, si, a su juicio, ello da alguna esperanza de salvar la vida, restituir la salud o aliviar el sufrimiento. Tales intervenciones deben ser investigadas posteriormente a fin de evaluar su seguridad y eficacia. En todos los casos, esa información nueva debe ser registrada y, cuando sea oportuno, puesta a disposición del público.

**20.** **ANEXO 6**

**NOTIFICACIÓN ACONTECIMIENTOS ADVERSOS**

**NOTIFICACION DE ACONTECIMIENTOS ADVERSOS GRAVES**

I. INFORMACION DE ACONTECIMIENTOS

1. INICIALES PACIENTE 2. FECHA NACIMIENTO

3. FECHA INICIO ACONTECIMIENTO

4. MARCAR TODAS LAS CASILLAS RELACIONADAS CON EL ACONTECIMIENTO ADVERSO:

( ) EXITUS

( ) HOSPITALITZACIÓN O PROLONGACIÓN DE LA HOSPITALIZACIÓN

( ) INCAPACIDAD PERSISTENTE

( ) AMENAZA PARA LA VIDA

5. ACONTECIMIENTO ADVERSO:

II. INFORMACION SOBRE EL FARMACO

1. FÁRMACO SOSPECHO (INCLUIR NOMBRE GENÉRICO):

2. ¿HA DESAPARECIDO DESPUÉS DE LA INTERRRUPCIÓN DE LA ADMINISTRACION DEL FÁRMACO? ________________________________________________________________________

3. DOSIS DIARIA: _______________________ 4. VÍA DE ADMINISTRACIÓN: __________________

5. INDICACIÓN DEL FÁRMACO: _________________________________________________________

6. FECHAS DE ADMINISTRACION: DESDE / / HASTA / /

7. ¿HA REAPARECIDO EL ACONTECIMIENTO DESPUES DE REINTRODUIR EL TRATAMIENTO? ____________________________________________________________________________________

III. FÁRMACOS CONCOMITANTES

FARMACOS CONCOMITANTES Y FECHAS DE ADMINISTRACION

IV. NOMBRE, DIRECCIÓN Y TELÉFONO DEL INVESTIGADOR:

V. DESCRIPCIÓN DEL ACONTECIMIENTO E HISTORIA CLÍNICA

DESCRIPCIÓN DEL ACONTECIMIENTO Y Hª CLÍNICA RELEVANTE (INCLUIR DATOS DE LABORATORIO, DIAGNÓSTICOS, ALERGIAS, ETC)

FECHA DEL INFORME:
