# Supplementary material for: Efficacy of gabapentin for the prevention of postherpetic neuralgia in patients with acute herpes zoster: A double blind, randomized controlled trial
Source: PLoS One. 2019 Jun 5;14(6):e0217335. doi: 10.1371/journal.pone.0217335 (PMC6550400; doi:10.1371/journal.pone.0217335)
Supplement: S3 Appendix — (DOCX) [file pone.0217335.s003.docx]

**Study Protocol**

**Randomized, double-blind clinical trial to evaluate the efficacy and safety of gabapentin versus placebo in the prevention of postherpetic neuralgia**

**PI12_01813**

**Version** **10/02/2014**

**1. SUMMARY**

**1.1.** **Type of request**

Pharmacological clinical trial, Phase IV-III

**1.2.** **Promoter identifier**

Primary Care Management Mallorca

C / Reina Esclaramunda nº 9

07003 Palma de Mallorca

Phone 971175897

**1.3.** **Title of the clinical trial**

"Randomized, double-blind clinical trial to evaluate the efficacy and safety of gabapentin versus placebo in the prevention of postherpetic neuralgia"

**1.4.** **Principal Investigator: work center address**

Manuel Rullán García

[mrullan@ibsalut.caib.es](mailto:mrullan@ibsalut.caib.es)

CS Pollença

C / Bisbe Desbach, s / n

07460 Pollença

Telephone 971 738227

**1.5.** **Centers where the trial is planned**

 Majorca:

1. CS Pollença
2. CS Calvià
3. CS Camp Redó
4. CS Inca
5. CS Lluchmajor
6. CS Manacor
7. CS Platja Palma
8. CS Son Gotleu
9. CS Son Pisà
10. CS Son Serra-La Vileta
11. CS Arquitecte Bennasar
12. CS Soller
13. CS Coll d'en Rabassa
14. CS Rafal
15. CS Portocristo
16. CS Cala D'or
17. CS Graduate School
18. CS Trencadors

**1.6.** **Name and qualification of the person responsible for monitoring**

 To be determined.

**1.7.** **Intervention**

Treatment with gabapentin will be increased until an optimal dose or maximum tolerated dose will be reached; therefore, the dose escalation guideline will be carried out as indicated in the product's technical sheet. Treatment can be initiated either escalating the dose, as described in Table 1 or by administering 300 mg three times a day (TID) on Day 1. Subsequently, based on the patient's individual response and tolerability, the dose can be increased by 300 mg / day every 2-3 days up to a maximum dose of 1800 mg / day.

**Table 1:** Dose scheme (1^st^ week):

| Dose | Day 1 | Day 2 | Day 3 | Day 4 | Day 5 | Day 6 | Day 7 |
| --- | --- | --- | --- | --- | --- | --- | --- |
| Morning |  |  | 300mg | 3 00mg | 3 00mg | 600mg | Optimal dose |
| Lunch |  | 300mg | 300mg | 300mg | 600mg | 600mg | Optimal dose |
| Bedtime | 300mg | 300mg | 300mg | 6 00mg | 6 00mg | 600mg | Optimal dose |

 * Based on the patient's individual tolerability, the ceiling daily dose will be 1800mg / day, otherwise each patient will reach their optimal dose.

The treatment will be maintained for 4 weeks and then the dose will be reduced over a week to avoid possible "withdrawal effect"(anxiety, insomnia, nausea, pain, sweating).

**Table 2**: Scheme of dose tapering (5^th^ week).

| Dose | Day 30 | Day31-32 | Day33 | Day34 | Day35 | Day36 | Day37 |
| --- | --- | --- | --- | --- | --- | --- | --- |
| Morning | 600mg | 300mg | 300mg | 300mg |  |  | 0mg |
| Lunch | 600mg | 600mg | 300mg | 300mg | 300mg |  |  |
| Bedtime | 600mg | 600mg | 600mg | 300mg | 300mg | 300mg |  |

  * Example of dose tapering based on a ceiling daily dose of 1800mg / day

**1.8.** **Phase of the clinical trial**

Phase IV-III.

**1.9.** **Aims**

To evaluate the efficacy of an optimal dose of gabapentin added to the usual treatment -- valacyclovir and analgesics as needed -- in reduction of PHN at 12 weeks in patients more than 50 years-old who have moderate to severe pain

**1.10.** **Design**

We designed a multi-center, parallel, randomized, double-blind, placebo-controlled trial.

**1.11.** **Main outcome**

The main outcome measure will be the incidence of PHN at 12 weeks, defined as an average daily VAS pain score of 0.

**1.13.** **Study population and total number of patients**

Patients older than 50 years diagnosed with herpes zoster and with moderate-severe pain. One hundred and thirty-four patients will be included.

**1.14.** **Treatment duration**

A 5 weeks gabapentin or placebo treatment.

**1.15.** **Scheduled schedule**

The overall duration of the study will be 3 years.

**2. GENERAL INFORMATION**

**2.1. Trial identification**

**Title**

"Randomized, double-blind clinical trial to evaluate the efficacy and safety of gabapentin versus placebo in the prevention of postherpetic neuralgia"

**2.2. Type of clinical trial**

A multi-center, parallel, randomized, double-blind, placebo-controlled, phase IV-III clinical trial.

**2.3. Description of the intervention**

Treatment with gabapentin at optimal dose or placebo.

**2.4. Data related to the developer**

Primary Care Management Mallorca

C / Reina Esclaramunda nº 9

07003 Palma de Mallorca

Phone 971175897

**2.5. Identification of the monitor**

To be determined.

**2.6. Data from the trial investigators**

**Principal investigator**

Manuel Rullán García

[mrullan@ibsalut.caib.es](mailto:mrullan@ibsalut.caib.es)

CS Pollença

C / Bisbe Desbach, s / n

07460 Pollença

Telephone 971 738227

**Investigators and Healthcare Centers the trial will be conducted**

Martí Cladera Cifre

CS Pollença

Patricia Lorente Montalvo

CS Calvià

Catalina Comas Pol

CS Camp Redó

Maria Antònia Mir Pons

CS Inca

Tomeu Aulet

Biel Lliteras

CS Lluchmajor

Maria José González Bals

CS Manacor

Apol·lònia Cifre Socias

CS Platja Palma

Francisca Bestard

CS Son Gotleu

Salvador Gestoso Gayá

CS Son Pisà

Mª Antonia Roca

CS Son Serra-La Vileta

Antoni Jover Palmer

CS Arquitecte Bennasar

Mª Antonia Bosch

CS Coll dén Rabassa

Guillen More

CS Soller

Francisca Comas

CS Rafal

Diana Garau

CS Portocristo

Luis Lopez

CS Cala D'or

Teresa Rosselló

CS Graduate School

Biel Moragues

CS Trencadors

**2.7. Expected duration of the trial**

The expected duration of the trial is 3 years.

**3. JUSTIFICATION AND OBJECTIVES**

**3.1. Introduction**

Herpes Zoster (HZ) is a self-limiting disease, characterized by a vesicular dermal eruption distributed in the region of a cutaneous dermatome, usually unilateral, associated with or without neuropathic pain. The acute HZ infection is produced by the reactivation of the varicella-zoster virus, which has remained latent in a dorsal ganglion from the infantile infection, known as varicella. After reactivation, the virus extends centrally and peripherally from the dorsal ganglion, producing intense inflammation of the skin, peripheral nerves, nerve root and occasionally the spinal cord (1).

Postherpetic neuralgia (NPH) is considered its most frequent complication, although there is no consensus on its exact definition: for some authors is pain that persists after the healing of the acute skin rash, while others define it as the pain that appears and lasts at least one month after the onset of the vesicles or a minimum of 3-4 months-after the onset of the eruption. Despite these different definitions, pain resolution models suggested that the most appropriate definition is the persistence of pain 90 days after the onset of HZ (2-4).

This controversy makes it difficult to estimate its incidence, which can vary between 9 and 34% of patients with HZ, depending on the definition and the population studied, as well as the interpretation of the results of clinical trials (5).

In Spain, the incidence of HZ is 4.1 cases per 1,000 inhabitants per year for all ages (6). 9-10% of patients who have an HZ will have an NPH; 1.3% among those under 50, 36% of those over 60 and 50% of those over 70 (7). It is estimated that the annual cost attributable in the United Kingdom to the acute infection by HZ and NPH amounts to €108 million (8) and in Spain in Primary Care it has been estimated that the cost of HZ infection is €378 and NPH €821 per patient (9).

In the pathogenesis of NPH, it seems that central and peripheral mechanisms may be involved. A lesion of the spinal cord may occur, mainly of the posterior and anterior gray matter, which would explain the motor disorders that are sometimes associated with the disease. It is thought that in the NPH there would be a central hyperexcitability caused by the nociceptors during the acute phase, accompanied by an axonal lesion (10).

Despite the advances made in its treatment and prevention, PHN pain is difficult to control, therefore NPH continues to be a significant clinical problem. At present, several pharmacological strategies are available for the treatment of NPH, but their efficacy is limited, and it is associated with the appearance of adverse effects. This has led to the search for therapeutic alternatives applicable during the acute phase of the infection to prevent its onset.

The results of systematic reviews and clinicals trials indicate that the use of systemic antivirals during the first 72 h after the appearance of the rash accelerate healing and decrease the duration of pain; they also shorten the duration of PHN, but its effect on its incidence It is not well established (3,10). In this same line, the use of systemic corticosteroids in the acute phase has also been proposed as an alternative to prevent PHN but has only been shown to be effective in pain control and quality of life. Corticosteroids have been associated with a reduction in the incidence of PHN, but its use is controversial (3.5).

Currently the efficacy and safety of the new varicella- zoster vaccine has been evaluated in the Shingles Prevention Study, a randomized, multicenter, double-blind, placebo-controlled study that included more than 38,000 immunocompetent individuals of 60 or more years, with no history of HZ, with a mean follow-up of more than 3 years. The use of the vaccine was associated with a moderate reduction in the burden of the disease due to HZ (61.1%; p <0.001), as well as the incidence of PHN (66.5%, p <0.001). If the patient had developed HZ, the reduction in NPH was 12.46% versus 8.56%, a reduction of 31%. Currently the vaccine is approved for the prevention of HZ and NPH in adults 60 years old and older and more recently, with data only from immunogenicity, has also been approved for adults 50-59 years. Nevertheless, it is not known if the vaccine protection lasts beyond 4 years or if a revaccination is necessary. The use of vaccine use is currently not generalized (5,12).

On the other hand, given that intense pain during the acute phase of HZ is a known risk factor for the subsequent development of PHN, another feasible therapeutic strategy would be based on the reduction of the incidence of PHN once the HZ appeared by optimal pain control in this phase.

However, there is no a clear evidence of the efficacy of analgesic treatment in the acute phase of HZ and its effect on the incidence of PHN. Among the drugs studied during the acute phase of HZ are amitriptyline and gabapentin. The efficacy of amitriptyline and other antidepressants is well established to treat PHN pain, but the effectiveness of its use in the prevention of PHN has been evaluated in a single randomized, placebo-controlled study in a few patients (n = 72). Although the results were favorable -reduced the incidence of PHN by 50 %, the existence in the study of important methodological defects makes interpretation of the results difficult (12).

On the other hand, in an exploratory study published in 2005 to evaluate the efficacy of gabapentin administered in a single dose (900 mg) versus placebo, a reduction in pain intensity was shown in 66% of patients, compared to 33% in the control group, and a decrease in severity and area affected by allodynia. The authors indicated that a best pain control associated with the use of gabapentin during the acute phase could associate with a reduction in the incidence of PHN, but no new studies have been conducted on this issue (13).

Gabapentin acts at the level of the α2δ subunit of the calcium-dependent channels and reduces the release of neurotransmitters, that in animal models have been shown to be the mediating mechanism of its analgesic effect (14). Its mechanism of action would be based on the attenuation of the central sensitization or limitation of other central structural and functional changes that appear after intense nociceptor inputs such as damage on the gamma-aminobutyric acid inhibitory pathway.

Recently, an experimental study without a control group has been published; it is an open clinical trial with a single treatment arm to evaluate the efficacy of gabapentin treatment (initial dose of 300 mg / day up to a maximum of 1800 mg / d) and valaciclovir (1 g / 8h for 7 days) for the prevention of PHN in patients with acute HZ. The study included 133 patients > 50 years (mean age 64.6 years) immunocompetent with a time > 72 hours from the appearance of the first symptoms and moderate pain (score on the VAS scale> 4). The incidence of PHN at weeks 12, 16 and 24 were 20.3%, 18.0%, and 9.8% respectively (15).

  Although several pharmacological strategies are available for the treatment of PHN once it is established, its limited efficacy and the frequent occurrence of adverse effects make it necessary to search for therapeutic approaches during the acute phase of the infection to prevent its appearance. Given that severe pain during the acute phase of HZ is a risk factor for the subsequent development of PHN, one would expect that an optimal control of pain in this phase would be associated with a lower incidence of PHN.

Gabapentin is a neuromodulator whose analgesic effect in the treatment of pain in acute HZ and NPH is well established (16,17). It is also a drug with extensive experience of use and its benefit / risk balance and good tolerance is known; although it can cause various side effects, such as drowsiness, ataxia, diplopia, vertigo and nystagmus, they are very rare, mild and easily reverse by decreasing the dose or by making a progressive introduction; in addition, its interaction with other drugs is minimal, which facilitates its management. It is a drug that is not metabolized in the liver and eliminated by the kidney and barely binds to plasma proteins (less than 3%), therefore it has a good safety profile.  The penetration of the blood-brain barrier is quite good, which facilitates its analgesic action (18).

One of the key points for the treatment of HZ is the antiviral therapy within the first 72 hours after the onset of symptoms. In this sense, we believe that recruitment is more feasible in primary care practices. In our region (Balearic Island, Spain), the incidence of new cases of HZ diagnosed in primary care was 2200 cases in 2010, of which 1400 were aged 50 years or older.

The objective of this study of patients older than 50 years with moderate or severe pain from HZ is to assess the efficacy of gabapentin added to the usual treatment (valacyclovir and analgesics as needed) on reducing acute pain and preventing PHN at 12 weeks (19).

**3.2. Objectives and Hypothesis.**

1. **Main objective**

To evaluate the efficacy of an optimal dose of gabapentin added to the usual treatment -- valacyclovir and analgesics as needed -- in reduction of PHN at 12 weeks in patients more than 50 years-old who have moderate to severe pain.

Secondary objectives

To evaluate the efficacy of an optimal dose of gabapentin added to the usual treatment -- valacyclovir and analgesics as needed -- in reduction of PHN at 12 weeks in patients more than 50 years-old who have moderate to severe pain in terms of:

- Percentage of patients without PHN (defined as score 0 on the VAS scale of pain) at 6 weeks after the onset of acute symptoms.

- Percentage of patients with a reduction> 50% of the score on the VAS scale of pain at 6 and 12 weeks.

- Reduction of the incidence of neuropathic pain measured by the DN-4 scale.

- Improvement in the score of the SF-12 scale.

- To evaluate the safety of gabapentin treatment in terms of percentage of patient that report Adverse Events (AA).

**B) Main hypothesis**

Treatment with gabapentin at optimal dose (defined as maximum tolerated dose) for 5 weeks (4 weeks + 1 week of tapering), in the acute phase of HZ, added to the usual treatment (antiviral-valaciclovir and analgesic treatment) compared to usual treatment + gabapentin placebo will reduce by at least 25% the incidence of PHN at 12 weeks (5 weeks treatment + 7 weeks without treatment) in patients> 50 years with moderate-severe pain

**4. TYPE OF STUDY AND DESIGN**

**4.1. Design and type of control**

Clinical trial, multicenter, double-blind, parallel randomized of two treatment arms. The control group will receive placebo treatment of gabapentin at optimal doses. The trial registration will be made in the "International Standard Randomized Controlled Trial" http://www.controlled-trials.com/.

**4.2. Randomization process**

A sequence of random numbers will be generated by the Epidat 3.1. The investigators, after patient signed the informed consent [(IC), annex 2) will make a telephone call to the Research Unit of the Mallorca Primary Care Management to receive the randomization code for every patient. Every patient will have an assigned unique identification code.

The sequence of random numbers will be performance by blocks of 6. The date of randomization, the patient identification code and the assigned treatment arm will be recorded.

**4.3 Masking:**

The investigators, the patient and the statistician will remain blind to the treatment received by the patient.

To assess the effectiveness of the masking, the investigator, the patient and the statistician must choose which treatment group they believe belongs to each patient according to the following categories of mutually exclusive responses: placebo, gabapentin or do not know / no answer.

**5. SUBJECTS SELECTION**

**5.1. Inclusion and exclusion criteria**

**A) inclusion criteria**

To be included in the study, participants must meet all of the following inclusion criteria:

- Patients over 50 years old.

- Patients with uncomplicated HZ, within the first 72h since the rash onset, with moderate to severe pain (pain assessment on the VAS scale ≥ 4).

**B) exclusion criteria.**

- Patients treated with gabapentin, pregabalin, or tricyclic antidepressants

- Patients with a diagnosis of acute liver failure, hypersensitivity to active substance or to any of the excipients, or diagnosis of moderate or chronic renal failure

- Patients with evidence of cutaneous or visceral dissemination of the HZ (more than 20 lesions outside the adjacent dermatome) or ophthalmic involvement

- Patients with immunosuppressive or immunomodulatory treatment (interferon) in the last 4 weeks.

- Diagnosis of immunodeficiency in the last 3 months.

- Herpes zoster vaccination.

Treatment with tricyclic antidepressants or systemic corticosteroids will not be allowed during the trial period.

**5.2. Sample size**

The sample size has been calculated according to the main efficacy variable. The percentage incidence of PHN in patients in the valaciclovir + placebo group is expected to be at least 45% (Helgason S, Petursson G, Gudmundsson S, Sigurdsson JA. Prevalence of postherpetic neuralgia after a first episode of herpes zoster: prospective study with long term follow up BMJ 2000 Sep 30; 321 (7264): 794-6.) and in the valaciclovir + gabapentin group of 20.3% ( Lapolla W, Digiorgio C, Haitz K, Magel G, Mendoza N, et al. incidence of postherpetic neuralgia after combination treatment with gabapentin and valacyclovir in patients with herpes zoster: open-label study Arch Dermatol 2011; 147 (8): 901-7 ), We have calculated a follow-up loss rate of 20%; an alpha risk of 5% and a beta risk of 20%. Therefore, it is necessary to recruit 134 patients (67 subjects in each treatment arm).

**5.3.** **Recruitment**

The recruitment period will take place between June 2013 and June 2015.

Patient will be recruited on a scheduled visit with the GP or in the out of hours primary care centers.

• Scheduled visit with the GP: the research team will hold sessions to the centers of the recruited investigators. GPs will be explained the nature of the study and asked to refer to the investigator, who will investigate, to all eligible patient, the inclusion and exclusion criteria, invite to participate in the study and initiate the treatment with valacyclovir. Once the patient signed informed consent the patient will be randomized to gabapentin or placebo.

- Out of hours primary care centers. We will hold session to GPs in the out of hours centers and they will be asked to initiate valaciclovir treatment; the eligible patient will then be referred to the collaborating investigator with a scheduled visit. If the patient agrees to participate in the study and signs the IC, will afterward receive gabapentin or placebo.

.

**Training:** All the investigators participating in the study will receive basic training on the study and questionnaires filling. The training will be given by a research team with experience in the development of clinical trials. Investigator will be given the investigator’ manual with detailed explanation of the procedure and the methodology to be followed.

**5.4*.* Criteria for withdrawal of patients from the trial or analysis.**

**Important:** If the patient decides to abandon the study, patients should continue with the most appropriate treatment according to the opinion of the investigator; investigator will ask the patient consent for make an appointment for the the final visit. These patients cannot be included in any other research trial and the intervention will not be applied at any time.

Patients will only be considered to discontinue the trial if there is a loss of follow-up after exhausting all means of contact or if the patient explicitly expresses an intention to discontinue the trial and no longer attend scheduled visits.

Subsequent events must be communicated throughout the study.

Investigator will be asked to determine the reason(s) for which the patient stops attending the concerted visits or is discontinued from the trial. This information should be recorded in the corresponding section of the Case Report Form (CRF) and in the final sheet. If a patient leaves the study, all means of collecting information related to the main endpoints should be attempted.

**5.5.** **Quality control of the study development:**

Each center will be monitored before the center recruits any patient. The protocol, the investigator's manual, the CRFs, the procedures for obtaining informed consent will be reviewed. Two monitoring visits will be made to each center, in order to compare the data recorded in the CRFs with the data of the original documents and computerized clinical history.

The computerized clinical history should state that a project visit  PI12_01813 has been carried out " Clinical, randomized, double-blind trial to evaluate the efficacy and safety of gabapentin versus placebo in the prevention of postherpetic neuralgia" and record the dose that the patient is taking, baseline and final VAS scale score, dates of the visits, withdrawal of the study if it occurs, and date of completion of the study. This information will be contrasted by the external monitor of the study as quality control.

A pilot study will be conducted with 6 investigators that will include two patients each to know the degree of acceptance of the protocol by health professionals and patients.

***5*.6. Recruitment period**

The recruitment period will be 18 months.

**6. DESCRIPTION OF THE INTERVENTION**

The treatment of acute Herpes Zoster (HZ) includes the treatment of the dermatological lesions, antiviral treatment and pain control. The intervention for the participating patients will include these aspects according to the HZ current recommendations of treatment. (Fisterra Guide of Herpes zoster and post herpetic neuralgia updated on 03/05/2011. Available at: http://www.fisterra.com/guias-clinicas/herpes-zosterneuralgia-post-herpetica/ [Consulted on February 22, 2012] Fashner J, Bell AL. Herpes Zoster and Postherpetic Neuralgia: Prevention and Management. Am Fam Physician. 2011; 83: 1432-1437**).**

Treatment of dermatological lesions: The main objective is to prevent bacterial infection and rubbing pain. Adequate hygiene of the skin will be carried out to avoid a secondary bacterial infection; it is recommended to wear loose clothing to reduce friction and associated pain; the use of topical antivirals is not recommended.

Antiviral treatment: The results of various meta-analyzes and clinical trials indicate that the use of systemic antivirals during the first 72 h within the rash onset, accelerate healing and decrease the duration of pain; it also shortens the duration of PHN, although its effectiveness in reducing the incidence of PHN is not established. Its use is recommended if it is started within 72 hours since the rash onset. In our trial, as the time of the rash is an inclusion criteria, all patients will receive antiviral treatment.

Although any of the antivirals authorized for the treatment of HZ would be indicated, Valaciclovir 1g / 8 h will be administered during the trial for 7 days in both groups. Valacyclovir decreases the duration of neuropathic pain more rapidly than acyclovir (average days of pain: Valaciclovir 38-48 days vs. 51 days acyclovir). In addition, the results of a randomized clinical trial that compared the efficacy of valaciclovir with acyclovir, suggest that valaciclovir may be slightly more effective in pain resolution and in the prevalence of PHN (acyclovir group 25.7%, versus 18.6% in valaciclovir at 6 months). Therefore, the choice of valaciclovir as an antiviral in the trial will require the evaluation of the effect of gabapentin under the most unfavorable conditions.

Analgesic treatment: In both groups the following "analgesic ladder" proposed by the WHO will be followed:

First Step: paracetamol (NSAIDs appear to have modest efficacy in the acute neuralgic pain of Herpes Zoster). Step Two: combine a weak opioid analgesic with acetaminophen (e.g. codeine). Third Step: combine paracetamol with a powerful opioid analgesic (e.g. morphine).

Treatment with gabapentin or placebo: The patients included in the trial will receive the usual medical treatment for acute HZ infection and will be randomized to receive gabapentin or placebo.

Treatment with gabapentin will be increased until the optimal dose (maximum tolerated dose will be reached; therefore, the dose escalation guideline will be carried out as indicated in the product's technical sheet, FT).

The collaborating GP must keep in mind that as all anticonvulsants, gabapentin acts on the central nervous system and may cause drowsiness, dizziness or other related symptoms. Even if they are only of mild or moderate intensity, these adverse effects can be potentially dangerous for patients who drive or operate machinery, particularly until the time when the patient's individual experience is consolidated. Patients should be advised to refrain from driving or operating machinery until they know the effects of gabapentin, as well as the intake of alcohol or drugs can aggravate some of the side effects of gabapentin related to the CNS, e.g. drowsiness, ataxia.

Treatment can be initiated either escalating the dose, as described in Table 1 or by administering 300 mg three times a day (TID) on Day 1. Subsequently, based on the patient's individual response and tolerability, the dose can be increased by 300 mg / day every 2-3 days up to a maximum dose of 1800 mg / day.

Table 1: Dose scheme (1^st^ week):

| Dose | Day 1 | Day 2 | Day 3 | Day 4 | Day 5 | Day 6 | Day 7 |
| --- | --- | --- | --- | --- | --- | --- | --- |
| Morning |  |  | 300mg | 3 00mg | 3 00mg | 600mg | Optimal dose |
| Lunch |  | 300mg | 300mg | 300mg | 600mg | 600mg | Optimal dose |
| Bedtime | 300mg | 300mg | 300mg | 6 00mg | 6 00mg | 600mg | Optimal dose |

 * Based on the patient's individual tolerability, the ceiling daily dose will be 1800mg / day, otherwise each patient will reach their optimal dose.

The treatment will be maintained for 4 weeks from the beginning of the table and then the dose will be reduced over a week to avoid possible "withdrawal effect"(anxiety, insomnia, nausea, pain, sweating).

**Table 2**: Scheme of dose tapering (5^th^ week).

| Dose | Day 30 | Day31-32 | Day33 | Day34 | Day35 | Day36 | Day37 |
| --- | --- | --- | --- | --- | --- | --- | --- |
| morning | 600mg | 300mg | 300mg | 300mg |  |  | 0mg |
| Late | 600mg | 600mg | 300mg | 300mg | 300mg |  |  |
| Night | 600mg | 600mg | 600mg | 300mg | 300mg | 300mg |  |

 * Example of dose tapering based on a ceiling daily dose of 1800mg / day

**7. TIMELINE**

**7.1. Visits and outcomes**

- Screening or baseline visit: The study investigator will offer to the eligible patients, who meet the inclusion criteria of the study and do not incur any of the exclusion criteria, to participate in the study; the patient information sheet and informed consent will be also given.

In this first visit or baseline visit, all the patients included in the trial will be evaluated and the following data will be registered in the CRF:

* Review of the inclusion and exclusion criteria of the study.

* Baseline evaluation on the VAS scale.

* Evaluation of the SF-12 scale of quality of life.

* Sociodemographic data (age, sex and origin of the patient).

* Concomitant diseases: any disease that is present at the beginning of the trial.

* Concomitant medication records: any medication the patient takes during the trial including the selection period. Any change in the concomitant medication must be recorded at each follow-up visit.

* Delivery of antiviral medication and gabapentin according to the dosage schedule established in the technical file. Every patient will be reminded that it must bring the medication bottle to the final visit.

-Visit 1 of treatment. Week 1

*The investigators will deliver to the included patients the antiviral and gabapentin medication or placebo, according to the dosing schedule established in the data sheet; instructions for trial capsule’ counting will be also provided, and the patient will be informed that he must register the intake of the study drug daily.

*Any new concomitant diseases not presented in the previous visit and any new prescribed medication will be recorded.

*Any adverse event will be registered, and the record information should include: description of the adverse event, start date, end date or if continuous, and the possible causality with the study drug.

-Visit 2 of treatment. Week 4

*The study investigator will prescribe the withdrawal of gabapentin or placebo, according to the technical data sheet; instructions for trial capsule’ counting for the withdrawal phase will be also provided, and the patient will be informed that he must register the intake of the study drug daily.

*Any new concomitant diseases not presented in the previous visit and any new prescribed medication will be recorded.

*Any adverse event will be registered, and the record information should include: description of the adverse event, start date, end date or if continuous, and the possible causality with the study drug.

-Visit 3 follow-up. Week 6.

*The researcher of the study will perform the study medication count and record the measure of adherence to treatment.

*It will evaluate the efficacy of short-term treatment, assessing the pain using visual analogue scale, the quality of life at 6 weeks using the SF-12 quality of life scale and it will record the quality of sleep using the MOS-Sleep scale.

*It will record the new concomitant diseases that have appeared and newly prescribed medication.

*Any new concomitant diseases not presented in the previous visit and any new prescribed medication will be recorded.

*Any adverse event will be registered, and the record information should include: description of the adverse event, start date, end date or if continuous, and the possible causality with the study drug.

Final follow-up visit. Week 12:

*The study investigator will evaluate the effectiveness of the treatment measuring the pain using a visual analogue scale.

*It will also evaluate the quality of life through the SF-12 scale of quality of life, the quality of sleep through the MOS-Sleep scale and will assess the patient neuropathic pain using the DN-4 neuropathic pain scale.

* Any new concomitant diseases not presented in the previous visit and any new prescribed medication, especially those prescribed for pain will be recorded.

*Any adverse event will be registered, and the record information should include: description of the adverse event, start date, end date or if continuous, and the possible causality with the study drug.

**7.2.** **Main dependent variable.**

Percentage of patients without PHN at the end of follow-up at 12 weeks (defined as score 0 on the Analog Visual Scale (VAS) of pain). The VAS scale will be used with a score of 0 to 10.

**7.3.** **Secondary dependent variables.**

- Percentage of patients without PHN at the follow-up visit at 6 weeks

- Percentage of patients without PHN at 6 weeks of onset of the acute condition defined as score 0 on the VAS scale of pain.

- Percentage of patients considered responders that improve the symptomatology of PHN at 6 and 12 weeks after the onset of the acute condition: We define responders to those patients who achieve a reduction of 50% or less on the VAS pain score. to basal.

·- Quality of life: using ShortForm-12 (SF-12 Spanish version ( [Med Care, 1996 Mar; 34 (3): 220-33.](https://translate.google.com/translate?hl=es&prev=_t&sl=auto&tl=en&u=http://www.ncbi.nlm.nih.gov/pubmed%3Fterm%3DMed%2520Care.%25201996%2520Mar%253B34%25283%2529%253A220-33.) ) ), a short form of SF-36® Heath Survey; all eight domains (physical functioning, role physical, bodily pain, general health, vitality, social functioning, role emotional and mental health) will be measured using a scale ranging from 0 to 100.,

- Sleep interference: the quality and quantity of sleep was measured by the Medical Outcomes Study Sleep Scale; this scale has been validated previously in Spanish and with 12 items, assessing the key constructs of sleep (Eur J Pain, 2007 Apr; 11 (3): 329-40.) In weeks 6 and 12,

-Patient with neuropathic pain by the Spanish version of the questionnaire DN4 (score on the scale > 3) (Health Qual Life Outcomes, 2007 Dec 4; 5: 66) , two-dimensional scale Pain quality and neurological exploration of the pain, which consists of 4 questions and 10 response items on the characteristics of pain .

- Impression scale of overall patient improvement  Spanish version ( [Int Urogynecol J Pelvic Floor Dysfunction, 2008 Aug; 19 (8): 1109-16](https://translate.google.com/translate?hl=es&prev=_t&sl=auto&tl=en&u=http://www.ncbi.nlm.nih.gov/pubmed%3Fterm%3D18360735) ) : consists of a single question that asks the patient to classify the relief obtained with the treatment that follows according to a Likert scale of seven points .

-Consumption of analgesics: In weeks 6 and 12 the analgesic consumption related to NPH will be evaluated.

All the variables of efficacy and quality of life will be collected through an interview with the patient.

- Security variable:

Incidence of adverse effects and severe AA. (sources: interview with the patient during follow-up visits to the study and clinical history of Primary Care).

**7.4. Independent variables**

 The **main independent variable**will be the treatment group (gabapentin or placebo).

**Independent variables:**

**-**Socio-demographic variables: age, sex and BMI. (source: interview and collection of anthropometric data at the baseline visit: age, sex, BMI.

- Percentage of adherence to the prescribed treatment: At the final visit, the patient will be invited to bring the bottle with gabapentin medication and a count will be made of the pills he has taken and those that have been prescribed.

- Presence of Diabetes Mellitus, autoimmune diseases and neuropathies.

(sources: interview and clinical history)

-Concomitant medication: analgesics, tricyclic antidepressants, opioids, HZ vaccination, etc. (sources: interview and clinical history).

**7.5. Pilot study**

 A pilot study will be conducted with 6 investigators that will include two patients each, in order to know the degree of acceptance of the protocol by health professionals and patients, checking the adequacy of the circuits in the detection of patients with herpes zoster, offering participation in the study in a period not exceeding 72h, communication with the research unit and subsequent randomization.

**8. ADVERSE EFFECTS**

Incidence of adverse events and severe AA. In a double-blind study in patients with neuropathic pain treated with gabapentin, somnolence, peripheral edema and asthenia appeared in a higher percentage in patients> 65 when compared with younger patients. Apart from these facts, clinical investigations in this age group do not indicate different adverse events from that observed in young patients.

Despite this, as there have not been systematic studies in patients older than 65 years and the trial foresees the inclusion of patients older than 50 years, information regarding these AAs will be systematically collected. (Gabapentin, technical data sheet of the product).

When gabapentin was prescribed, several adverse effects have been detected, such as:

Disorders of blood and lymphatic system:

Common: leukopenia.

Rare: thrombocytopenia.

Immune system disorders:

Rare: allergic reactions (for example, urticaria).

Metabolism and nutrition disorders:

Common: anorexia, increased appetite.

Psychiatric disorders:

Frequent: hostility, confusion and emotional instability, depression, anxiety, nervousness, abnormal thinking.

Rare: hallucinations.

Nervous system disorders:

Very common: drowsiness, dizziness, ataxia.

Common: seizures, hyperkinesia, dysarthria, amnesia, tremor, insomnia, headache, sensations such as paresthesia, hypesthesia, abnormal coordination, nystagmus and increase, decrease or absence of reflexes.

Rare: movement disorders (for example, choreoathetosis, dyskinesia, dystonia).

Eye disorders:

Common: visual disturbances such as amblyopia, diplopia.

Ear and labyrinth disorders:

Common: vertigo.

Rare: tinnitus.

Cardiac disorders:

Rare: palpitations

Vascular disorders:

Common: hypertension, vasodilation.

Respiratory, thoracic and mediastinal disorders:

Common: dyspnea, bronchitis, pharyngitis, cough, rhinitis.

Gastrointestinal disorders:

Common: vomiting, nausea, dental anomalies, gingivitis, diarrhea, abdominal pain, dyspepsia,

constipation, dry mouth or throat, flatulence.

Rare: pancreatitis.

Hepatobiliary disorders:

Rare: hepatitis, jaundice.

Disorders of the skin and subcutaneous tissue:

Common: facial, purple edema most often described as contusions resulting from physical trauma, rash, pruritus, acne.

Rare: Stevens-Johnson syndrome, angioedema, erythema multiforme, alopecia.

Musculoskeletal and connective tissue disorders:

Common: arthralgia, myalgia, back pain, spasms.

Renal and urinary disorders:

Uncommon: incontinence

Rare: acute renal failure.

Disorders of the reproductive system and breast:

Common: impotence.

General disorders and alterations in the place of administration:

Very frequent: fatigue, fever.

Common: peripheral or generalized edema, abnormal gait, asthenia, pain, malaise, flu syndrome.

Rare: reactions due to withdrawal syndrome (mainly anxiety, insomnia, nausea, pain, sweating), chest pain. Unexpected deaths have been reported, in which a causal relationship with treatment with gabapentin has not been established.

Complementary explorations:

Common: decreased white blood cell count, weight gain.

Rare: fluctuations of blood glucose in patients with diabetes, increased liver function tests.

Traumatic injuries and poisonings:

Common: accidental injuries, fracture, abrasion.

 In severe cases, acute pancreatitis and suicidal tendencies have been described during treatment with gabapentin.

Adverse events are classified as:

Mild: The adverse event related to the treatment with gabapentin does not modify the normal life of the patient.
Moderate: The adverse event related to the treatment with gabapentin alters the normal life of the patient (involves medical visit or sick leave) 
Serious: The adverse event related to treatment with gabapentin directly endangers the patient's life. 
Death: The patient is death because of treatment with gabapentin.

Serious adverse events are considered to be any serious or fatal withdrawal symptom and any cause of hospitalization or death, whether or not caused by treatment with gabapentin.

If a serious adverse event occurs, the record of serious adverse events should be filled out (Annex 6) and sent by fax before 48 hours to the coordinating center: 971175888.

**9. ETHICS**

This project has been submitted to the approval of the Ethical Committee of the Balearic Islands. The health professionals participating in the study will sign a document committing themselves to guarantee the confidentiality of the data of all the patients (annex 3, 4). Before initiating any procedure of the study, the informed consent of each patient must be obtained (annex 2). The patient information sheet will contain the necessary information (annex 1) and will be previously approved by CEIC. The consent will be kept with the documents of the investigator’s study. To obtain consent, the recommendations included in the Declaration of Helsinki (annex 5) will be followed and the investigators will sign a confidentiality document of the collected data.

**10. PRACTICAL CONSIDERATIONS**

**Organization of the study and responsibilities**

**10.1. Monitoring Committees**

**A) Executive Committee.**It will be formed by Dr. Manuel Rullán García, Dr. Joan Llobera Cànaves, will supervise the quality and methodological rigor of the data collection and analysis. It will also be responsible for developing a mechanism for transmitting information to the investigators of each center. It will evaluate the results of the monitoring and will be responsible for correcting the detected problems. It will also review and approve the presentations or publications made.

**B) Coordination Committee:**Dr. Manuel Rullán García and Alfonso Leiva Rus. They will be responsible for the uniform application of the protocol in the different health centers and to maintain the rhythm of inclusion, organize the dispatch and collection of material, coordination of the study staff.

**C) Safety Committee:**Will be formed by Dr. Manuel Rullan Garcia, Dr. Joan Llobera Cànaves; their role will be to receive, review and notify if necessary to the Spanish Agency of Medicines, serious adverse effects of the study. The opening of the randomization code will also be within its functions.

**10.2. Responsibilities**

Each investigators who decides to participate in the study, will be responsible for the recruitment of patients, the request and achievement of written informed consent, the application of the inclusion and exclusion criteria, the notification of inclusion to the coordinating center, the patient’ visit schedule and periodic evaluation according to the study protocol, of the collection and periodic sending of data to the coordinating center and of the communication of serious adverse effects.

The coordinating center of the study will be responsible for the planning of the study, the design and implementation of the data collection instruments, the statistical analysis, the edition of the operations and procedures manual, the organization of the training sessions, standardization and certification of the investigators, to organize the pilot study and its analysis, the subsequent edition of the manual and CRD if necessary, sending of questionnaires and study materials to the participating centers, to supervise and coordinate the collection of data (face-to-face monitoring in a sub-sample) of patients and data processing of all centers.

The general coordination will be in charge of the Research Unit of the Primary Care Department of Mallorca.

**11. STATISTICAL ANALYSIS**

**Global data management**

Every subject included in the study will be assigned a unique numerical identifier created with an algorithm based on the codes of the community of origin, the Health Center to which it belongs, the collaborating doctor and the case number.

The identifier will be present in each of the tables of the study. Here are the different data tables that will be included in the study:

1. Inclusion table. It will correspond to the data of the CRDs of visits -1 & -0.
2. Tracking table. They correspond to the data of the CRDs of visits -1, -2 and -3.
3. Final visit table. They correspond to the data of the CRDs of the visit -4.
4. Monitoring table. Record of the monitoring carried out for each of the included patients.

The ***Teleform***program has a verification module that guarantees the quality of the data entry. Nevertheless, 30% of the questionnaires will be entered manually and the data will be validated by checking the file concordance with the ***Epiinfo***program**.**The data in printed support will be stored locally and copies will be sent periodically to the coordinating center, where they will be processed and stored in magnetic support. Two backup copies will be made on magnetic tape of all the data entered each month. An IBM 3500 backup 400 Gb server appliance will be used.

All statistical analyzes will be carried out with the **SPSS for Windows v15**. The level of statistical significance is established at 5% bilaterally. The statistical analysis will be the responsibility of the Research Unit of the Primary Care Management of Mallorca.

For the statistical analysis, the SPSS program for Windows v.15:

- Descriptive analysis, labeling and filtering of data: evaluation of atypical and extreme values ('outliers´), detection and labeling of lost values and / or non-applicable values, description of the distribution of each of the variables.  Normality tests, scatter plots.

- Baseline comparative analysis: Comparison between sociodemographic characteristics between the intervention group and the control group, through the t-test and chi-square test. In case of non-fulfillment of normality assumptions, non-parametric tests will be applied.

- Final comparative analysis: Comparison of the clinical characteristics, of medication between the groups of placebo and treatment, using t-test and chi-square test. If the normality assumptions are not met, nonparametric tests will be applied. The clinical relevance of the intervention will be determined from the absence of pain at 12 weeks in the control and intervention groups and in the benefit / risk ratio of the intervention, the relative risk reduction (RRR), the absolute risk reduction (ARR) and the number of patients needed to treat will be calculated (NNT). A crude analysis will be performed by chi-square and adjusted by logistic regression of the baseline characteristics that show to be different in the control and intervention groups. All the analyzes will be carried out by intent to treat (`intention to treat '). The level of statistical significance is established at 5% bilaterally.

**12. DIFFICULTIES AND LIMITATIONS OF THE STUDY.**

  When including patients with moderate or intense pain, an effect of regression to the mean could occur, therefore there would be a tendency for these patients to refer less intense pain independently of the effect of gabapentin or placebo; however when comparing two groups we assume that this effect will occur in the two groups equally and therefore will not affect the estimation of the magnitude of the risk.

Gabapentin is a drug that can have adverse effects, and this may cause on one hand, a greater number of losses of follow-up in the active drug group; if these losses are more frequent in older patients, with more comorbidity or greater immunosuppression, a type I error can occur; to avoid it we opted for an intention-to-treat analysis.

On the other hand, patients taking the active drug could recognize that they are not taking placebo due to the presence of such adverse effects; to control the possible lack of blinding of the collaborating GP doing the follow-up visits and the patients, the number follow-up visits have been limited. The magnitude of the masking of the placebo will be assessed both in patient and collaborating GP; they will be asked to which treatment group they think belong.

**13. WORK CALENDAR**

**Project preparation: January-April 2013**

- Preparation of the CRDs, inclusion sheet and detailed drafting of the protocol.
- Review of the CRDs for all the subprojects and improvements.
- Recruitment collaborating GP for the trial.
- Registration ISRCTN

**Training and piloting: April -July 2013:**

- Training in research methodology in clinical trials for the included GPs.
- Piloting the questionnaires and rectifications (April 15)
- Second coordination meeting to prepare field work, collaborating GP's research manual and actions before protocol deviations.
- Contact Service Pharmacy of Son Espases Hospital, and purchase study medication and placebo.
- CEIC
- AEMPS registration

**Fieldwork: August 2013-April 2015:**

- Inclusion of patients and randomization.
- Baseline assessment and initiation of patient follow-up
- Reception of CRDs
- Data entry
- Research Team meeting
- Quality control, queries resolution.
- Monitoring
- Preparation of preliminary reports
- Annual reports FIS 2013/2014

**End: May -September 2015:**

- Analysis of data.
- Meeting with general report of the results, discussion of the results and planning of the preparation of articles.
- Dissemination of results.
- Memory.
- Final Fis 2015
- Bibliographic search and preparation of the manuscripts.
- Final memory.

**Table 1: Summary table of the tests and interventions to be carried out during the study**

| Basal visit: screening | Visit 1: Treatment | Visit 2: Treatment | Visit 3: Treatment | Visit 4: Final |
| --- | --- | --- | --- | --- |
| Week 0 | Week 1 | Week 4 | Week 6 | Week 12 |
| Investigator | Investigator | Investigator | Investigator | Investigator |
| Inclusion criteria: - Patients older than 50 years.  -Patients with an uncomplicated HZ diagnosis of less than 72h of evolution, with moderate to intense pain (pain score on the EVA scale ≥ 4). | Delivery of medication antiretroviral and gabapentin or placebo according to dosage pattern established in the technical data sheet. |  |  |  |
| Exclusion criteria: - Patients in habitual treatment with gabapentin, pregabalina or antidepressants triciclitos  -Patients with a diagnosis of severe liver failure, hypersensitivity to the active substance or to any of the Excipients, the Diagnostic of moderate or severe renal failure.  -Patients with evidence of cutaneous or visceral dissemination of HZ (more than 20 lesions outside the adjacent dermatome) or ophthalmic involvement  -Patients with immunosuppressive treatment in the last 3 months or immunomodulatory (interferon) in the last 4 weeks, diagnosis of  Immunodeficiency of any kind.  -Vaccination herpes zoster. |  |  |  |  |
| Demographic data (age, sex and origin of the patient) and anthropometric (weight and size) |  |  |  |  |
| Concomitant diseases | Register of new concomitant diseases | Register of new concomitant diseases | Register of new concomitant diseases | Register of new concomitant diseases |
| Concomitant medication registration | Concomitant medication register | Concomitant medication register | Concomitant medication register | Concomitant medication register |
| Randomization  Tel:(971175883 (76704)/ 659691565) Fax: 971175888. | Record of adverse events | Record of adverse events | Record of adverse events | Record of adverse events |
| Basal evaluation on the EVA scale.  And the SF-12 quality of life scale. |  |  | Baseline evaluation in the EVA scale, MOS-Sleep  And the SF-12 quality of life scale. | Baseline evaluation in the EVA scale, MOS-Sleep, the SF-12 scale of quality of life and DN4 scale. |
| Delivery bottle medication |  |  | Reminder medication for the next visit | Medication count |

**14. BIBLIOGRAPHY**

(1) González-Escalada J. R. Pregabalina en el tratamiento del dolor neuropático periférico. Rev. Soc. Esp. Dolor 2005;12: 169-180.

(2) Desmond RA, Weiss HL, Arani RB. Clinical applications for change-point analysis of herpes zoster pain. Journal of Pain and Symptom Management. 2002; 23(6): 510-6.

(3) Whitley RJ, Volpi A, McKendrick M, Van Wijck A, Oaklander AL. Management of herpes zoster and post-herpetic neuralgia now and in the future. Journal of clinical virology 2010;48:S20-S28.

(4) Arani RB, Soong SJ, Weiss HL, Wood MJ, Fiddian RJ et al. Phase specific analysis of herpes zoster associated pain data: a new statistical approch. Stat Med 2001;20:2429-39.

(5) López E, Agustí A. Prevención de la neuralgia postherpética. Med Clin (Barc). 2008;130(20):794-6.

(6) Cebrián-Cuenca AM, Díez-Domingo J, Rodríguez MS, Puig-Barberá J, Navarro-Pérez J; 'Herpes Zoster Research Group of the Valencian Community'. Epidemiology of herpes zoster infection among patients treated in primary care centres in the Valencian community (Spain). BMC Fam Pract. 2010 May 6;11:33.

(7) Helgason S, Petursson G, Gudmundsson S, Sigurdsson JA. Prevalence of postherpetic neuralgia after a first episode of herpes zoster: prospective study with long term follow up. BMJ. 2000 Sep 30;321(7264):794-6.

(8) Wahreham DW, Breuer J. Herpes Zoster. Clinical Review. BMJ 2007;334:1211-5.

(9) Cebrián-Cuenca AM, Díez-Domingo J, San-Martín-Rodríguez M, Puig-barbera J, Navarro-Pérez J et al. Epidemiology and cost of herpes zoster and postherpetic neuralgia among patients treated in primary care centers in valencian community of Spain. BMC Infectious Diseases 2011;11:302

(10) Qifu Li, Ning Chen, Jie Yang, Muke Zhou, Dong Zhou, Quanwei Zhang, Li He. Tratamiento antiviral para la prevención de la neuralgia posherpética (Revision Cochrane traducida). En: Biblioteca Cochrane Plus 2009 Número 3. Oxford: Update Software Ltd. Disponible en: http://www.update-software.com. (Traducida de The Cochrane Library, 2009 Issue 2 Art no. CD006866. Chichester, UK: John Wiley & Sons, Ltd.).

(11) Oxman MN, Levin MJ, Johnson GR, Schmader KE, Straus SE, Gelb LD, et al, and the Shingles Prevention Study Group. A vaccine to prevent herpes zoster and postherpetic neuralgia in older adults. N Engl J Med. 2005;352:2271-84.

(12) Bowsher D. The effects of pre-emptive treatment of post-herpetic neuralgia: a randomised, double-blind, placebocontrolled trial. J Pain Symptom Manage. 1997;13:327-31.

(13) Berry JD, Petersen KL. A single dose of gabapentin reduces acute pain and allodynia in patients with herpes zoster. Neurology. 2005;65:444-7.

(14) Tenser RB, Dworkin RH. Herpes zoster and the prevention of postherpetic neuralgia. Beyond antiviral therapy. Neurology 2005;65:349 -350.

(15) Lapolla W, Digiorgio C, Haitz K, Magel G, Mendoza N, et al.Incidence of postherpetic neuralgia after combination treatment w ith gabapentin and valacyclovir in patients with acute herpes zoster: open-label study. Arch Dermatol 2011;147(8):901-7

(16) Fashner J, Bell AL. Herpes Zoster and Postherpetic Neuralgia: Prevention and Management. Am Fam Physician. 2011;83:1432-1437.

(17) Guía Fisterra de Herpes zoster y neuralgia post herpética actualizada el 03/05/2011. Disponible en: http://www. fisterra.com/guias-clinicas/herpes-zoster-neuralgia-post-herpetica/ [Consultada el 22 de febrero de 2012].

(18) Gabapentina. Ficha técnica del producto. Disponible en AEMPS.

(19) Green CB. Prevent rather than treat postherpetic neuralgia by prescribing gabapentina earlier in patients with herpes zoster. Practice gaps. Arch Dermatool 2011;147:908.

**15. ANNEX 1**

**PATIENT INFORMATION SHEET**

**TITLE OF THE STUDY:** A randomized, double-blind clinical trial to evaluate the efficacy and safety of gabapentin versus placebo in the prevention of postherpetic neuralgia

**SPONSOR CODE:** PI12_01813

**SPONSOR:**Management of Primary Care Mallorca

**MAIN INVESTIGATOR:** Manuel Rullán García

**CENTER:**CS Pollença

**INTRODUCTION**

We are addressing you to inform about a research study in which you are invited to participate. The study has been approved by the Research Ethics Committee of the Balearic Islands and the Spanish Agency for Medicines and Health Products, in accordance with the legislation in force, and is carried out with respect to the principles set out in the Declaration of Helsinki and the standards of good clinical practice.

Our intention is that you receive the correct and enough information so that you can evaluate and judge whether you want to participate in this study. For this, please read this information sheet carefully and we will clarify any doubts that may arise after the explanation. In addition, you can consult with the people you consider appropriate.

You should know that your participation in this study is voluntary and that you can decide not to participate or change your decision and withdraw your consent at any time, without altering the relationship with your doctor or causing any harm to your treatment.

In some cases, and depending on the age, people who have suffered a herpes zoster can develop pain in the affected area of different intensity; it is thought that between 15 and 50% of them depending on age.

The clinical trial to which you are invited to participate aims to determine if the administration of gabapentin in the first 72h of the onset of shingles and for 5 weeks, reduces the number of people suffering from pain after the herpes zoster episode; neither the doctor nor you will know what treatment you will receive, for this half of the patients will receive treatment with gabapentin and the other half will receive a capsule with the same characteristics as gabapentin, but which does not contain pharmacologically active substance, just as if you will take a candy.

The study lasts 12 weeks and you will have to go to 4 visits with your doctor and one visit made with a person from the study. One hundred thirty-four patients will participate in this study.

If you receive active treatment (gabapentin) and the results are positive, you and other people with herpes zoster may not develop pain as a sequela in the affected area, although you may not get any benefit to your health. If you receive the placebo, like a candy, it will not cause you any benefit or harm.

Gabapentin is a marketed drug and is used for the treatment of epilepsy and neuropathic pain and this drug have shown in several studies that it is a drug that can be used safely in people, however in these studies some people developed unwanted effects as: drowsiness, dizziness and swelling in the extremities. Gabapentin is an anticonvulsant agent that acts on the central nervous system and can cause drowsiness, dizziness, or other related symptoms. Even if they are only of mild or moderate intensity, these adverse effects can be potentially dangerous for patients who drive or operate machinery, particularly until the patient's individual experience is consolidated.

Patients should be advised to refrain from driving or operating machinery until the effects of gabapentin are known, and the intake of alcohol or drugs may aggravate some of the CNS-related side effects of gabapentin, e.g. drowsiness, ataxia.

You will not have to pay for the study drugs.

Your GP will receive financial compensation for your participation in this study and has declared whether or not there is a conflict of interest.

The treatment, communication and transfer of personal data of all participating subjects will comply with the provisions of Organic Law 15/1999, of December 13, on the protection of personal data, and its regulations development. In accordance with the aforementioned legislation, you can exercise the rights of access, modification, opposition and cancellation of data, for which you should contact your study physician.

Your data will be processed electronically and will be incorporated into an automated personal data file which is responsible (*Alfonso Leiva Rus*), which has been registered with the Spanish Agency for Data Protection. Your study data will be identified by a code and only your physician and the research team will be able to relate this information to you and your medical history. Therefore, your identity will not be revealed to any person except in case of medical emergency or legal requirement.

Your data can only be transmitted to third parties and other countries, prior notification to the Spanish Agency for Data Protection and the data collected for the study in no case will contain information that can identify you directly, such as name and surnames, initials, address, number of the social security, etc. In the event that this transfer occurs, it will be for the same purposes of the study described and guaranteeing confidentiality at least with the level of protection of current legislation in our country.

Access to your personal information will be restricted to the investigator / research team, health authorities, the Research Ethics Committee of the Balearic Islands and authorized personnel, when they need to check the data and procedures of the study, but always maintaining confidentiality of them according to current legislation.

If you decide to withdraw consent to participate in this study, no new data will be added to the database, although those responsible for the study may continue to use the information collected about you up to that time, unless you expressly object to it.

You should also know that you can be removed from the study if the responsible for the study deems it appropriate, either for reasons of safety, for any adverse event that occurs due to the study medication or because they consider that you are not complying with the established procedures. In either case, you will receive an adequate explanation of the reason for your withdrawal from the study.

If you are removed from the study, for any of the reasons expressed, your physician will prescribe an appropriate treatment for your illness.

By signing the enclosed consent form, you agree to comply with the study procedures that have been presented to you.

For any questions you can contact:

Research Unit Primary Care Management of Mallorca:

Tel 971 175884

**16. Annex 2**

**INFORMED CONSENT**

Case no: ____________

Title of the essay:

 A randomized, double-blind clinical trial to evaluate the efficacy and safety of gabapentin versus placebo in the prevention of postherpetic neuralgia

I, _________________________________________________________

              (Name and surname)

1.      I have read the study information sheet.

2.      I was able to ask questions about the study.

3.      I have received enough information about the study.

4.      I have spoken with ___________________________________

                                                        (Medical investigator name)

5.      I understand that my participation is voluntary.

6       I know that I can withdraw from the study whenever I want, without having to give explanations and without this having an impact on my medical attention.

I freely give my consent to participate in the study.

Signature of the patient  Signature of the responsible physician

Patient's Name______________ Physician's Name _________

Date:                                                                  Date:

Principal investigator of the study:

Manual Rullán García

CS Pollença.

Tlf: 971533041

[mrullan@ibsalut.caib.es](mailto:mrullan@ibsalut.caib.es)

**17. ANNEX 3**

**INFORMATION SHEET AND COMMITMENT FOR COLLABORATING PHYSICIANS.**

Dear partner,

We invite you to participate in a research project whose objective is to study the **effectiveness of gabapentin in reducing the percentage of patients suffering from pain after an episode of herpes Zoster, in patients older than 50 years**.

If you decide to participate in the study, the patients who decide to participate will be randomly selected to the gabapentin or placebo treatment groups. The identification data of the patients will remain anonymous for the coordinating center and the personal and clinical data of the patients will be included in a database to be analyzed statistically without their identity being revealed at any time. The study **will comply with the principles of the Declaration of Helsinki and with the current legislation on Clinical Trials**. All information collected will be strictly confidential and the data will be used in accordance with the Organic Law of Protection of Personal Data LO15 / 1999 and European Law 95/46 / CE.

For your collaboration in the study you will be given a certificate of participation.

With the signing of this document you state that **there is no conflict of interest**between your participation in the study and your care duties with your patients and that the inclusion of these in the study will not harm your interests in any case.

You also agree **to collect, record and notify the data correctly and ensure its accuracy**, also to facilitate the monitoring of the data recorded during the monitoring time and save the study data for the period of time established by law.

If after receiving this information you agree to participate, please sign this authorization in duplicate and keep one of the copies for yourself.

If you have any questions, you can contact the people in charge of the project whose data is at the bottom of the page.

**Physician's name Date Signature**

**Research Unit Management of Primary Care of Mallorca:**

Tel 971 175884, or

Principal investigator of the study:

Manual Rullán García

CS Pollença.

Tlf: 971533041

[mrullan@ibsalut.caib.es](mailto:mrullan@ibsalut.caib.es)

**18. ANNEX 4**

**COMMITMENT OF CONFIDENTIALITY OF THE INVESTIGATOR**

I, Mr / Mrs ............................................ ......................................, with DNI no ........ ...................., I will participate in the project: "a randomized, double blind clinical trial to evaluate the efficacy and safety of gabapentin versus placebo in the prevention of postherpetic neuralgia" as a collaborating physician field investigator.

For this reason, and only with the guidance indicated in the objectives of this project,

I AGREE to comply with the legislation in force regarding the confidentiality of the data of the participants and the custody of the documentation collected in the files of inclusion of the cases and the notebooks of data collection of the study.

Signed ............................

In ................................., a ... ... of ........ .............. of 2013

**19. ANNEX 5**

**DECLARATION OF HELSINKI**

Adopted by the 18th WMA General Assembly, Helsinki, Finland, June 1964
and amended by the:
29th WMA General Assembly, Tokyo, Japan, October 1975
35th WMA General Assembly, Venice, Italy, October 1983
41st WMA General Assembly, Hong Kong, September 1989
48th WMA General Assembly, Somerset West, Republic of South Africa, October 1996
52nd WMA General Assembly, Edinburgh, Scotland, October 2000
53rd WMA General Assembly, Washington DC, USA, October 2002 (Note of Clarification added)
55th WMA General Assembly, Tokyo, Japan, October 2004 (Note of Clarification added)
59th WMA General Assembly, Seoul, Republic of Korea, October 2008
64th WMA General Assembly, Fortaleza, Brazil, October 2013

### Preamble

1.         The World Medical Association (WMA) has developed the Declaration of Helsinki as a statement of ethical principles for medical research involving human subjects, including research on identifiable human material and data.

The Declaration is intended to be read as a whole and each of its constituent paragraphs should be applied with consideration of all other relevant paragraphs.

2.         Consistent with the mandate of the WMA, the Declaration is addressed primarily to physicians. The WMA encourages others who are involved in medical research involving human subjects to adopt these principles.

### General Principles

3.         The Declaration of Geneva of the WMA binds the physician with the words, “The health of my patient will be my first consideration,” and the International Code of Medical Ethics declares that, “A physician shall act in the patient’s best interest when providing medical care.”

4.         It is the duty of the physician to promote and safeguard the health, well-being and rights of patients, including those who are involved in medical research. The physician’s knowledge and conscience are dedicated to the fulfilment of this duty.

5.         Medical progress is based on research that ultimately must include studies involving human subjects.

6.         The primary purpose of medical research involving human subjects is to understand the causes, development and effects of diseases and improve preventive, diagnostic and therapeutic interventions (methods, procedures and treatments). Even the best proven interventions must be evaluated continually through research for their safety, effectiveness, efficiency, accessibility and quality.

7.         Medical research is subject to ethical standards that promote and ensure respect for all human subjects and protect their health and rights.

8.         While the primary purpose of medical research is to generate new knowledge, this goal can never take precedence over the rights and interests of individual research subjects.

9.         It is the duty of physicians who are involved in medical research to protect the life, health, dignity, integrity, right to self-determination, privacy, and confidentiality of personal information of research subjects. The responsibility for the protection of research subjects must always rest with the physician or other health care professionals and never with the research subjects, even though they have given consent.

10.       Physicians must consider the ethical, legal and regulatory norms and standards for research involving human subjects in their own countries as well as applicable international norms and standards. No national or international ethical, legal or regulatory requirement should reduce or eliminate any of the protections for research subjects set forth in this Declaration.

11.       Medical research should be conducted in a manner that minimises possible harm to the environment.

12.       Medical research involving human subjects must be conducted only by individuals with the appropriate ethics and scientific education, training and qualifications. Research on patients or healthy volunteers requires the supervision of a competent and appropriately qualified physician or other health care professional.

13.       Groups that are underrepresented in medical research should be provided appropriate access to participation in research.

14.       Physicians who combine medical research with medical care should involve their patients in research only to the extent that this is justified by its potential preventive, diagnostic or therapeutic value and if the physician has good reason to believe that participation in the research study will not adversely affect the health of the patients who serve as research subjects.

15.       Appropriate compensation and treatment for subjects who are harmed as a result of participating in research must be ensured.

### Risks, Burdens and Benefits

16.       In medical practice and in medical research, most interventions involve risks and burdens.

Medical research involving human subjects may only be conducted if the importance of the objective outweighs the risks and burdens to the research subjects.

17.       All medical research involving human subjects must be preceded by careful assessment of predictable risks and burdens to the individuals and groups involved in the research in comparison with foreseeable benefits to them and to other individuals or groups affected by the condition under investigation.

Measures to minimise the risks must be implemented. The risks must be continuously monitored, assessed and documented by the researcher.

18.       Physicians may not be involved in a research study involving human subjects unless they are confident that the risks have been adequately assessed and can be satisfactorily managed.

When the risks are found to outweigh the potential benefits or when there is conclusive proof of definitive outcomes, physicians must assess whether to continue, modify or immediately stop the study.

### Vulnerable Groups and Individuals

19.       Some groups and individuals are particularly vulnerable and may have an increased likelihood of being wronged or of incurring additional harm.

All vulnerable groups and individuals should receive specifically considered protection.

20.       Medical research with a vulnerable group is only justified if the research is responsive to the health needs or priorities of this group and the research cannot be carried out in a non-vulnerable group. In addition, this group should stand to benefit from the knowledge, practices or interventions that result from the research.

### Scientific Requirements and Research Protocols

21.       Medical research involving human subjects must conform to generally accepted scientific principles, be based on a thorough knowledge of the scientific literature, other relevant sources of information, and adequate laboratory and, as appropriate, animal experimentation. The welfare of animals used for research must be respected.

22.       The design and performance of each research study involving human subjects must be clearly described and justified in a research protocol.

The protocol should contain a statement of the ethical considerations involved and should indicate how the principles in this Declaration have been addressed. The protocol should include information regarding funding, sponsors, institutional affiliations, potential conflicts of interest, incentives for subjects and information regarding provisions for treating and/or compensating subjects who are harmed as a consequence of participation in the research study.

In clinical trials, the protocol must also describe appropriate arrangements for post-trial provisions.

### Research Ethics Committees

23.       The research protocol must be submitted for consideration, comment, guidance and approval to the concerned research ethics committee before the study begins. This committee must be transparent in its functioning, must be independent of the researcher, the sponsor and any other undue influence and must be duly qualified. It must take into consideration the laws and regulations of the country or countries in which the research is to be performed as well as applicable international norms and standards but these must not be allowed to reduce or eliminate any of the protections for research subjects set forth in this Declaration.

The committee must have the right to monitor ongoing studies. The researcher must provide monitoring information to the committee, especially information about any serious adverse events. No amendment to the protocol may be made without consideration and approval by the committee. After the end of the study, the researchers must submit a final report to the committee containing a summary of the study’s findings and conclusions.

### Privacy and Confidentiality

24.       Every precaution must be taken to protect the privacy of research subjects and the confidentiality of their personal information.

### Informed Consent

25.       Participation by individuals capable of giving informed consent as subjects in medical research must be voluntary. Although it may be appropriate to consult family members or community leaders, no individual capable of giving informed consent may be enrolled in a research study unless he or she freely agrees.

26.       In medical research involving human subjects capable of giving informed consent, each potential subject must be adequately informed of the aims, methods, sources of funding, any possible conflicts of interest, institutional affiliations of the researcher, the anticipated benefits and potential risks of the study and the discomfort it may entail, post-study provisions and any other relevant aspects of the study. The potential subject must be informed of the right to refuse to participate in the study or to withdraw consent to participate at any time without reprisal. Special attention should be given to the specific information needs of individual potential subjects as well as to the methods used to deliver the information.

After ensuring that the potential subject has understood the information, the physician or another appropriately qualified individual must then seek the potential subject’s freely-given informed consent, preferably in writing. If the consent cannot be expressed in writing, the non-written consent must be formally documented and witnessed.

All medical research subjects should be given the option of being informed about the general outcome and results of the study.

27.       When seeking informed consent for participation in a research study the physician must be particularly cautious if the potential subject is in a dependent relationship with the physician or may consent under duress. In such situations the informed consent must be sought by an appropriately qualified individual who is completely independent of this relationship.

28.       For a potential research subject who is incapable of giving informed consent, the physician must seek informed consent from the legally authorised representative. These individuals must not be included in a research study that has no likelihood of benefit for them unless it is intended to promote the health of the group represented by the potential subject, the research cannot instead be performed with persons capable of providing informed consent, and the research entails only minimal risk and minimal burden.

29.       When a potential research subject who is deemed incapable of giving informed consent is able to give assent to decisions about participation in research, the physician must seek that assent in addition to the consent of the legally authorised representative. The potential subject’s dissent should be respected.

30.       Research involving subjects who are physically or mentally incapable of giving consent, for example, unconscious patients, may be done only if the physical or mental condition that prevents giving informed consent is a necessary characteristic of the research  group. In such circumstances the physician must seek informed consent from the legally authorised representative. If no such representative is available and if the research cannot be delayed, the study may proceed without informed consent provided that the specific reasons for involving subjects with a condition that renders them unable to give informed consent have been stated in the research protocol and the study has been approved by a research ethics committee. Consent to remain in the research must be obtained as soon as possible from the subject or a legally authorised representative.

31.       The physician must fully inform the patient which aspects of their care are related to the research. The refusal of a patient to participate in a study or the patient’s decision to withdraw from the study must never adversely affect the patient-physician relationship.

32.       For medical research using identifiable human material or data, such as research on material or data contained in biobanks or similar repositories, physicians must seek informed consent for its collection, storage and/or reuse. There may be exceptional situations where consent would be impossible or impracticable to obtain for such research. In such situations the research may be done only after consideration and approval of a research ethics committee.

### Use of Placebo

33.       The benefits, risks, burdens and effectiveness of a new intervention must be tested against those of the best proven intervention(s), except in the following circumstances:

Where no proven intervention exists, the use of placebo, or no intervention, is acceptable; or

Where for compelling and scientifically sound methodological reasons the use of any intervention less effective than the best proven one, the use of placebo, or no intervention is necessary to determine the efficacy or safety of an intervention

and the patients who receive any intervention less effective than the best proven one, placebo, or no intervention will not be subject to additional risks of serious or irreversible harm as a result of not receiving the best proven intervention.

Extreme care must be taken to avoid abuse of this option.

### Post-Trial Provisions

34.       In advance of a clinical trial, sponsors, researchers and host country governments should make provisions for post-trial access for all participants who still need an intervention identified as beneficial in the trial. This information must also be disclosed to participants during the informed consent process.

### Research Registration and Publication and Dissemination of Results

35.       Every research study involving human subjects must be registered in a publicly accessible database before recruitment of the first subject.

36.       Researchers, authors, sponsors, editors and publishers all have ethical obligations with regard to the publication and dissemination of the results of research. Researchers have a duty to make publicly available the results of their research on human subjects and are accountable for the completeness and accuracy of their reports. All parties should adhere to accepted guidelines for ethical reporting. Negative and inconclusive as well as positive results must be published or otherwise made publicly available. Sources of funding, institutional affiliations and conflicts of interest must be declared in the publication. Reports of research not in accordance with the principles of this Declaration should not be accepted for publication.

### Unproven Interventions in Clinical Practice

37.       In the treatment of an individual patient, where proven interventions do not exist or other known interventions have been ineffective, the physician, after seeking expert advice, with informed consent from the patient or a legally authorised representative, may use an unproven intervention if in the physician’s judgement it offers hope of saving life, re-establishing health or alleviating suffering. This intervention should subsequently be made the object of research, designed to evaluate its safety and efficacy. In all cases, new information must be recorded and, where appropriate, made publicly available.

**20. ANNEX 6**

**NOTICE OF ADVERSE EVENTS**

**NOTIFICATION OF SERIOUS ADVERSE EVENTS**

I. EVENT INFORMATION

1. patient initials

2. date of birth

3. date start event

4. mark all cases related to the adverse event:

              () death

              () hospitalization or prolongation of hospitalization

              () persistent disability

              () threat to life

5. adverse event:

II. DRUG INFORMATION

1. Suspected drug (include generic name):

2. Has it disappeared after interrupting drug administration?

 _______________________________________________________________

3. Daily dose: _______________________

4. Route of administration: __________________

5. Indication of the drug: _________________________________________________________

6. Administration dates: from / / until / /

7. Has the event reappeared after reintroducing the drug?

_______________________________________________________________

III. CONCOMITANT DRUGS

CONCURRENT DRUGS AND ADMINISTRATION DATES

IV. INVESTIGATOR’ NAME, ADDRESS AND TELEPHONE

V. DESCRIPTION OF THE EVENT AND CLINICAL HISTORY

Description of the event and relevant clinic (including laboratory data, diagnostics, allergies, etc.)

DATE OF THE REPORT:
